# Hybrid Hierarchical Heterostructures of Nanoceramic Phosphors as Imaging Agents for Multiplexing and Living Cancer Cells Translocation

**DOI:** 10.1021/acsabm.0c01417

**Published:** 2021-03-10

**Authors:** David G. Calatayud, Teresa Jardiel, Mara S. Bernardo, Vincenzo Mirabello, Haobo Ge, Rory L. Arrowsmith, Fernando Cortezon-Tamarit, Lorena Alcaraz, Josefa Isasi, Pablo Arévalo, Amador C. Caballero, Sofia I. Pascu, Marco Peiteado

**Affiliations:** † Department of Electroceramics, Instituto de Ceramica y Vidrio−CSIC, Kelsen 5, Campus de Cantoblanco, Madrid 28049, Spain; ‡ Department of Chemistry, University of Bath, Claverton Down, Bath BA2 7AY, United Kingdom; § Department of Inorganic Chemistry I, Universidad Complutense de Madrid, Madrid28040, Spain

**Keywords:** core−shell nanoparticles, cellular bioimaging, multiplexing probes, luminescence, nanophosphors

## Abstract

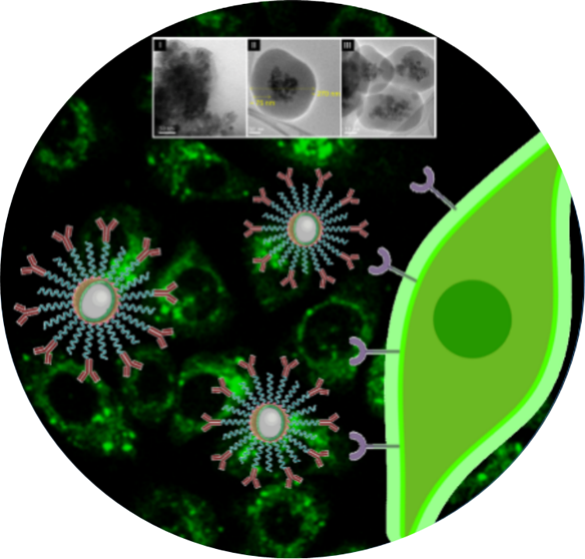

Existing fluorescent labels used in life sciences are based on organic compounds with limited lifetime or on quantum dots which are either expensive or toxic and have low kinetic stability in biological environments. To address these challenges, luminescent nanomaterials have been conceived as hierarchical, core–shell structures with spherical morphology and highly controlled dimensions. These tailor-made nanophosphors incorporate Ln:YVO_4_ nanoparticles (Ln = Eu(III) and Er(III)) as 50 nm cores and display intense and narrow emission maxima centered at ∼565 nm. These cores can be encapsulated in silica shells with highly controlled dimensions as well as functionalized with chitosan or PEG5000 to reduce nonspecific interactions with biomolecules in living cells. Confocal fluorescence microscopy in living prostate cancer cells confirmed the potential of these platforms to overcome the disadvantages of commercial fluorophores and their feasibility as labels for multiplexing, biosensing, and imaging in life science assays.

## Introduction

Recent advances in bioinspired fabrication approaches, which can combine nanomaterials self-assembly, molecular recognition, and soft matter chemistry processes, have led to sustainable methods to produce functional materials with nanometer precision.^1^ New synthetic methodologies, focused on the nano scale, have opened the door to new manufacturing processes that allow for accessible, large-scale, and sustainable materials production in terms of energy consumption and environmental footprint, and give rise to complex nanostructures of relevance to catalysis, energy conversion and storage, architecture, communications, or healthcare applications.^2−4^ New nanoparticles with functional and tunable optical properties are of relevance to the field of diagnostic and therapeutic nanomedicine,^5−8^ a currently emerging area with great potential to address current challenges related to the diagnosis and treatment of noncommunicable diseases such as cancer.^9^ Nanomedicine encompasses the wide range of nanoscale technologies aimed at revolutionizing the basis of disease through early diagnosis, treatment, and prevention of diseases, i.e., the medical application of nanotechnology ranging from nanomaterials design to nano biosensors and their applications.^5,10−15^ Molecular-level control for the assembly of functional nanoparticles has recently been used in drug design aiming to facilitate the selective binding of biological materials to inorganic substrates.^16,17^ Additionally, new methods of early detection and non-invasive post-diagnosis monitoring of cancer are in great demand as they can improve the survival rate,^18−20^ and it is here where nanomedicine and, particularly, its application to biomedical imaging, can become an essential tool: new near-infrared (NIR) absorbing and emitting nanoprobes for advances in single- and multiplexing arrays used in biosensing technologies are a holy grail, yet challenges remain regarding their synthesis, batch-to-batch reproducibility, size and shape control, biocompatibility, as well as the bio- and photophysical characterization. Biomedical imaging is a powerful diagnostic tool for personalized and targeted medicine.^21,22^ Industrial and academic research users in this sector require access to advanced and affordable monitoring tools and testing facilities that can also accelerate the development of new and safe medical technologies. Recent studies have demonstrated that the applications of fluorescent labels in biomedical imaging have the potential to address the current ca. €520 million medical diagnostics market.^23^

Photoluminescence spectroscopy and related imaging techniques^24^ remain the most widely used in biopsy diagnostics; additionally, fluorescence imaging can be used to track and evaluate the efficiency of drug release and it is complementary to photodynamic therapies. The fluorescence techniques applied to date employ a number of well-established organic molecules further functionalized to be directed to target cancer specifically, such as Rhodamine, derivatives of fluorescein, and more recently NIR-emitting cyanine dyes.^22^ The dominant immunoassays used in biosensing clinical, preclinical, and life sciences research are multiplexed ELISA, where different emitting fluorophores anchored onto supports and coated with specific antibodies are responsive for a high precision biomarker detection, additionally to Western Blot (WB), immunofluorescence microscopy (IF), immunohistochemistry (IHC), and flow cytometry (FC) which all have a requirement for enzymatic or fluorescence-based detection methodologies.^25^ The majority of the currently available immunoassays are either singleplex or low multiplex (2–4 channels) and in the latter, the extra channels are normally required for providing contextual information such as providing cellular context in imaging applications.

**Figure 1:**
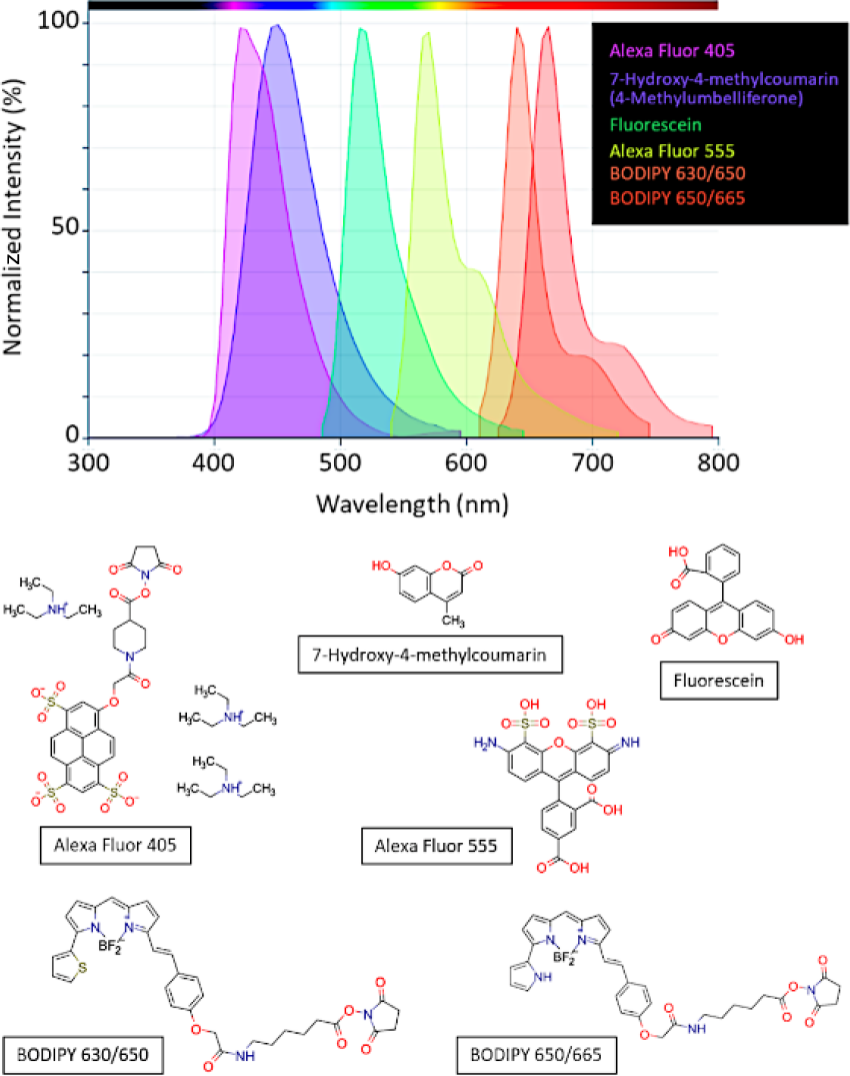
Fluorescence emission spectra and structures of the commercially available dyes: Alexa Fluor 405, 7-hydroxy-4-methylcoumarin (4-mehtylumbelliferone), Fluorescein, Alexa Fluor 555, BODIPY 630/650, and BODIPY 650/665.

Therefore, fluorescence-based detection remains the ideal choice for multiplexing in biosensing domains, and there are a range of well-studied fluorophores commercially available. The most popular ones include the fluorescein-based FITC, AlexaFluor, and BODIPY-families of commercial dyes, which have established roles in assays in the life sciences domain, used together or in combination with dye-conjugated primary and secondary antibodies available commercially as individual products (typical for FC and IF/IHC) or formulated with other reagents into kits (typical for ELISA). Moreover, fluorescent-labeled antibodies can be applied in the detection of ELISA, which is termed as fluorescence-linked immunosorbent assay (FLISA). Significant draw-backs yet exist around these systems particularly due to the broadness and frequently overlapping emission signals (Figure 1).^26^ Namely, signal detection normally occurs through an enzymatically processed organic fluorescent dye molecule with disadvantages linked to the stability, photobleaching, and broad emission ranges, which are all particularly limiting when used in a multiplex format. It is common to multiplex colors in IF and FC, where 4 channel experiments (using single photon excitation wavelengths of 350, 405, 488, and 543 nm) are relatively routine. The multiplex limit here is imposed by the ability to spectrally separate the fluorophores due to the wide emission peaks of these dyes.

There have been attempts to overcome these impediments to the performance of common organic dyes through the development of quantum dots, luminescent nanocrystalline materials based on nanoparticulate semiconductors, such as CdSe, CdTe, or CdS.^27^ These materials display tunable fluorescent spectra and single-wavelength-excited multicolor emissions, offering greater stability and brightness over organic fluorophores, but they also have some significant disadvantages. Color tuning is based on particle size, which limits their convenience for some applications, and they can even “blink” under certain conditions, which is not helpful for signal acquisition. In addition, the monodispersity of QDs must be ensured with rigorous synthesis qualifications based on the quantum confinement-induced size-dependent emissions, and those that are hydrophobic also require additional modifications to achieve water solubility. These two factors critically raise the cost of the QD-based bioimaging probes. Finally, most available quantum dots contain highly toxic metals (Cd, Se, Hg, etc.), which pose potential risks to human health and environment, thus further restricting their practical applications.^28^

Alternative materials approaches such as the use of inorganic luminescent nanoparticles are gaining increased attention to overcome the restrictive scenarios with current biosensing probes. Mostly based on doped oxide-based matrices, these materials, labeled nanophosphors, can show considerable advantages compared with quantum dots and organic dyes, including strong luminescence and low toxicity.^29,30^

They present higher photostability upon continuous excitation, which renders them highly advantageous as synthetic scaffolds in singleplex systems.^28^ Since the luminescence resulting from the metal ion dopants (activators) can cover a broad range of the optical spectra from the UV to the near-infrared regions, they can be simultaneously engineered to have specific optical properties with practical use in multiplex assays.^31^ In addition, their size similarity to biomolecules ensures their potential for the investigation of biological events by means of super-resolution microscopy techniques, in which a relatively fast acquisition is required while keeping photobleaching and toxicity acceptably low.^32^ Among the metal ions that can serve as activators of the luminescent response, lanthanide ions are particularly interesting due to their auspicious optical properties arising from their electronic configuration.

Lanthanides have recently been used as the materials scaffold of choice in the design and delivery of new inorganic luminescent materials: they display a partially filled 4f shell shielded by the filled 5s^2^ and 5p^6^ orbitals that lead to weak electron–phonon coupling and result in narrow and sharp emission lines of 4f-4f transitions.^30^ They do have a relatively low global intensity luminescence caused by the low absorptions of the parity forbidden Ln^3+^ 4f-4f transitions, however, this can be compensated for by using aluminate, phosphate, or vanadate matrices as host materials for the Ln^3+^ doping ions, eventually leading to an intense emission.^33^ This requires the ability to also engineer the nanophosphor systems with adaptable surface chemistries and explains why current research with these materials is focused both on obtaining high quantum efficiency values and long half-life excitation states, as well as on improving their physical and chemical stability. The principal draw-back for the widespread application of the nanophosphor technology in current molecular imaging techniques is the inherent difficulty to ensure an adequate binding of the luminescent nanomaterials to the biological species, surfaces, or material targets, and in allowing them to be used in low concentrations (i.e., in order to not interfere with the systems being studied).^34,35^

Design elements in the preparation of core–shell inorganic composites with stable emissive properties and tunable functionalities involve the encapsulation of nanophosphors of controlled dimensions in a silica shell that enables an easy conjugation/bonding of the luminescent entities to biological systems. The SiO_2_ ceramic layer is an increasingly recurring component in biomedical applications and multifunctional nanomedicine due to attractive features such as good biocompatibility, controllable particle size and shape, and a particularly useful dual-functional surface area (exterior and interior).^36,37^ Tuning its pore size can lead to a convenient light transparency that enables the excitation and emission light to pass through, as necessary for bioimaging applications. Further modification of the silica surface would allow the host nanoparticles to reach the target, preventing their loss to nonspecific sites, and providing the overall system with increased functionality.^38,39^ The chemical functionalization of the shells with targeting groups relies on the ability to incorporate bio-orthogonal linkers having the ability to attach “addresses” which in turn will tackle a real need in the biological imaging space (with applications in ELISA-type diagnostics, for example). Over the past years, different research groups have been working on obtaining core–shell composite structures such as Er,Yb:NaYF_4_, Eu:Ga_2_O_3_, Eu:CaZr(PO_4_)_3_, or Eu:YVO_4_ nanoparticles wrapped with SiO_2_;^40−44^ but, despite some promising findings, the materials are not yet sold as fluorescent probes that can replace commercial organic dyes or quantum dots. Paramount complications related to the colloidal stability and degree of dispersion of the synthesized nanomaterials, in controlling the pore size distribution of the SiO_2_ coating, as well as in adjusting the nature of the inorganic–organic interfaces upon functionalizing the bioprobes (which itself constitutes a critical milestone that can spoil the luminescent response), are behind the adverse projection of these nanophosphor systems and may explain why they have not been widely accessed in the sensing and diagnostic bioimaging domain for cancer cells. Besides, the majority of the existing nanoscale syntheses routines are related to hands-on systems displaying one individual functionality or a single operational mode, whereas technological solutions involving complex nanostructures with multiple possibilities are far from being established.

This work reports on the rational design of a new family of synthetic platforms, composite structures based on luminescent nanophosphors and with a range of tunable properties so that they can replace common organic fluorophores [Alexa Fluor dyes, fluorescein, carboxyfluorescein diacetates (CFDA), carboxyfluorescein succinimidyl ester (SE), etc.]^45^ (Figure 1) as imaging agents for multiplexing bioimaging techniques. Using a soft-processing approach based on a hydrothermal synthesis and the Stöber process, hierarchical core–shell inorganic structures are obtained, functionalized with organic tags, and tested as promising candidates for luminescence imaging in cells. The synthetic protocols are systematically tailored aiming to understand the composition–function relationships, whereby the single excitation wavelength of light can create a number of emissions that can be measured simultaneously. For these multiplexing purposes, the nanophosphors are designed with high efficiency in the absorption and emission of photons (quantum yield), to obtain the highest possible signal intensity. This requires the imaging agents to be produced in a highly crystalline and suitably dispersed form, with the functionalized composites displaying an optimum size. As will be described, the elucidation of the luminescent properties of the nanocomposites in vitro, and their organelle colocalization, internalization, and biological stability in living cells will ultimately allow the development of a new generation of hybrid organic–inorganic biomarkers for cancer detection and monitoring in multiplex biosensing approaches.

## Results and Discussion

**Scheme 1:**
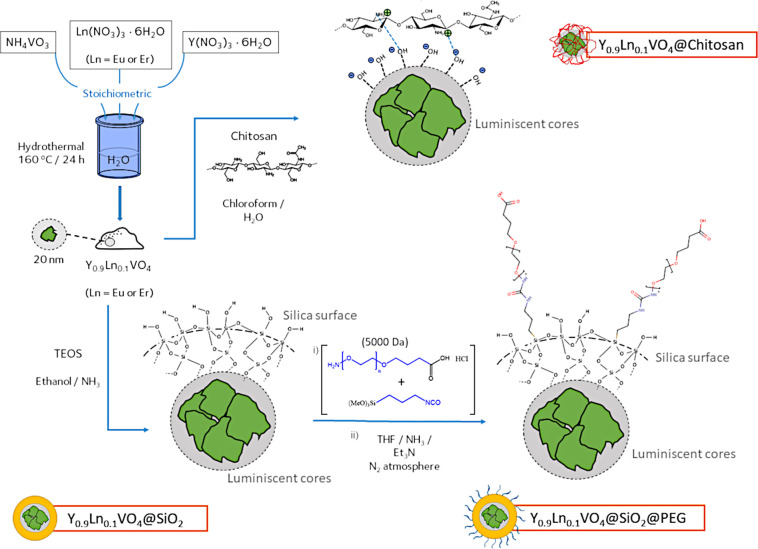
Designed, Synthesized, and Explored Composite Structures

To produce materials that exhibit superior performance over existing commercial products and to generate reliable material qualification and an appreciation of the in vitro behavior regarding interactions with cells, the following targets have been identified: (1) The ability to produce core particles smaller than 50 nm and composite bodies with an average radius below 150 nm; (2) To demonstrate the surface chemistry compatible with the linking of biologically active molecules and with the achievement of tunable dimensions; (3) To retain the brightness of the water-compatible nanophosphors which is currently known to be comparable to that of Quantum Dots; (4) To evaluate whether or not photobleaching in solution and in cells that inavertently affects their traceability on cells; (5) To evaluate the cellular morphology as a first indicator of the degree of toxicity in live cell imaging requirements. They have all been addressed by a synthetic and biophysical characterization involving the preparation of up to three composite structures of the luminescent cores, as summarized in Scheme 1.

### Design Features, Spectroscopy, and Structural Characterization of the Hydrothermally Produced Nanoceramic Cores

**Figure 2:**
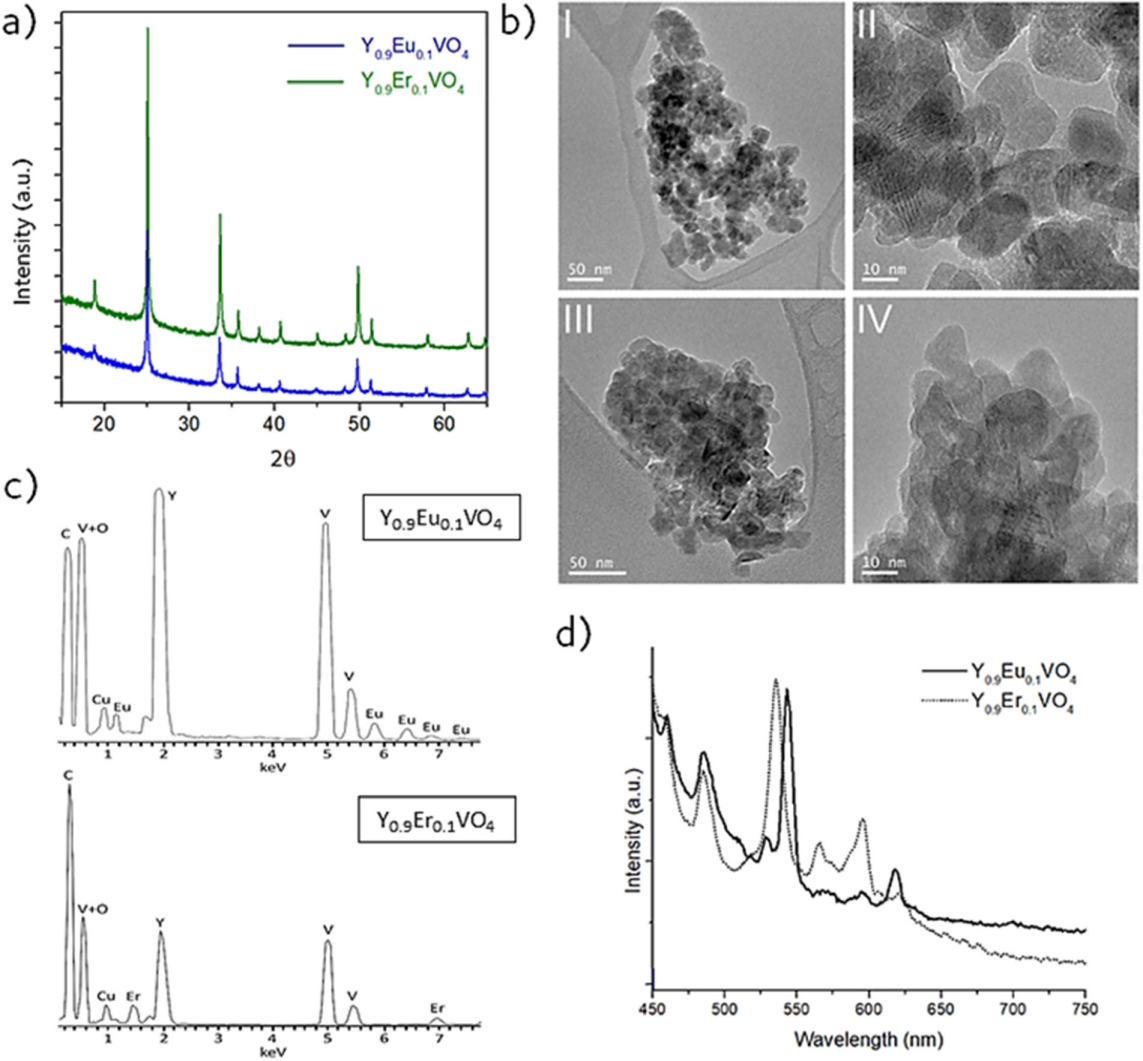
(a) X-ray diffractograms of Y_0.9_Eu_0.1_VO_4_ and Y_0.9_Er_0.1_VO_4_ nanoparticles, (b) TEM micrographs of Y_0.9_Eu_0.1_VO_4_ nanoparticles (I and II) and Y_0.9_Er_0.1_VO_4_ nanoparticles (III and IV), (c) EDS analyses of Y_0.9_Eu_0.1_VO_4_ nanoparticles and Y_0.9_Er_0.1_VO_4_ nanoparticles (the Cu signal comes to the copper TEM grid used), and (d) solid state fluorescence emission spectra of Y_0.9_Eu_0.1_VO_4_ (λ_exc_ = 398 nm) and Y_0.9_Er_0.1_VO_4_ nanoparticles (λ_exc_ = 392 nm).

With a view to the possible multiplexing applications of the conceived biomarkers, the high efficiency and narrow emission in the visible region of Ln-YVO_4_ nanophosphor formulations are based on their selection as the luminescent cores of the composites. Particularly, Europium and Erbium, two lanthanide elements with a well-known capacity as activator ions but with a slightly different excitation behavior, have been tested. The compositions were obtained using an optimized hydrothermal procedure^36^ which concedes high crystallinity and particle sizes well below (the targeted) 50 nm. Figure 2 summarizes the analytical characterization of the synthesized final probes. The recorded XRD patterns indicate that all reflections can be indexed to a tetragonal symmetry corresponding to a zircon-type structure (ICDD N. 17–0341), with no differences being observed upon changing the lanthanide ion, see Figure 2a. The representative TEM images in Figure 2b depict the morphological characteristics of the synthesized nanoparticles, whose specific composition was confirmed by EDS analyses (Figure 2c). The micrographs again indicate no significant differences when using one or another dopant. The high crystallinity already pointed by the XRD analyses is evidenced from the observation of some well resolved lattice fringes. In both cases, the average particle size is controlled around 20 nm. Further investigations on the particle size and its distribution were conducted by Dynamic Light Scattering (DLS) measurements, working with aqueous suspensions of 1 mg/mL concentration (Supporting Information, SI, Figure S1). The results obtained were not conclusive since in the absence of any type of surface functionalization, the small size of the nanoparticles pushes them to strongly agglomerate in solution. As will be discussed later, this natural tendency will not be a major impediment for the subsequent production of composites with a suitable degree of dispersion.

The optical performance of the synthesized cores was evaluated by fluorescence spectroscopy, allowing one to estimate their suitability for application as fluorophores in fluorescence microscopy (in vitro) and luminescent biomarkers (in vitro and in vivo studies).Figure 2d displays the solid state emission spectra of the Eu-doped and Er-doped compositions. In both cases, a common emission at ∼485 nm is first observed which is generally associated with the vanadate groups. The emission lines above 500 nm all correspond to the 4f-4f transitions of the Ln^3+^ ions, but the spectrum obtained differs when doped with Eu^3+^ or Er^3+^. In particular, there are variations in both the position and intensity of the recorded peaks, which are due to different electronic transitions of the Eu^3+^ and Er^3+^ centers. In the case of the Eu-doped cores, the specialized literature identifies the following transitions:^46−48^ 5D_1_ → 7F_1_ (centered at 530 nm in our sample), 5D_1_ → 7F_2_ (543 nm), 5D_0_ → 7F_1_ (double emission with peaks at 565 and 594 nm) and the 5D_0_ → 7F_2_ (618 nm); for the Er-doped materials, these other transitions have been reported: 2H_11/2_ → 4I_15/2_ (535 nm), 4S_3/2_ → 4I_15/2_ (560 and 590 nm), and 4F_9/2_ → 4I_15/2_ (622 nm). All these data are consistent with a partial replacement of the Y^3+^ ions in the YVO_4_ network by the respective Eu^3+^ or Er^3+^ dopants (in an environment with a D_2d_ symmetry),^47^ and further confirm the appropriateness of these hydrothermally produced samples to obtain a highly efficient luminescent response. These results have been compared with commercial samples of analogous composition, revealing however a dissimilar optical response that may be attributed to the synthesis method (Figure S2).

### Functionalization of As-Made Nanophosphors Cores Leading to Chitosan- and Silica-Coated Composites

A first generation of hybrid composites were obtained by encapsulating the synthesized nanophosphors encased with a chitosan layer (Scheme 1). A soft gelation methodology adapted from Hyeon et al.^49^ was used to generate the coating. This material was desirable to ensure biocompatibility, as chitosan is an extracellular, biocompatible, and biodegradable polysaccharide polymer that represents a very flexible formulating solution for a variety of applications, including controlled drug delivery, tissue-engineering, wound dressing material, biosensors, or membrane separators.^50^ Coating with chitosan will allow to evaluate the in vitro behavior and further viability of the inorganic cores inside the cells, but for practical (clinic) applications it may represent a too labile encapsulation approach thus leading to early breakup in vivo. Alternatively, the luminescent nuclei were encapsulated with amorphous SiO_2_ using the Stöber process.^51^ Coating with silica can provide a more effective shielding of the cores, further reducing nontarget cytotoxicity without initially affecting the optical performance of the assembly.^22^

**Figure 3:**
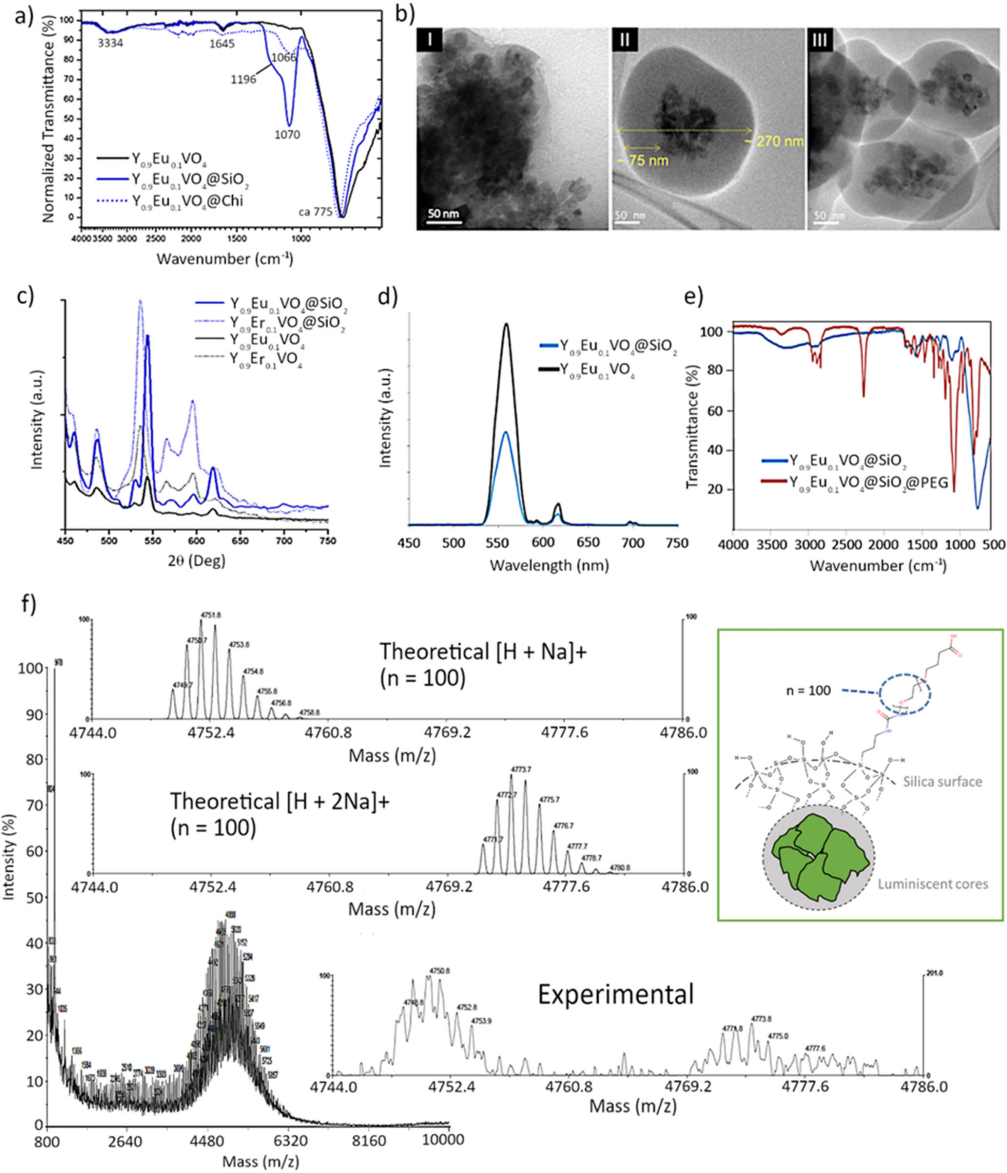
(a) FTIR of Y_0.9_Eu_0.1_VO_4_ nanoparticles, Y_0.9_Eu_0.1_VO_4_@SiO_2_, and Y_0.9_Eu_0.1_VO_4_@Chitosan composites. (b) TEM images of the (I) Y_0.9_Er_0.1_VO_4_@Chitosan, (II) Y_0.9_Er_0.1_VO_4_@SiO_2_ and (III) Y_0.9_Eu_0.1_VO_4_@SiO_2_ composite samples. (c) Solid state fluorescence emission spectra of Y_0.9_Eu_0.1_VO_4_@SiO_2_ nanoparticles (λ_exc_ = 398 nm) and Y_0.9_Er_0.1_VO_4_@SiO_2_ nanoparticles (λ_exc_ = 392 nm). (d) Fluorescence emission spectra of Y_0.9_Eu_0.1_VO_4_ and Y_0.9_Eu_0.1_VO_4_@SiO_2_ nanoparticles in water (0.05 mg/mL). (e) FTIR spectra of the free and functionalized Y_0.9_Ln_0.1_VO_4_@SiO_2_ composites. (f) Mass spectra of PEG precursor *M*_w_ ≈ 5000 Da. which was used to subsequently functionalize the surface of the final composites (inset experimental and calculated distribution of the PEG polymer corresponding to n = 100 units, *M*_w_ ≈ 5000 Da).

To evaluate the characteristics of the biopolymer coating and to assess whether confinement effects occur in the luminescent cores after their encapsulation, the FTIR characterization was carried out; the spectra in Figure 3a all belong to the Eu-doped materials, although completely analogous data were obtained for the compositions with Erbium. As can be seen, an intense absorption band centered around 775 cm^–1^ is resolved in all cases. This band is assigned to the antisymmetric stretching of the VO_4_^–^ vanadate group, and the fact that it registers equally in both the pristine nuclei and the composites suggests that no significant structural change occur in the vanadate matrix upon completion of the coating reactions (the minimal shift toward higher wave numbers in the composite materials is just attributed to an increased vibrational energy produced by the confinement of the nanoparticles inside the coatings). Apart from that, the additional bands that only show up in the spectra of the composite samples further evidence that the encapsulations have been effectively conducted. In the case of the chitosan-coated material, the band at 1066 cm^–1^ corresponds to the C–O–C groups characteristic of the polysaccharide molecule. For the SiO_2_ coated compositions, the absorption bands at 1072 and 1196 cm^–1^ are associated with the symmetric stretching of O–Si–O and Si–O–Si.

Figure 3a depicts the corresponding TEM images of the two series of composites (Figure S3 summarizes the compositional analyses as measured by EDS). On the one hand, the encapsulation with chitosan renders a compacted nanometric structure in which a relatively great number of nanoparticles are wrapped in a uniform layer of the polysaccharide. The polymeric nature of chitosan hampers the acquisition of steady images in the microscope, but a thickness about 10 nm could be estimated for the chitosan layer around the dense core of nanophosphors, with the composite units displaying an average size of 200 nm. The situation considerably changes for the SiO_2_-coated samples produced by the Stöber routine. An archetypal core–shell hierarchical structure is obtained consisting of a core of a few units of the luminescent Y_0.9_Ln_0.1_VO_4_ nuclei, homogeneously encapsulated in a SiO_2_ shell whose thickness (ca. 75 nm) can be easily controlled throughout the process. The statistical treatment of the micrographs reveals a mean size for the whole composite slightly larger than that of the chitosan-coated materials (250 nm on average), indicating that the aforementioned tendency of the synthesized nanoparticles to agglomerate is effectively prevented during the formation procedure of the composites. These data were corroborated by DLS investigations, taking the SiO_2_-coated composites as the representative samples (Figure S4). When working with water suspensions with a solid load of 1 mg/mL, a certain trend to agglomeration was again registered, bringing the existence of agglomerate units of around 800 nm in size. However, when the dispersions are diluted to solid loads of 0.1 mg/mL, the mean size measured by DLS reduces to 200 nm, i.e., nearly to the size of a single composite as identified by TEM. These results actually stress the importance of controlling the concentration of the suspensions that will be used later during the cell incubation stage.

In the case of the silica-coated materials, the formation of the inorganic SiO_2_ shell was further characterized by pore size distribution analyses and specific surface area measurements (Figure S5). These two microstructural parameters can critically impact the behavior and overall viability of these composites inside the cell environment. Specifically, a monomodal distribution of the pores with maxima at 15–20 nm was detected, while the N_2_ adsorption/desorption isotherm rendered a Brunauer–Emmet–Teller (BET) surface area of 7.6 m^2^·g^–1^. This last figure was complemented with the measurement of the Brunauer–Joyner–Halenda (BJH) area, which provides information on the existence of mesoporosity (sizes between 1.7 and 30 nm) and which returned a value of 1.2 m^2^·g^–1^. Eventually, the combination of all these data indicates that we are dealing with a relatively low surface area so that the existing pores are mainly distributed in the outer part of the SiO_2_ shell. This is a very promising configuration, as it will avoid the lixiviation of the composite as well as any possible leakage of the lanthanide ions in the cores to the cellular media.

Figure 3c shows the fluorescence spectra of the two series of composites. On the one hand, the chitosan-coated nanophosphors return the same spectra (position and intensity of the emission lines) as those obtained for the uncoated nuclei, confirming, as previously seen with similar inorganic materials,^52^ that the coating with this polysaccharide does not interfere with the luminescent emission of the cores. In contrast, the encapsulation within the silica shell gives rise to an interesting effect since, according to Figure 3c, an increase in the intensity of the fluorescent emission of the composites was observed. A similar phenomenon had already been reported^36^ and assigned to a process related to the effective suppression of the surface quenching effect of the luminescent nanocrystals: nanoparticles typically show a high density of surface defects. These defects can act as recombination channels of nonradiative centers for electrons and voids and this can cause a reduction in the quantum yield of the nanophosphors.^53^ Coating the constituent nanoparticles with a transparent, homogeneous shell can drastically reduce the density of surface defects (and even fully suppress them) and, consequently, the intensity of luminescence increases. Figure 3d finally depicts the optical response of the composites in an aqueous solution, specifically working with water suspensions of 0.05 mg/mL. As can be seen, the fluorescence of the suspended composites changes significantly with respect to that in the solid state. First, the signal corresponding to the vanadate groups is no longer detected, indicating that their emission is completely dispersed in the liquid medium. The nuclei on the other hand provide a widened contribution in solution, in an effect that is typically attributed to the interaction with the solvent.^54^ Even though the emission wavelength still remains in a narrow range, highlighting the excellent possibilities of these composites for multiplexing applications.

#### Further Functionalization of the SiO_2_-Coated Composites with PEG

As mentioned in the Introduction, several contributions have been published in recent years reporting on the improved possibilities of core–shell Ln:YVO_4_@SiO_2_ composite materials as fluorescent probes to replace commercial biomarkers. Despite the improvements, some fundamental challenges still hamper the deployment of these nanophosphor materials to the clinic. These include nonspecific binding to nontargeted or nondiseased areas, but also the uptake by the reticuloendothelial system (RES), where the nanomaterials are rapidly shuttled out of circulation to the liver, spleen, or bone marrow.^55^ Concerns about nanoparticles toxicity often arise because of this RES accumulation (aggregation may lead to entrapment in the liver, lungs, or elsewhere due to capillary occlusion), and a feasible approach to avoid this negative scenario involves modifying the surface of the nanoparticles, functionalizing them so that the RES uptake is significantly avoided and the circulation time is increased. Polyethylene glycol (PEG) is a potential candidate to meet these expectations. PEGylation of nanoparticles has been traditionally used in vitro to reduce nonspecific interactions with serum and nontargeted tissue proteins, which typically results in the so-called “stealth” behavior.^56−61^ The PEG chains reduce the charge-based contact typical of proteins as well as any other interaction with small molecules. Solubility in buffer and serum increases due to the hydrophilicity of the ethylene glycol arms and, also, the enhanced permeability and retention (EPR) effect is modulated due to changes in the size of the nanoparticles changes via addition of a PEG coat.^55^ Due to these attributes, PEGylated nanomaterials generally accumulate in the liver in half to one-third of non-PEGylated materials, also demonstrating a higher tumor accumulation versus background.^61,62^ In the Ln:YVO_4_@SiO_2_ samples, the silica coating is readily derivatized, eventually allowing for a convenient conjugation of the PEG molecules to the composite surface (Scheme 1). Specifically, the surface of the core–shell Y_0.9_Ln_0.1_VO_4_@SiO_2_ nanocomposites was functionalized using a PEGylated silane polymer that covalently anchors to the silica shell (Scheme 1). One-pot, stepwise functionalization reactions were initially performed with PEG linkers of different molecular weight, *M*_w_ = ca. 2000 g/mol, ca. 3000 g/mol, and ca. 5000 g/mol (see PEGylation Procedures in SI Reaction Approach 1). The hybrid materials functionalized with the lengthiest functional chains of PEG (with Mw ca. 5000 g/mol, optimized as described in SI Schemes S1–S2) have been assembled, as described in Figure 3, and characterized by mass spectrometry and data are depicted in Figure 3e together with its FTIR (Figure 3f), leading to the PEGylated functionalized Y_0.9_Ln_0.1_VO_4_@SiO_2_ composite. Iterative uptake experiments showed that a reliable in vitro uptake is preferentially attained when the surface of the nanoparticles is functionalized with the linker denoted PEG-5000 (see the SI).

The as-produced functionalization was evaluated by a systematic FTIR characterization that is summarized in Figure 3e for Y_0.9_Eu_0.1_VO_4_@SiO_2_ composite, as representative for both compositions since no substantial differences are observed. The recorded spectra show a number of bands at the lowest frequencies that are common to both composite materials, with and without PEG. On the one hand, the intense band in the range 750–800 cm^–1^ can be associated with the metal-O stretching vibrations of the Ln-doped YVO_4_ ceramic cores, while the band observed at 1095 cm^–1^ is indicative of a Si–O stretching and therefore comes from the SiO_2_ shell. At higher frequencies, a series of bands arise that are only detected in the functionalized material and which all correspond to different vibrations of the PEG molecule. Bands at 1648 cm^–1^, 1554 cm^–1^ (N–H bend region), and 1198 cm^–1^ (C–N stretch region) indicate the presence of a −NH_2_ group. The band at ca. 2300 cm^–1^ corresponds to the isocyanate group and, finally there is also a band at 2940 cm^–1^ that is indicative of the N–H stretch. Figure 3f shows the experimental isotopic distribution of the used PEG (5000 Da, *n* = 100) in the range of 800 to 10 000 *m*/*z*, as it can be observed that the theoretical isotope distributions are in agreement with the experimental ones, confirming the presence of the PEG-5000. These results indicate the successful functionalization of the core–shell units with the PEG molecules, with the consequent formation of a hierarchical Ln:YVO_4_@SiO_2_@PEG hybrid composite that, as will be discussed next, will continue to exhibit an excellent optical performance.

### Confocal Fluorescence Imaging for the Uptake of New Optical Probes in Living Cancer Cells

**Figure 4:**
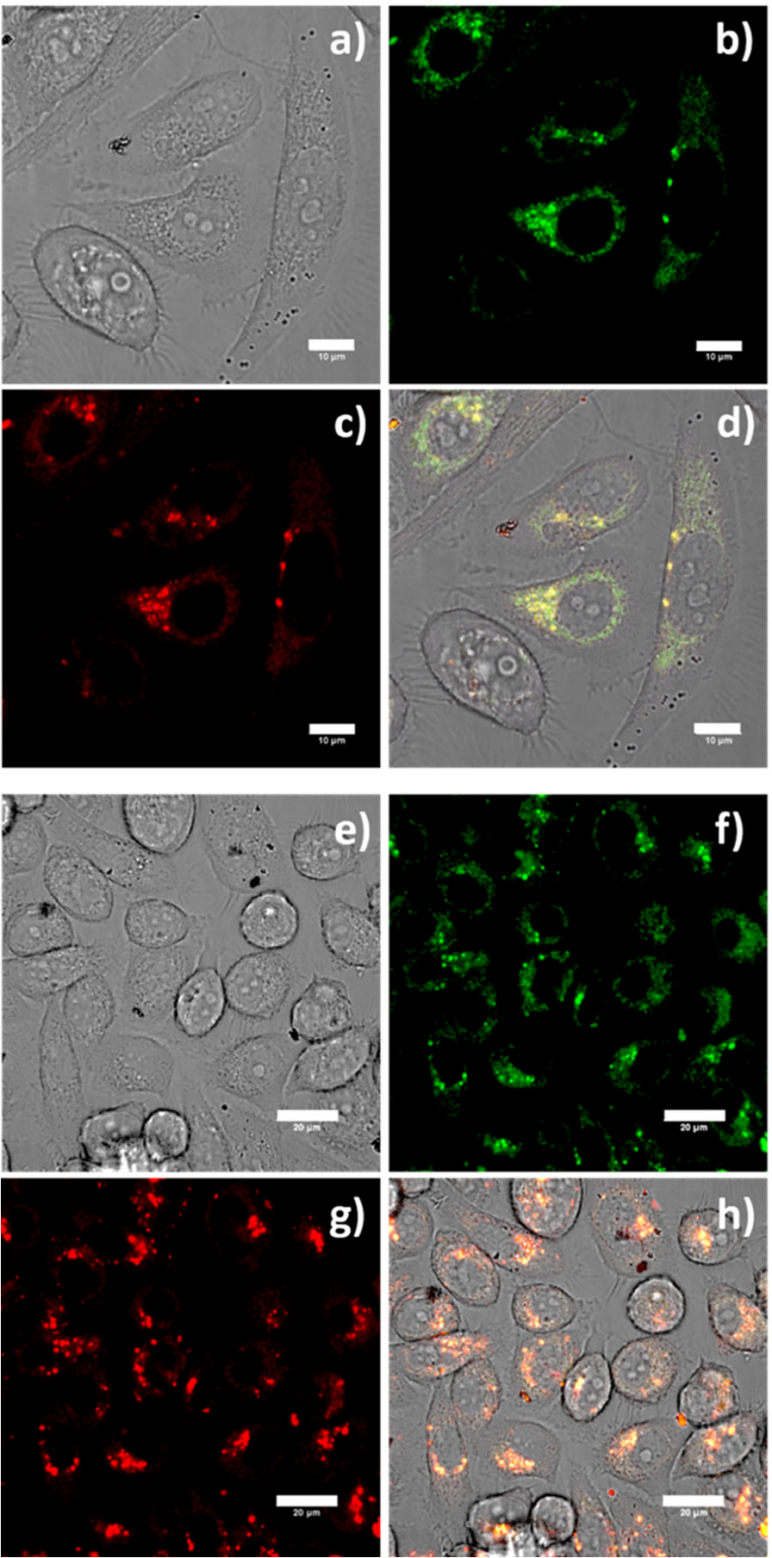
Confocal image of live PC-3 cells incubated with chitosan-wrapped composites at 1 mg/mL for 15 min: Y_0.9_Eu_0.1_VO_4_@Chitosan (a) DIC image; (b) green channel, λ_em_ = 516–530 nm; (c) red channel, λ_em_ 615–650 nm; and (d) overlay of the green-red channels; λ_ex_ = 488 nm. Scale bar: 10 μm; Y_0.9_Er_0.1_VO_4_@Chitosan (e) DIC image; (f) green channel; λ_em_ = 516–530 nm (g) red channel λ_em_ 615–650 nm; (h) and overlay of the green-red channels; λ_ex_ = 488 nm. Scale bar: 20 μm.

The newly synthesized materials were tested for their uptake in living cells. As-made pristine Y_0.9_Ln_0.1_VO_4_ cores are not suitable for biomedical applications and require derivatization to increase biocompatibility, isolate them from the media to avoid fluorescence quenching effects and facilitate their dispersion in water. This has been achieved by the formation of the above-described composites using an extracellular polysaccharide (chitosan) that favors water solubility, bicompatibility, and is biodegradable; SiO_2_, which improves dispersibility and derivatization, in addition to be biologically inert; and an additional functionalization with PEG of the SiO_2_-coated composites to passivate the surfaces and diminish the association with nontargeted serum and tissue proteins. In this context, single photon fluorescence confocal microscopy was used to image and examine the morphology and cellular biolocalization of the composite probes in vitro, and to establish their potential as fluorescent imaging agents for cell and tissue studies. For in vitro testing, prostate cancer cells (PC-3 line) were grown according to standard protocols, placed onto glass bottomed Petri dishes, and allowed to grow to suitable confluence (see Experimental Section for cell culture and plating details). Figure 4 shows the confocal images of PC-3 cells incubated with 1 mg/mL of Y_0.9_Eu_0.1_VO_4_@Chitosan and Y_0.9_Er_0.1_VO_4_@Chitosan. The results indicate an effective cell uptake for these composites, both profusely entering inside the cells and providing an intense fluorescence which is characteristic of a narrow emission (from the nanophosphor cores). The cellular biodistribution appears to be slightly different, for the case of Y_0.9_Eu_0.1_VO_4_@Chitosan vs Y_0.9_Er_0.1_VO_4_@Chitosan, with the Eu-doped nanophosphors appearing largely distributed through the cytoplasm and the Er-containing materials seem to accumulate more in lipid-rich regions. The morphology of cells after incorporating the fluorescent probes remains unaltered with respect to control experiments, with no apparent sign of membrane damage caused by the composites on the durations of experiments.

Several factors could explain the successful penetration of these particular compositions inside the cells. One is the presence of chitosan itself, since being a polysaccharide it esteems the cellular incorporation, but equally important is the reduced size of the composites.^63^ Particle sizes below 120 nm are typically observed for endocytic uptake,^64^ but it has been reported that particles as large as 500 nm can be readily internalized by cells.^65,66^ With an average size around 200 nm after coating the luminescent cores with the organic layer (Figure 3b), the chitosan-coated composites therefore have a propitious size which explicates the extraordinary bioimages monitored in Figure 4.

**Figure 5:**
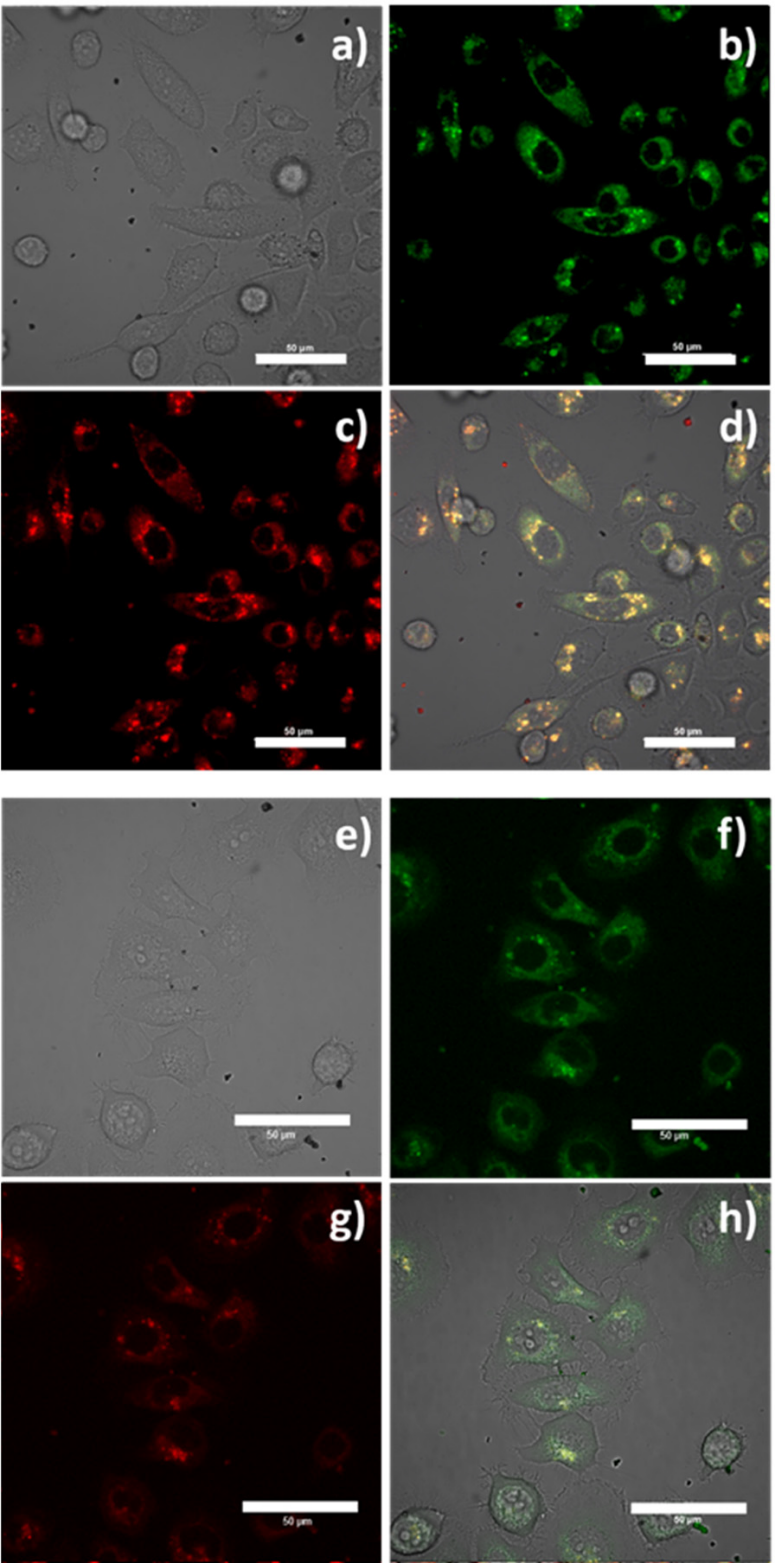
Confocal image of live PC-3 cells incubated with SiO_2_ based composites at 1 mg/mL for 15 min: Y_0.9_Eu_0.1_VO_4_@SiO_2_ (a) DIC image; (b) green channel, λ_em_ = 516–530 nm; (c) red channel, λ_em_ 615–650 nm; and (d) overlay of the green-red channels; λ_ex_ = 488 nm. Scale bar: 50 μm; Y_0.9_Er_0.1_VO_4_@SiO_2_ (e) DIC image; (f) green channel, λ_em_ = 516–530 nm; (g) red channel, λ_em_ 615–650 nm; and (h) overlay of the green-red channels; λ_ex_ = 488 nm. Scale bar: 50 μm.

However, as mentioned, the expediency of the chitosan coating would be limited in practical applications and the use instead of a SiO_2_ shell to shield the luminescent cores can be more operational. The cellular incorporation of these other composites was first studied on the core–shell units with no additional functionalization of the silica shell. Figure 5 shows that the cellular intake of Y_0.9_Eu_0.1_VO_4_@SiO_2_ and Y_0.9_Er_0.1_VO_4_@SiO_2_ compositions also takes place in an effective manner. The composites gave emission on both the green and red channels and most of the fluorescence intensity comes from the cytoplasm, where punctuated, highly localize characteristic to lysosomal incorporation has been observed, with no fluorescence being spotted from the cellular nucleus.

**Figure 6:**
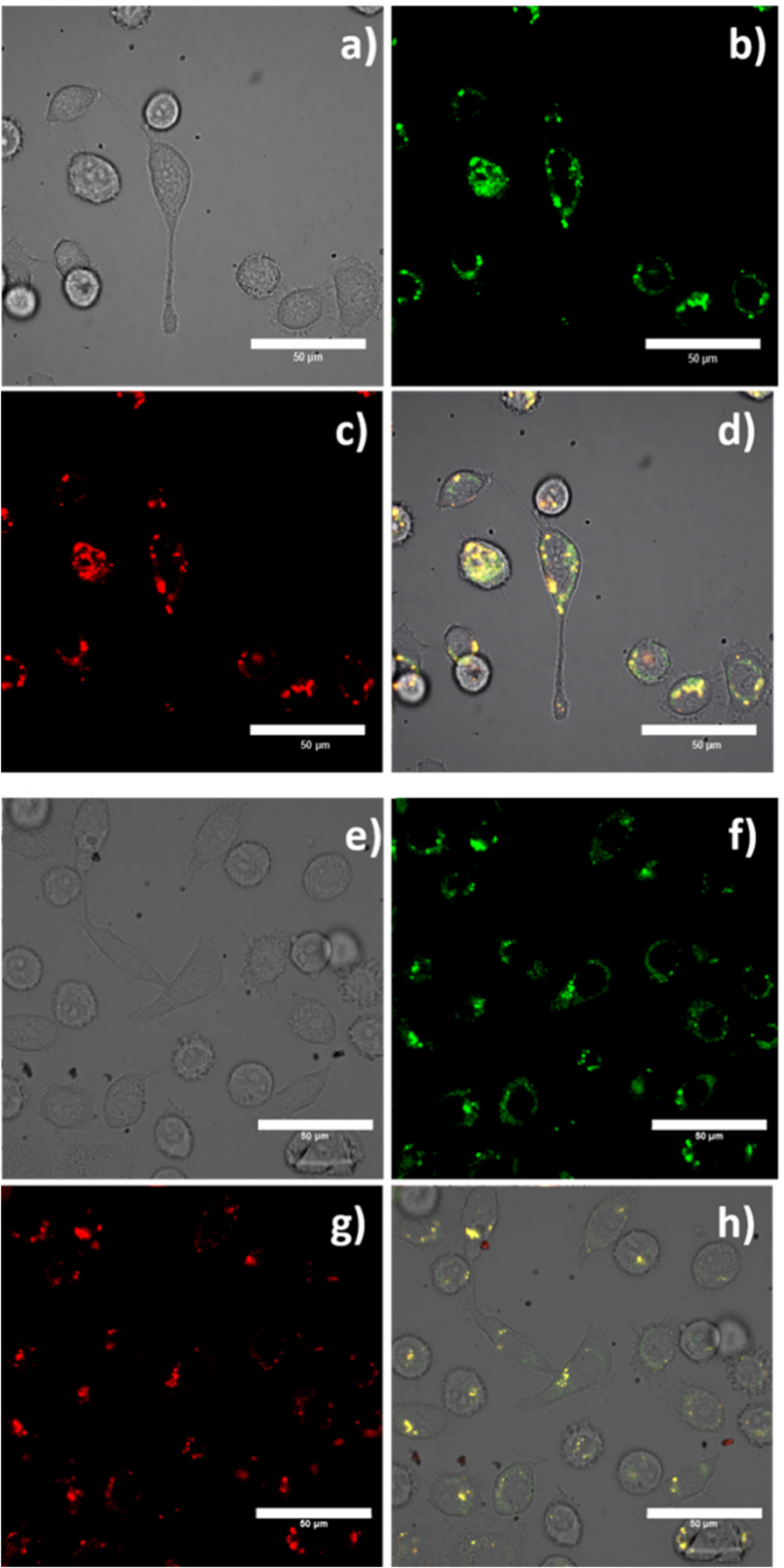
Confocal image of live PC-3 cells incubated with Y_0.9_Eu_0.1_VO_4_@SiO_2_ at 1 mg/mL for 15 min at 4 °C: (a and e) DIC image; (b and f) green channel, λ_em_ = 516–530 nm; (c and g) red channel, λ_em_ 615–650 nm; and (d and h) overlay of the green-red channels; λ_ex_ = 488 nm. Scale bar: 50 μm.

In the absence of the polysaccharide binding tag, the size of the composites (about 250 nm, Figure 3b) and their degree of dispersion now become decisive for an optimum cellular uptake: as the DLS measurements revealed no agglomeration occurs when these SiO_2_-coated structures are in suspension, eventually allowing a massive incorporation into the cells. Moreover, the low-temperature uptake at 4 °C was also tested for a representative sample of the Y_0.9_Eu_0.1_VO_4_@SiO_2_ composite. After 15 min of incubation, the confocal images depicted in Figure 6 reveal a bright red emission coming from within the cell. Again, limited changes in the morphology of the cells are produced and the cell membranes remain intact, but a lower incorporation of the composite is observed. To some extent, this indicates that the low temperature inhibits the active transport and slows the passive diffusion, which in other words implies that the cellular penetration of these composites likely occurs by both active and passive transport mechanisms, as verified by uptake at either 4 and 37 °C incubation experiments.^22^

Extensive confocal fluorescence microscopy characterization was carried out including: control PC-3 and HeLa cells and the incubations with Y_0.9_Eu_0.1_VO_4_@SiO_2_ and Y_0.9_Eu_0.1_VO_4_@SiO_2_ costaining with nuclear Hoechst dye (see SI). The cellular imaging experiments include 6-h as well as 24-h incubation of the silica-coated nanoparticles in PC-3, and analogous experiments with HeLa. The morphology of the cells remained stable, and there was no sign of damage caused by the uptake of this composite at 37 °C on the time scale of cellular imaging experiments (see SI). These results show that the Y_0.9_Eu_0.1_VO_4_@SiO_2_ compositions are biocompatible, a fact that is reinforced by the results of the MTT tests carried out, which show nearly negligible cytotoxicity with respect to control at 48 h (see SI). This is in line with similar works reported on SiO_2_-encapsulated ceramic nanoparticles.^22,67^

**Figure 7:**
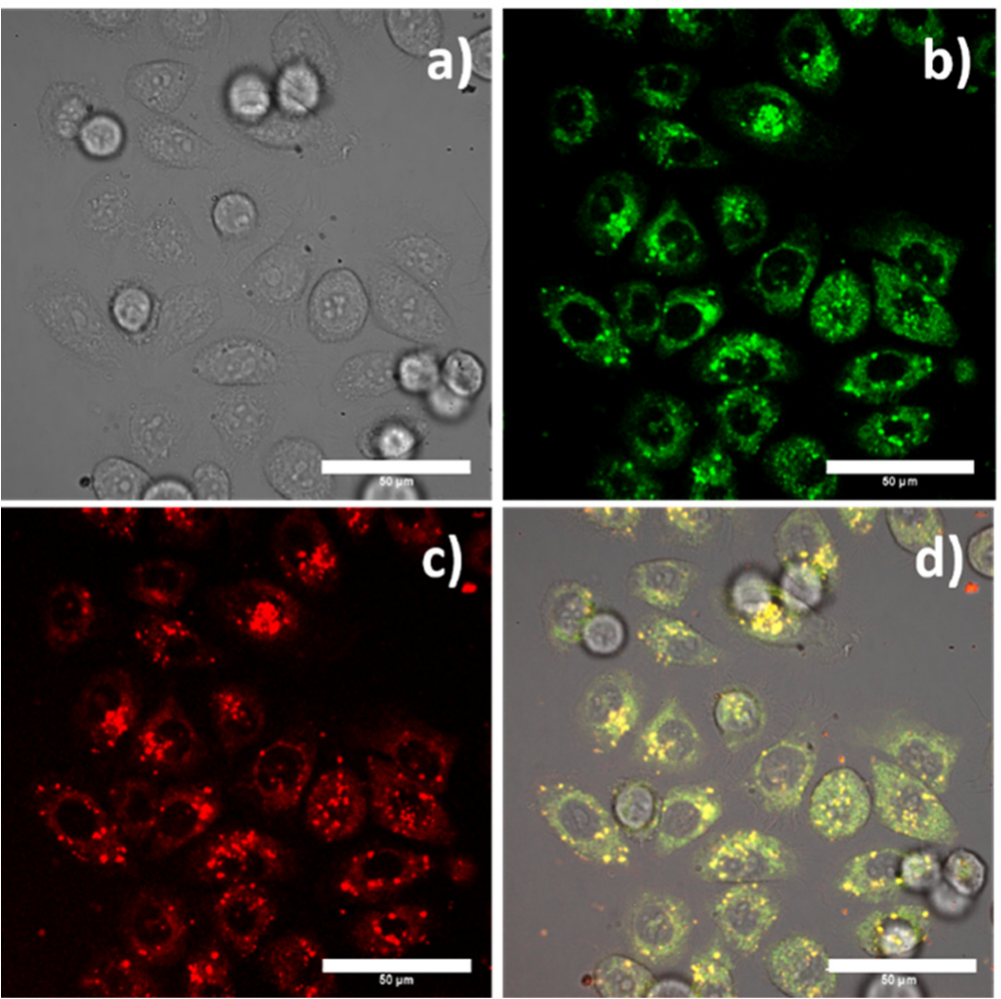
Confocal image of live PC-3 cells incubated with Y_0.9_Eu_0.1_VO_4_@SiO_2_@PEG (where the PEG linker was the conjugated PEG-5000 variant) at 1 mg/mL for 15 min at 4 °C: (a) DIC image; (b) green channel, λ_em_ = 516–530 nm; (c) red channel, λ_em_ 615–650 nm; and (d) overlay of the green-red channels; λ_ex_ = 488 nm. Scale bar: 50 μm.

Finally, the uptake of the SiO_2_-coated PEGylated composites was also analyzed in the confocal microscope. Figure 7 shows the corresponding images for the Y_0.9_Eu_0.1_VO_4_@SiO_2_@PEG hybrid heterostructure, but it can be taken as representative since no substantial differences were now obtained upon dealing with the Eu-doped or Er-doped formulated nanophosphors. The registered images again show no clear damage to the cells and a fluorescence emission in the red and green channels that, as happened with the nonfunctionalized SiO_2_-coated composites, mainly comes from inside the cell. There is, however, a big difference compared with the previous materials: the intensity of the fluorescence is much higher and it comes from within the cell, indicating a greater penetration and more homogeneous distribution of these hybrid composites inside the cells. This can be better seen on the overlay of the green-red channels depicted in Figure 7d, and actually responds to the mentioned surfactant effect of the PEG chains (i.e., reduction of the charge-based contacts), which allow an improved accumulation of the luminescent probes in organelles such as mitochondria or lysosomes. To the best of our knowledge, such results of cell incorporation and viability have not been achieved before for similar biomarkers based on inorganic nanoparticles. They further confirm that the physical-chemical properties achieved with these hierarchical structures are adequate to protect the luminescent nanophosphors and the guest molecules from detection and degradation by the immune system, simultaneously maintaining the necessary dose of the nanoprobe to get an image in vitro at very low levels. These factors, additionally to the stepwise, very simple, and implicitly versatile synthetic routine, opens the door to large-scale production of luminescent nanoceramics and biocompatible hybrid materials with characteristic narrow emission bands and hence potential application in multiplex bioimaging.

## Conclusions

Functionalized composites of lanthanide-doped nanoparticles and SiO_2_ have been synthesized and studied as an alternative to organic optical bioimaging probes. The suitability of these materials as optical bioprobes have been tested by incubation in PC-3 cancer cells and subsequent studies by laser scanning confocal fluorescence microscopy. The incorporation of the luminescent nanoparticles within a SiO_2_ shell and their surface functionalization do not reduce the luminescent response while increasing the possible multifunctional applications of the materials. The cellular uptake mechanism and the biodistribution inside the cells of the different compounds were also evaluated. The functionalized composites show a good level of dispersion and biocompatibility and lead to promising potential application for cell imaging assays in addition to their usefulness in multiplexing assays as alternatives to the ubiquitous organic dyes. Due to their functional versatility and low toxicity, these new nanophorescent nanoparticles are potential candidates as synthetic platforms for multimodality imaging techniques with applicability in vitro and in vivo, such as optical imaging combined with PAI and/or optical imaging combined with nuclear imaging techniques such as Positron Emission Tomography (PET) or Single Photon Computed Tomography (SPECT).

## Experimental Section

### Materials and Methods

Reagents were obtained from standard commercial sources and were used as received. *Mass spectrometry* data was acquired, in low resolution, on a Bruker Daltonics ESI-TOF, further MS analysis, in high resolution MALDI-TOF spectrometry, were carried out using the EPSRC National Mass Spectrometry Facility, Swansea, UK. *NMR* spectra were acquired on a Bruker 500 Ultrashield (^1^H: 500 MHz, ^13^C: 125 MHz). Mass spectrometry was carried out in HPLC-grade methanol or distilled water. NMR spectroscopy solvents were obtained commercially and used as received. *HPLC* was carried out using a Symmetry C-18 column (4.6 × 260 mm^2^) with UV–vis detection measured at eight wavelengths from λ_obs_ = 200, 220, 280, 300, 400, 450, 500, 600, 700, and 800 nm. The gradient elution was 0.8 mL/min, with 0.1% TFA Milli-Q water as solvent A and 0.1% TFA MeCN as solvent B. Start 95% A reverse gradient until 5% A at 7.5 min, isocratic until 15 min, reverse gradient until 17.5 min 95% A, then hold to 18 min. *UV–visible spectra* were obtained using a PerkinElmer Spectrometer, Lamda 650 in a 1.00 cm path length quartz cuvette. *Luminescence spectra* were measured in a 1.00 cm quartz cuvette using a PerkinElmer LS55 luminescence spectrophotometer. *Gel Permeation Chromatography* were performed at 2 mg/mL on a Polymer Laboratories PL-GPC 50 integrated system, with *M*_r_ Thomas Forder, using a PLgel 5 μm MIXED-D 300 × 7.5 mm^2^ column at 35 °C, THF solvent (flow rate, 1.0 mL/min). The polymers were referenced to 11 narrow molecular weight polystyrene standards with a range of *M*_w_ 615–5 680 000 Da. *Thermogravimetric analysis* was carried out on a TGA 4000 Thermogravimetric Analyzer (PerkinElmer). *Dynamic Light Scattering* were performed on a Nano-S Zetasizer (Malvern). *Infrared spectroscopy* was carried out on a Spectrum 100, FT-IR Spectrometer (PerkinElmer).

### Synthesis of Ln-YVO_4_ Nanoparticles

The Y_0.9_Ln_0.1_VO_4_ (with Ln = Eu or Er) nanoparticles were synthesized by a hydrothermal method under the optimized conditions as described elsewhere.^68^ Namely, the stoichiometric amounts of Y(NO_3_)_3_·6H_2_O, Ln(NO_3_)_3_·6H_2_O (Ln = Eu or Er) (Strem Chemicals) and NH_4_VO_3_ (Aldrich, 99%) were dissolved in 30 mL of distilled H_2_O and then poured into a Teflon-lined stainless steel autoclave. The reaction was carried out at 160 °C during 24 h under autogenous pressure. The obtained precipitate was washed several times with distilled water and then dried at 100 °C.

### Preparation of the Ln-YVO_4_@SiO_2_ Composites

The coating process was carried out by the Stöber process using tetraorthosilicate (TEOS) as the SiO_2_ source. Particularly, the previously synthesized NPs were dispersed in ultrasounds during 10 min in 50 mL of distilled water, 150 mL of ethanol and 5 or ml of NH_3_. Then, 1 mL of TEOS was added dropwise under continuous magnetic stirring which was maintained during 6 h until the reaction was completed. The reaction conditions were modified by varying the NH_3_ and the TEOS concentration to tailor the size and morphology of the obtained composites.

### Preparation of the Ln-YVO_4_@Chitosan Composites

For synthesis of chitosan composites, the method previously reported by Huawey was followed: 0.2 mL of luminescent nanoparticles dispersed in chloroform (1.13 mg Fe/mL) were added dropwise to a stirred solution of deionized water (3 mL, pH 7) containing 5 mg of chitosan-DOPA. The solution was sonicated for 3 min using a sonifier working at 20 kHz. The resulting oil-in-water emulsion was subjected to rotary evaporation at 40 °C for 5 min to remove residual solvent under reduced pressure. Excess polymer was removed by dialysis against deionized water for 1 day (Mw cutoff of 12 kDa).

### Surface Functionalization with PEG

The functionalization with PEG linkers was first carried out on a gram scale using bulk (powder) materials in a dispersed phase, which was optimised (as described in the SI) and then applied to the NP particles Y_0.9_Eu_0.1_VO_4_@SiO_2_. In typical experiments, a Schlenk flask was presilylated using dimethyldichloride silane in vacuo using a desiccator for 2 h and was then moved to an oven for 2 h at 200 °C. Ten mL dry, THF and 10 μL aqueous ammonia (34%) was added followed by 100 mg of the Y_0.9_Ln_0.1_VO_4_ nanoparticles under a flow of nitrogen and stirred for 1h. One mL Trimethoxy(3-isocyanotopropyl)silane was added and the reaction mixture was stirred overnight at room temperature. 100 mg of the 5000 g/mol aminooxy-PEG-carboxylic acid (see SI, scheme S1 and S2, for chemical formula) from NanoCS, denoted NH_2_–PEG-COOH·HCl, was added followed by 1 equiv of triethylamine under a flow of nitrogen. This was allowed to react for 5 h and then was centrifuged at 3000 rpm for 5 min and subsequently washed 3× with dry THF. More complete data on this process can be found in the SI.

### Characterization of the Synthesized Nanoparticles and Composites

Y_0.9_Eu_0.1_VO_4_ and Y_0.9_Er_0.1_VO_4_ nanoparticles were characterized through X-ray powder diffraction (XRD) on a Bruker D8 Advance diffractometer using Cu K_α1_ radiation (λ = 1.5406 Å). Step-scanned diffraction patterns were collected between 15 and 90° in steps of 0.02° and with a counting time of 1.5s per step; samples were prepared by placing a drop of a concentrated ethanol dispersion of particles onto a single crystal silicon plate. Transmission electron micrographs were obtained using a field-emission electron-source high-resolution transmission electron microscope (HRTEM) JEOL 2100F operated at 200 kV. The microscope was equipped with an energy-dispersive X-ray spectroscopy (EDS) system EDXS-ISIS300 from Oxford Instruments; samples were prepared by placing a drop of a dilute ethanol dispersion of nanoparticles onto a 300 mesh carbon-coated copper grid and evaporating immediately at 60 °C. The EDS spectra were quantified using Oxford ISIS software containing a library of virtual standards. The infrared spectra of the samples were obtained on a Fourier transform infrared (FTIR) spectrometer PerkinElmer Spectrum 100 equipment using the attenuated total reflectance (ATR) method. Spectra were recorded from 650 to 4000 cm^–1^, and 64 or 256 scans were averaged at a resolution of 6 or 4 cm^–1^. Specific surface area was determined by the BET method in a Monosorb Analyzer MS-13 QuantaChrome (U.S.A.). Nitrogen adsorption/desorption isotherms were carried out on an ASAP 2020-Micromeritics at 77 K. Samples were degassed at 150 °C during 48 h before analysis. The photoluminescence measurements of the samples were made at room temperature, using two measurement systems: a Horiba Jovin-Ybón LabRAM HR800 and a pulsed laser. In the former, the samples are excited with a wavelength of 325 nm from an Olympus BX 41 He–Cd laser, which is coupled to a confocal microscope, whose objective lens is 40×. A coupling detector that collects the scattered light and incorporates a 600 mm^–1^ line grid was used to collect the data. The spectral resolution of the system used was 0.1 nm. In the pulsed laser of approximately 50 fs duration, the measurements were made by exciting the samples at a wavelength of 333 nm, produced by a parametric optical amplifier (OPA). This system is coupled to a microluminescence system that allows the radiation emitted by the sample, after being excited, to be collected by Ocean Optics USB2000+ and BWTEK BTC261E fiber optic spectrometers.

### Incubation in PC3 Cells

Prostate cancer cells, PC-3 line, were originated from American Type Culture Collection (ATCC) and grown according to standard serial passage protocols.^69^ Cells were cultured at 37 °C in a humidified atmosphere in air and harvested once >70% confluence had been reached. PC3 cells were cultured in RPMI (Roswell Park Memorial Institute) 1640 medium. The media contained 10% fetal calf serum (FCS), 0.5% penicillin/streptomycin (10 000 IU mL-1/10 000 mg mL-1) and 1% 200 mM l-Glutamine. All steps were performed in absence of phenol red.

Supernatant containing dead cell matter and excess protein was aspirated. The live adherent cells were then washed with 10 mL of phosphate buffer saline (PBS) solution twice to remove any remaining media containing FCS, which may inactivate trypsin. Cells were incubated in 3 mL of trypsin solution (0.25% trypsin) for 5 to 7 min at 37 °C. After trypsinization, 6 mL of medium containing 10% serum was added to inactivate the trypsin and the solution was centrifuged for 5 min (1000 rpm, 25 °C) to precipitate cells. The supernatant liquid was aspirated and 5 mL of cell medium (10% FCS) was added to cells remain. Cells were counted using a hemocytometer and then seeded as appropriate on Glass bottomed Petri dishes (MaTek, 35 mm diameter and 1.5 mm thickness) at 1.5 × 10^5^ to 2.5 × 10^5^ cells per dish, allowed to grow during 24 h up to a suitable confluence. Laser confocal fluorescence imaging was then performed using our assays validated in previous experiments.^22^ Confocal microscopy images were acquired in a Nikon Eclipse Ti instrument equipped with 405, 488, and 561 nm excitations lasers. The obtained images were processed using the Nikon Elements-AR Analysis 4.30.02 software.^70^

## Supporting Information Available

DLS, TEM, EDS, and Fluorescence spectroscopy characterization; N_2_ adsorption/desorption studies; control and characterization of Y_0.9_Eu_0.1_VO_4_ commercial nanoparticles (quantum yield, stability of the luminescence with time, fluorescence microscopy, cell studies); PEGylation procedures (reaction approaches, thermogravimetric analysis, stability of the luminescence with time in aqueous media, TGA analyses, NMR spectra, TLC plates, GPC, Mass spectrometry); and confocal fluorescence imaging microscopy of Y_0.9_Eu_0.1_VO_4_ based composites (PDF)

## Abbreviations Used

PEG5000: Polyethylenglycol 5000 Da
QDs: Quantum Dots
NP: Nanoparticles
DLS: Dynamic Light Scattering
FTIR: Fourier Transform Infrared Spectroscopy
TEM: Transmission Electronic Microscopy
EDS: Energy-dispersive X-ray spectroscopy
PC-3: Prostate Cancer Cell Line
