# Supplementary material for: Hybrid Hierarchical Heterostructures of Nanoceramic Phosphors as Imaging Agents for Multiplexing and Living Cancer Cells Translocation
Source: ACS Appl Bio Mater. 2021 Mar 10;4(5):4105–18. doi: 10.1021/acsabm.0c01417 (PMC8155200; doi:10.1021/acsabm.0c01417)
Supplement: Supplementary file 1 — mt0c01417_si_001.pdf [file mt0c01417_si_001.pdf]

Supporting Information

Hybrid hierarchical heterostructures of nanoceramic phosphors as imaging agents for multiplexing and living cancer cells translocation

David G. Calatayud*, Teresa Jardiel*, Mara S. Bernardo, Vincenzo Mirabello, Haobo Ge, Rory L. Arrowsmith, Fernando Cortezon-Tamarit, Lorena Alcaraz, Josefa Isasi, Pablo Arévalo, Amador C. Caballero, Sofia I. Pascu* and Marco Peiteado*

Corresponding Authors
* David G. Calatayud – Instituto de Cerámica y Vidrio, Keslen 5, Campus de Cantoblanco, 28049, Madrid, Spain.
orcid.org/0000-0003-2633-2989
E-mail: dgcalatayud@icv.csic.es

* Teresa Jardiel – Instituto de Cerámica y Vidrio, Keslen 5, Campus de Cantoblanco, 28049, Madrid, Spain.
orcid.org/0000-0002-0163-7324
E-mail: jardiel@icv.csic.es

* Sofia I. Pascu – Department of Chemistry, University of Bath, Claverton Down, BA2 7AY, Bath, UK.
orcid.org/0000-0001-6385-4650
E-mail: s.pascu@bath.ac.uk

* Marco Peiteado – Instituto de Cerámica y Vidrio, Keslen 5, Campus de Cantoblanco, 28049, Madrid, Spain.
orcid.org/0000-0003-3510-6676
E-mail: mpeiteado@icv.csic.es

Table of Contents

DLS, TEM, EDS and Fluorescence spectroscopy characterization 2
N_2 adsorption/desorption studies 4
Control and characterisation of bulk powder particulate $Y_{0.9}Eu_{0.1}VO_4$ 4
Quantum yield 5
Stability of the luminescence with time 6
Fluorescence microscopy 7
Cell studies 8
PEG-ylation Procedures 11
Reaction approach 1 12
Approach 2: Test reaction approach for alternative PEG linkers functionalisation 16
Alternative functionalisation of linkers - reaction approach 3 17
Alternative reaction approach 4 18
Alternative reaction - approach 5 18
Reactions using protocols involving a pre-reacting to silane step 19
Thermogravimetric analysis 22
Probing the stability of the luminescence with time in aqueous media 23
TGA analyses 25
NMR spectroscopy for model linkers studies 26
Raman Spectroscopy: 27
GPC 28
Mass Spectrometry 31
Confocal fluorescence imaging microscopy of $Y_{0.9}Er_{0.1}VO_4$ and corresponding functional composites 42
Cellular Viability Tests 55
References 57

DLS, TEM, EDS and Fluorescence spectroscopy characterization

The degree of agglomeration of the nanoparticles in solution was followed by dynamic light scattering (DLS) measurements working with aqueous suspensions of 1 mg/ml in concentration. The results reveal the formation of agglomerates of around 1 μm in size (Figure S1), which would point to a strong trend of the synthesized nanos towards agglomeration; as explained in the text, this preliminary parameter will not be a major impediment to the subsequent production of composites with a suitable degree with which to achieve an unprecedented incorporation of the manufactured nanoparticles into the cells.

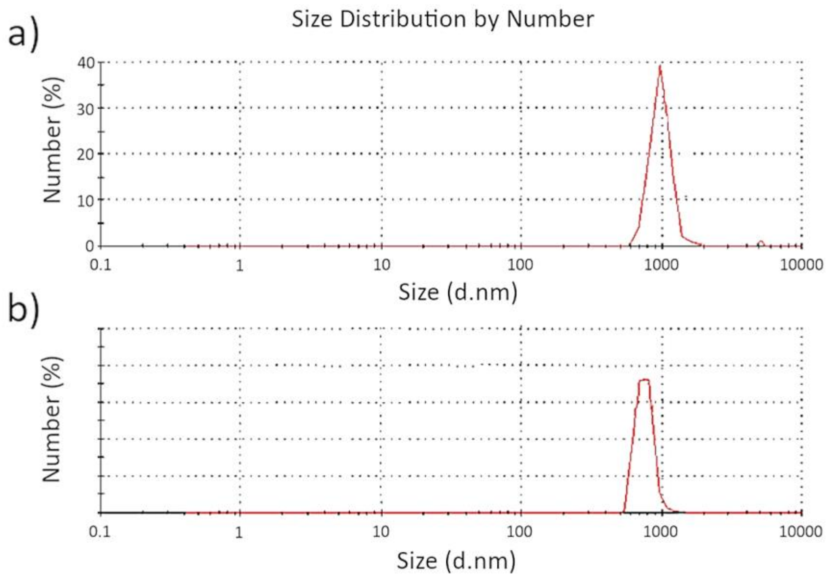

Figure S1. Dynamic light scattering spectra in water of a) $Y_{0.9}Er_{0.1}VO_4$ (concentration = 1 mg/ml in water) and b) $Y_{0.9}Eu_{0.1}VO_4$ (concentration = 1 mg/ml in water).

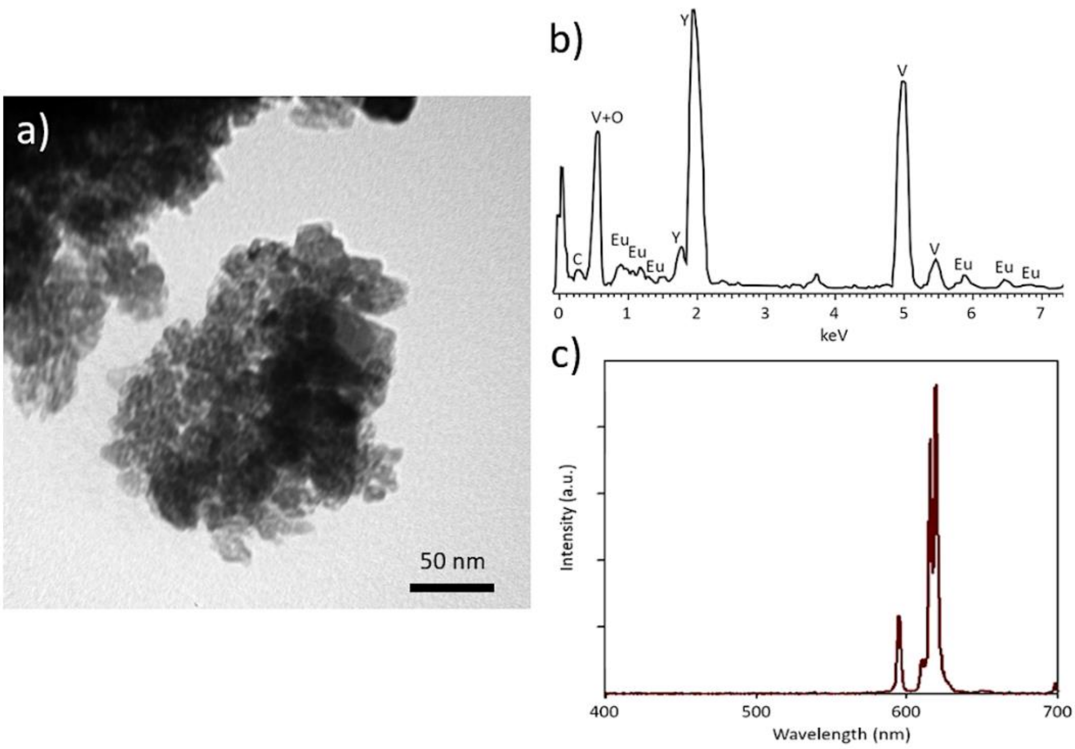

Figure S2. a) TEM micrographs, b) EDS analyses and c) solid state fluorescence emission spectra of bulk powder particulate $Y_{0.9}Eu_{0.1}VO_4$ (λ_{exc} = 350 nm).

Figure S3 show the EDS of @SiO_2 composites (those for Chitosan are alike, barely showing an increased intensity in the C and O signals). The EDX analyses in both samples indicate a homogeneous composition in agreement with the nominal $Y_{0.9}Eu_{0.1}VO_4$ and $Y_{0.9}Er_{0.1}VO_4$ formulas and the published data on commercial powder Eu:YVO_4 samples. The results indicate that the lanthanide dopants, either Er or Eu, are effectively incorporated into the vanadate matrix by this novel hydrothermal method and are maintained after the processes of obtaining the core-shell composites.

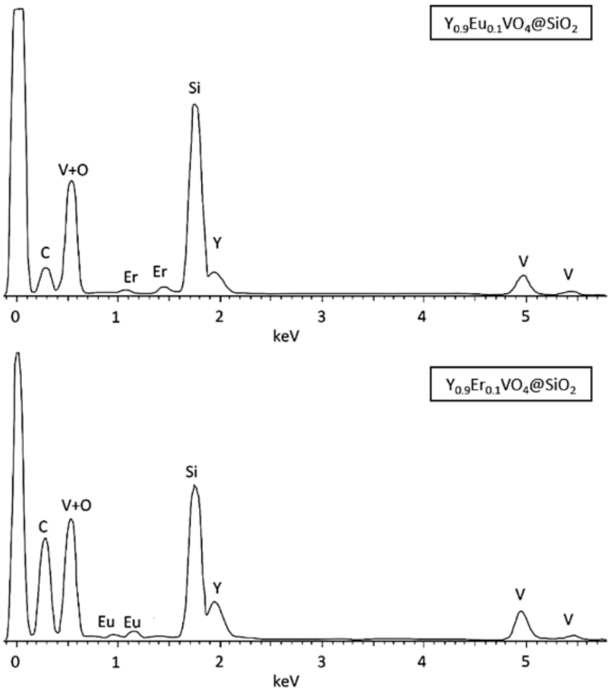

Figure S3. EDS analyses of $Y_{0.9}Eu_{0.1}VO_4$@SiO_2 and $Y_{0.9}Er_{0.1}VO_4$@SiO_2 composites.

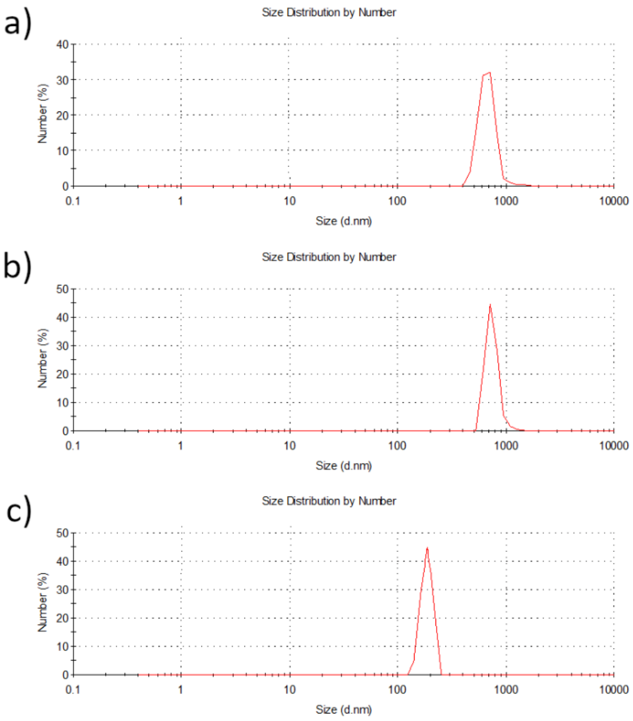

Figure S4. Dynamic light scattering spectra of a) 1 mg/ml of $Y_{0.9}Er_{0.1}VO_4$@SiO_2 composite in water, b) 1 mg/ml of $Y_{0.9}Eu_{0.1}VO_4$@SiO_2 composite in water and c) 0.1 mg/ml $Y_{0.9}Eu_{0.1}VO_4$@SiO_2 composite in water.

N_2 adsorption/desorption studies

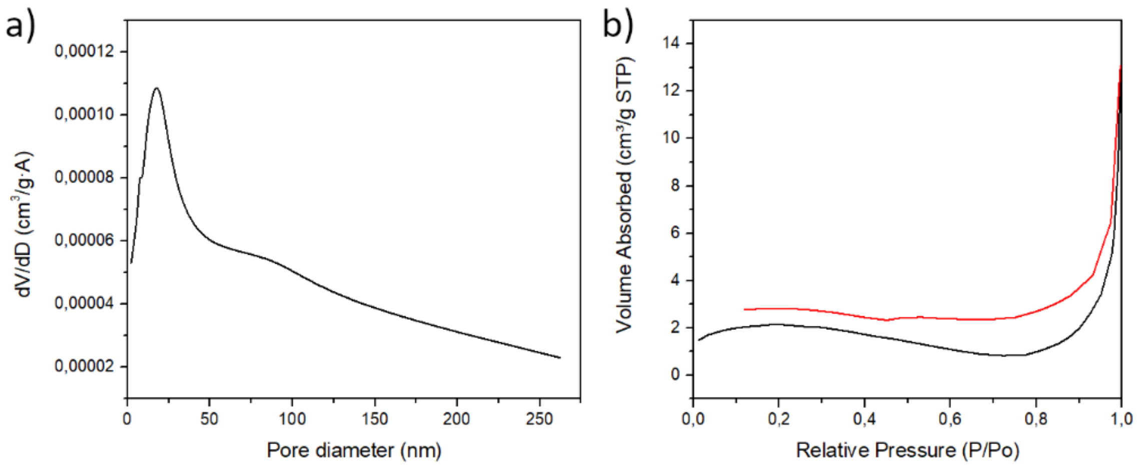

Figure S5. a) Pore size distribution curves and b) nitrogen adsorption-desorption BET isotherms of $Y_{0.9}Er_{0.1}VO_4$.

Control and characterisation of bulk powder particulate $Y_{0.9}Eu_{0.1}VO_4$

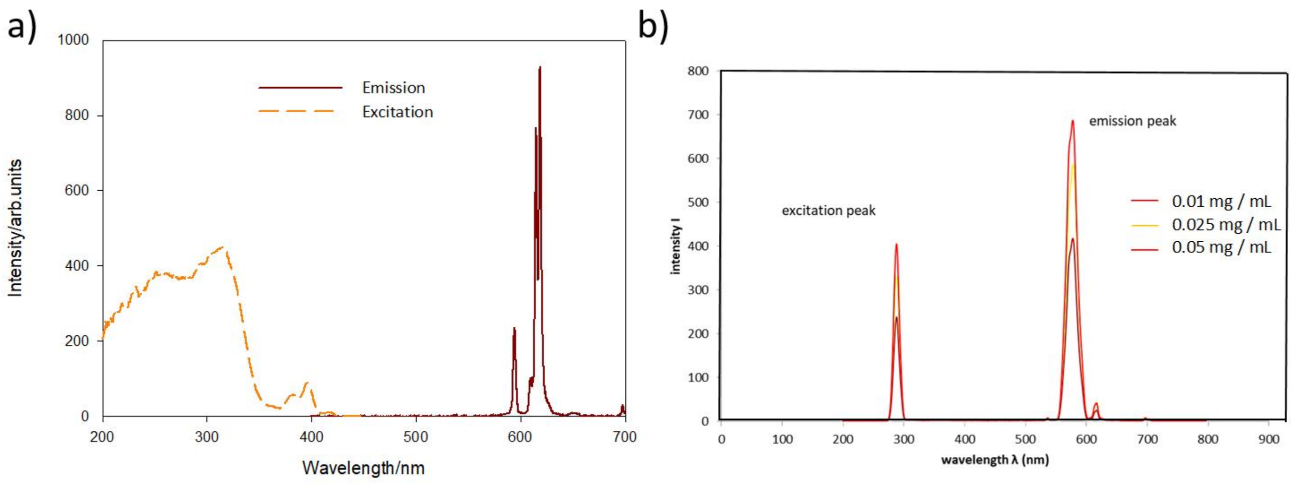

Figure S6. a) Solid state fluorescence excitation and emission spectra of bulk powder particulate $Y_{0.9}Eu_{0.1}VO_4$ (λ_{exc} = 350 nm) and b) fluorescence excitation and emission spectra bulk powder particulate $Y_{0.9}Eu_{0.1}VO_4$ in water at different concentrations (λ_{exc} = 280 nm).

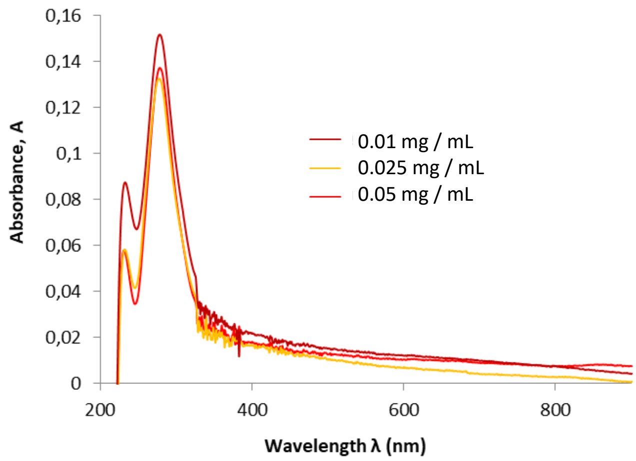

Figure S7. UV-vis spectra of bulk powder particulate $Y_{0.9}Eu_{0.1}VO_4$ in water at different concentrations.

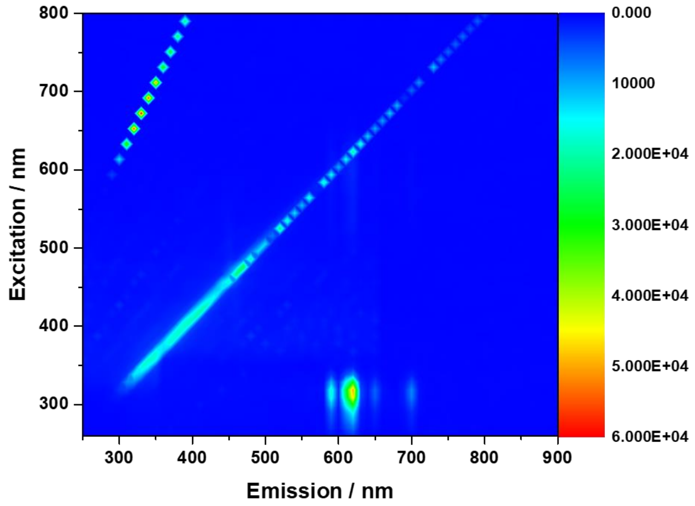

Figure S8. Normalized 2D fluorescence contour plots recorded for dispersions in water (0.05 mg/mL) of bulk powder particulate $Y_{0.9}Eu_{0.1}VO_4$.

Quantum yield

Figure S6 shows the excitation / emission spectra of bulk powder particulate $Y_{0.9}Eu_{0.1}VO_4$ with different concentrations in water. As it can be seen on the spectra samples absorbed at 280 nm. So, the calculated quantum yields were obtained at 280 nm using tryptophan which absorbs at 280 and 290 nm as reference. Figure S9 shows the fluorescence emission spectra of tryptophan and fluorescein.

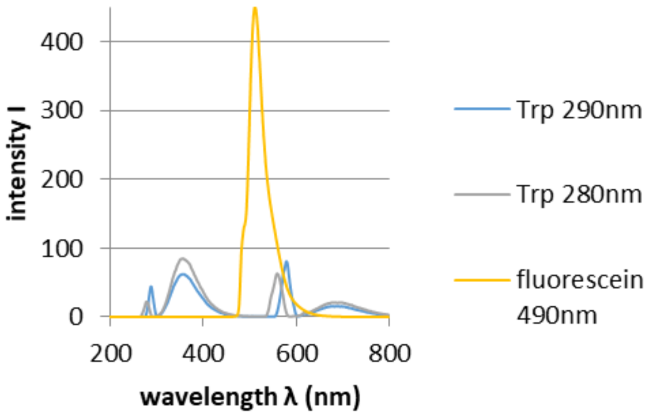

Figure S9. Fluorescence excitation and emission spectra of tryptophan and fluorescein in water.

The table S1 shows the quantum yield of fluorescein and tryptophan and the refractive index of both compounds.

$$Qs = \mathrm{Qr} * \frac{Ds}{Dr} * \frac{Ar}{As} * \frac{Ir}{Is} * \frac{\mathrm{ns}^2}{nr^2}$$

The Table S1 and Table S2 show the reference data for two fluorescence references considered to carry out the quantum yield calculations and the refractive index of common solvents.

Table S1. Quantum yield and refractive index of fluorescein and tryptophan

	References data	
	Quantum yield Q_r	Refractive index n_r
Fluorescein	0.82	1.33
Tryptophan	0.13	1.754

Table S2. Refractive index of several solvents

	**Refractive index of solvents (n_s)**
DMSO	1.479
water	1.33
toluene	1.497
methanol	1.329

Table S3 lists the fluorescence data extracted from the fluorescence spectra of bulk powder particulate $Y_{0.9}Eu_{0.1}VO_4$ (Figure S6b)

Table S3. Fluorescence data of bulk powder particulate $Y_{0.9}Eu_{0.1}VO_4$ at different concentrations in water

	**λ (nm)**	**A**	**Total area**	**I (excitation peak)**	**φs(without I)**	**φs**
**Trp**	280	0.0555	2968.3981	37.98		
**0.025 mg/mL**	280	0.1159	495.13	142.36	0.00551102	0.001470276
**0.05 mg/mL**	280	0.1159	549.2893	195.74	0.006113837	0.001186286
**0.01 mg/mL**	280	0.1328	430.1346	127.51	0.004178328	0.001244552

Stability of the luminescence with time

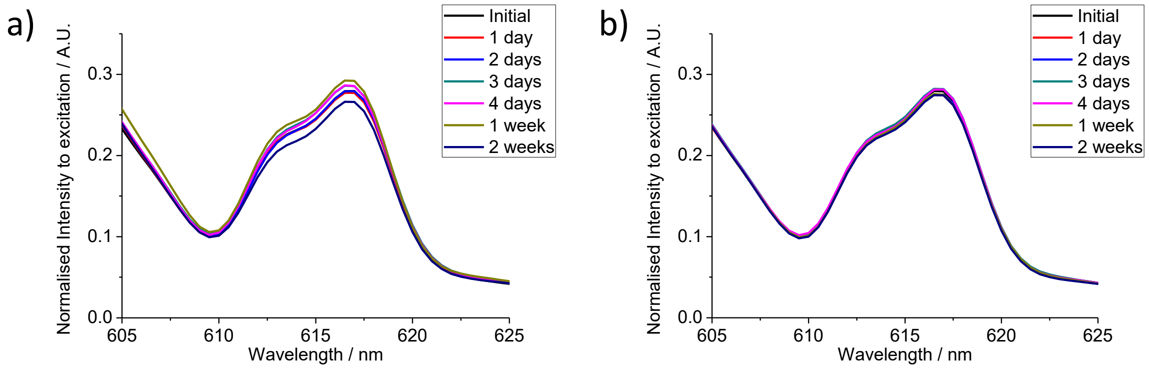

Figure S10. Luminescence spectra of bulk powder particulate $Y_{0.9}Eu_{0.1}VO_4$ (λ_{ex} = 290 nm) in Milli-Q water 0.1 mg/mL stored in a glass vial, protected from light a) at room temperature and b) at 4 °C up to 2 weeks.

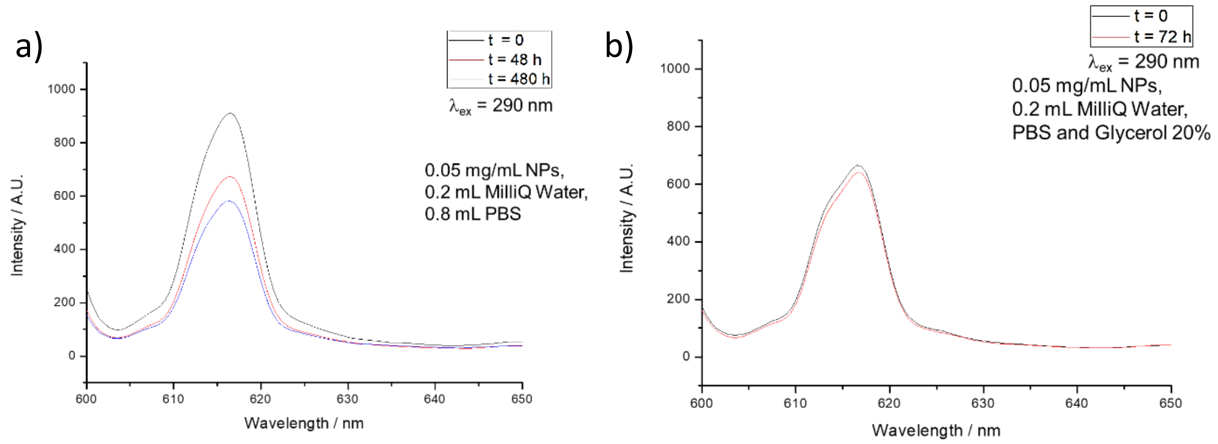

Figure S11. Luminescence spectra of bulk powder particulate $Y_{0.9}Eu_{0.1}VO_4$ (λ_{ex} = 290 nm) in a) PBS 0.05 mg/mL stored in a glass vial, protected from light at room temperature up to 20 days and b) PBS with glycerol 20% 0.05 mg/mL stored in a glass vial, protected from light at room temperature up to 3 days.

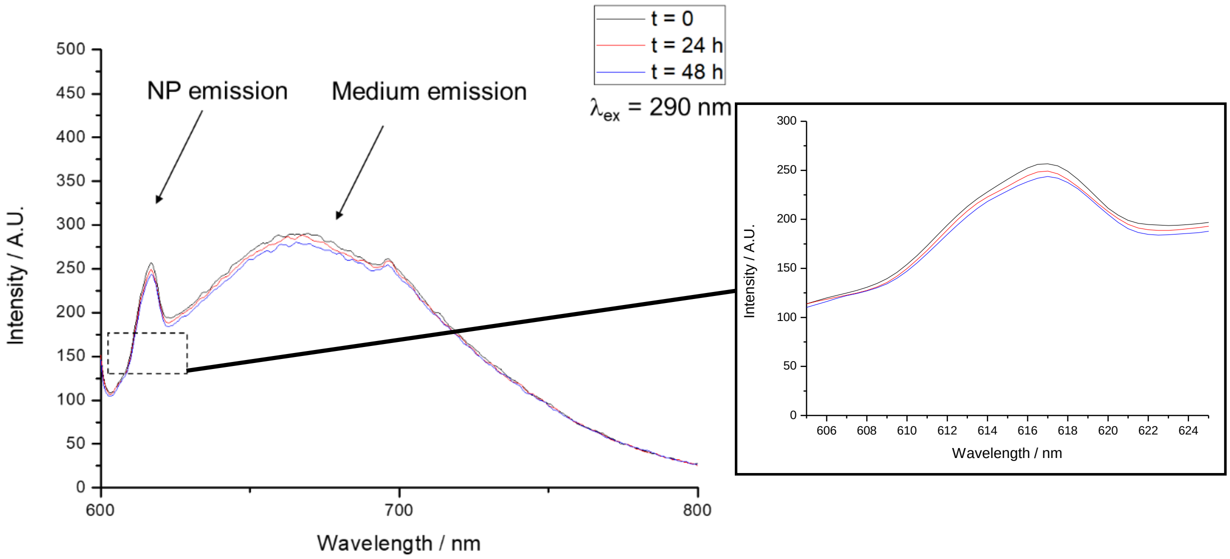

Figure S12. Luminescence spectra of bulk powder particulate $Y_{0.9}Eu_{0.1}VO_4$ (λ_{ex} = 290 nm) in cell medium (MEM) (0.8 mL MEM 10% FBS + 0.05 mg/mL NPs, 0.2 mL Milli-Q water) protected from light at room temperature up to 2 days.

Fluorescence microscopy

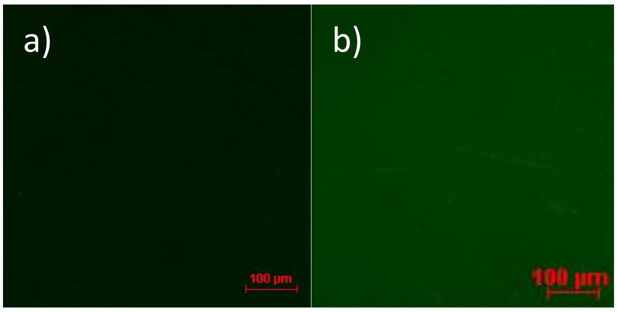

Figure S13. Representative fluorescence of a) Milli-Q water and b) Milli-Q water with 0.25 mg/mL of bulk powder particulate $Y_{0.9}Eu_{0.1}VO_4$ nanomaterials (green channel, λ_{ex}= 488 nm).

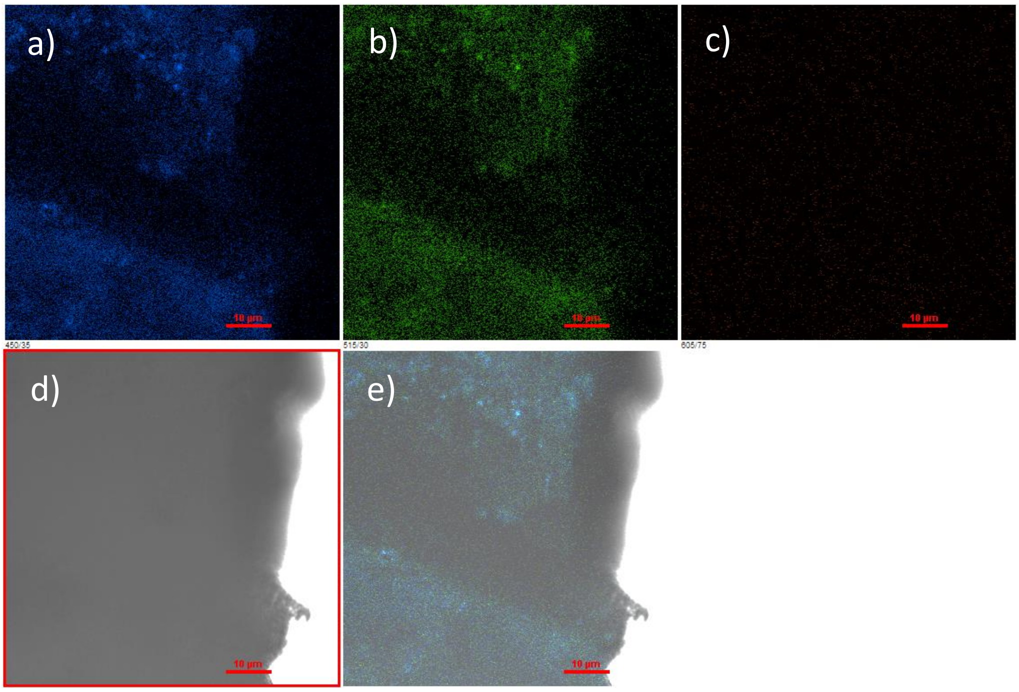

Figure S14. Representative confocal fluorescence of bulk powder particulate $Y_{0.9}Eu_{0.1}VO_4$ nanomaterials deposited on a glass slide a) blue channel; b) green channel; c) red channel; d) DIC image and e) overlay of the blue-green channels; λ_{ex} = 488 nm. Scale bar: 10 μm.

Cell studies

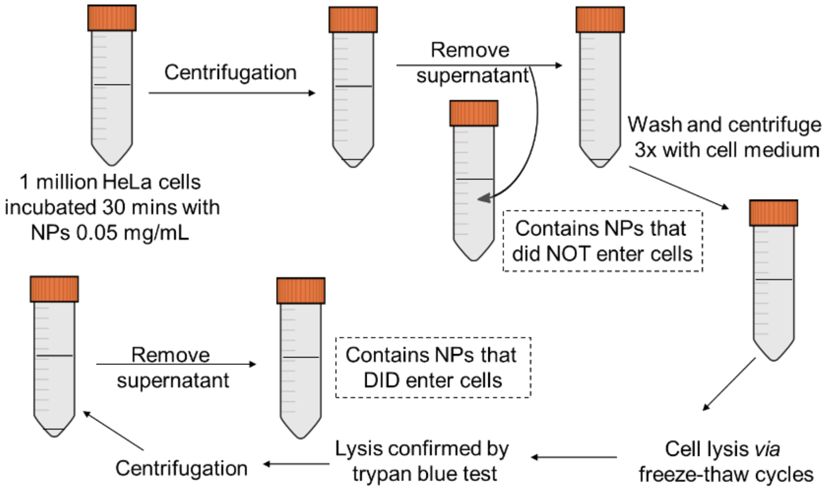

Scheme S1. Cell uptake experiment.

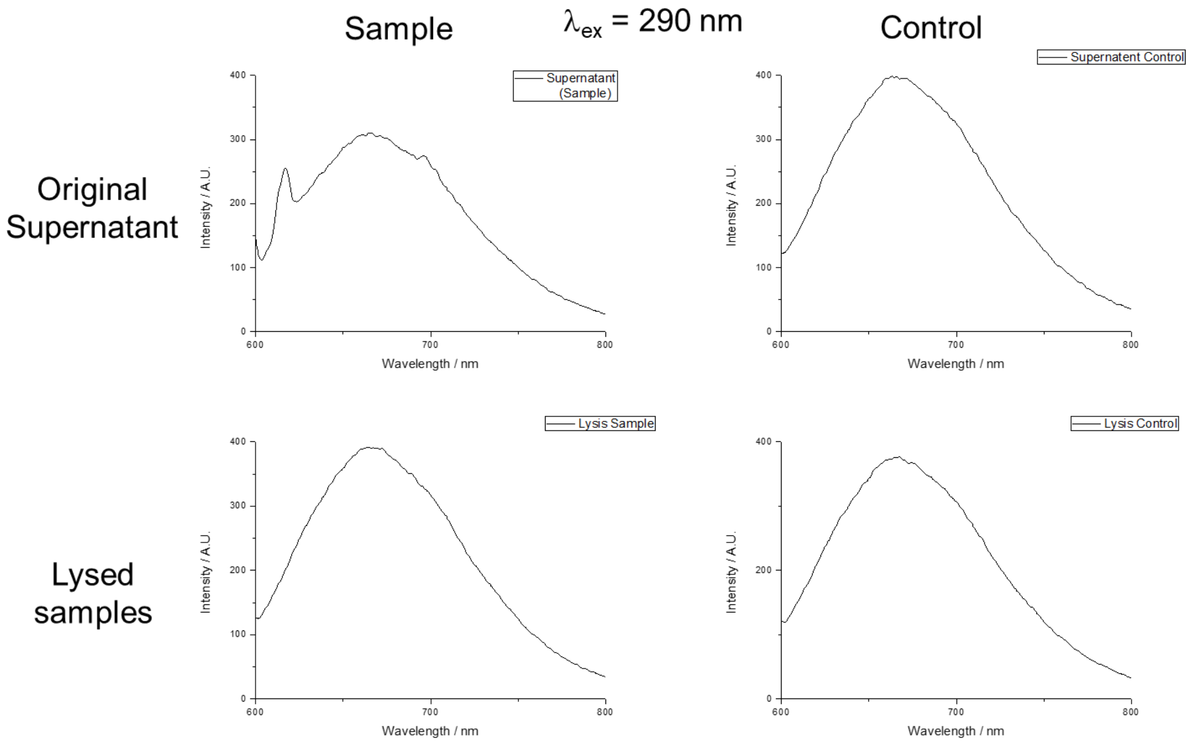

Figure S15. Luminescence spectra of bulk powder particulate $Y_{0.9}Eu_{0.1}VO_4$ nanomaterials and control samples (λ_{ex} = 290 nm).

As it can be observed in the Figure S16, no incorporation of bulk powder particulate $Y_{0.9}Eu_{0.1}VO_4$ into the cell takes place. This was also confirmed by confocal fluorescence microscopy (Figure S17).

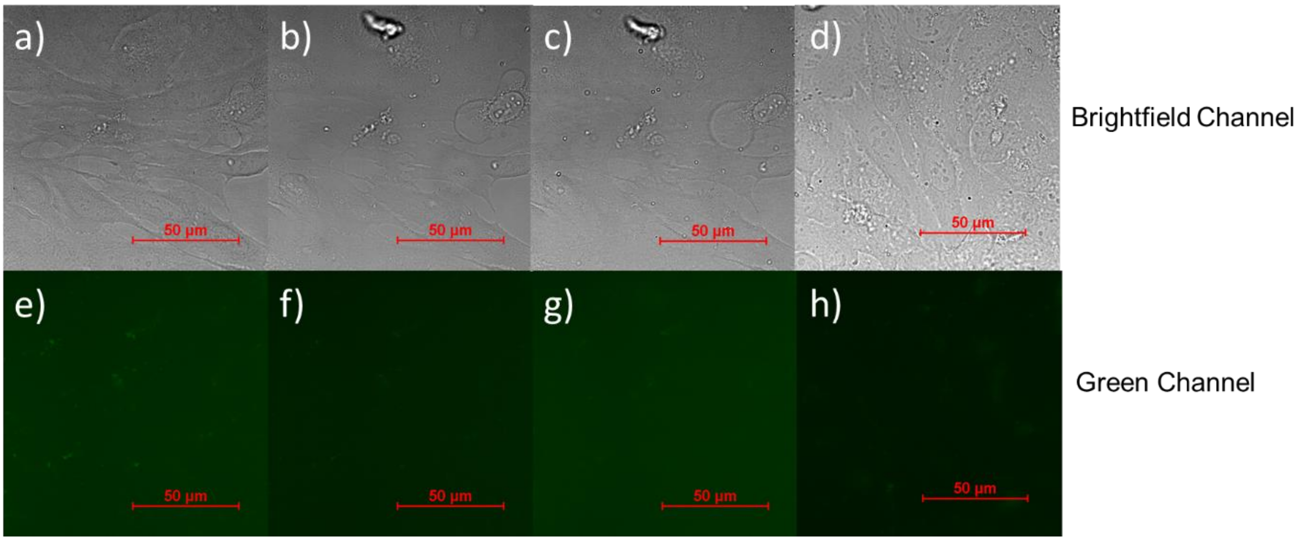

Figure S16. Fluorescence image of live HeLa cells incubated with bulk powder particulate $Y_{0.9}Eu_{0.1}VO_4$ (0.05 mg/mL) at a) and e) t = 0 min (without nanoparticles), b) and f) t = 15 min, c) and g) t = 60 min, and d) and h) t = 360 min. λ_{ex} = 488 nm. Scale bar: 50 μm.

In addition, it has been proven experimentally that, after 6 hours of incubation, there is practically no incorporation of uncoated bulk powder particulate $Y_{0.9}Eu_{0.1}VO_4$ into the cell, and the non-toxicity of the nanoparticles Figure S17.

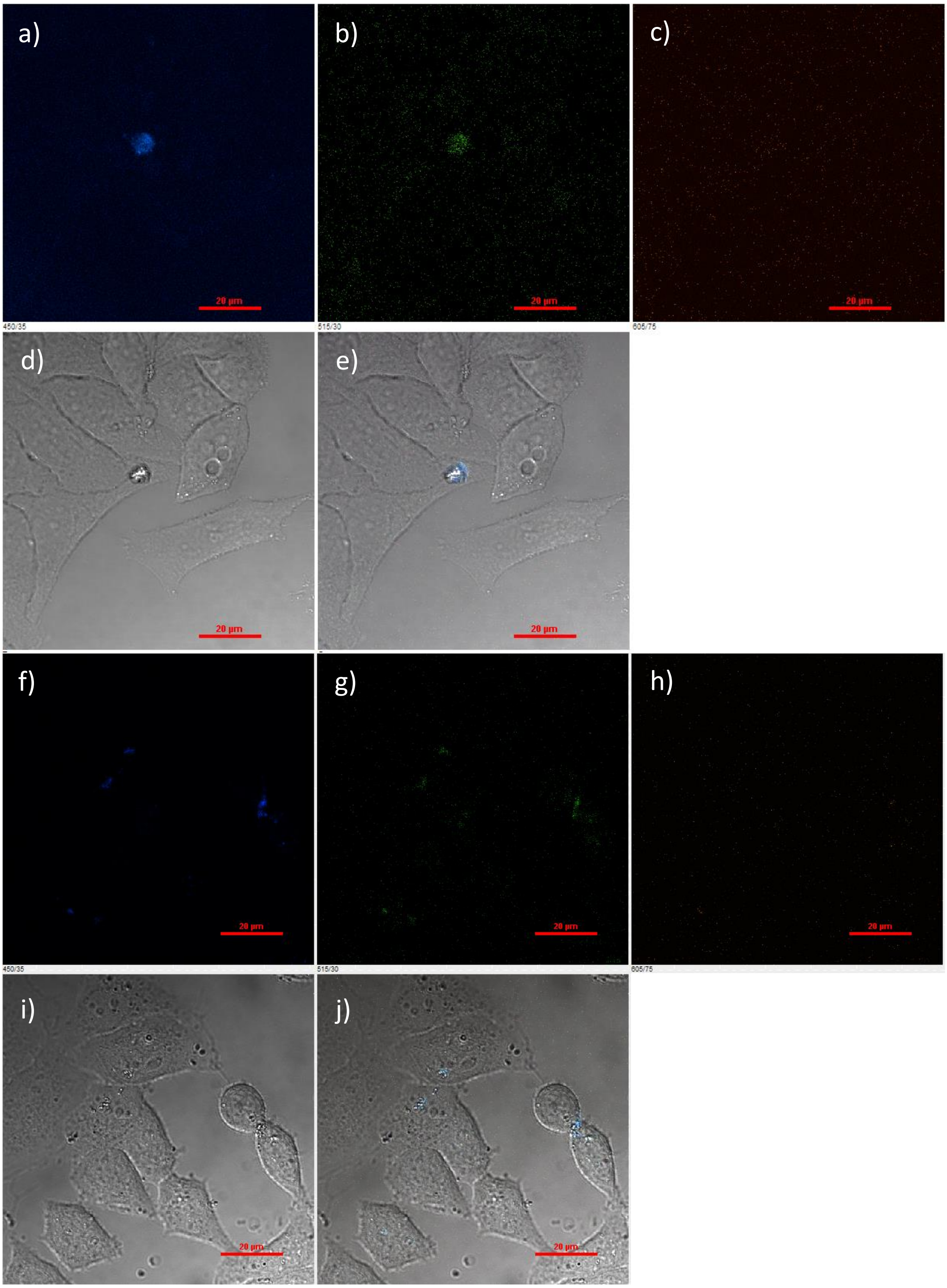

Figure S17. Confocal image of live HeLa cells incubated (15 min) with bulk powder particulate $Y_{0.9}Eu_{0.1}VO_4$ (0.05 mg/mL) a) blue channel; b) green channel; c) red channel; d) DIC image and e) overlay of the blue-green channels and live HeLa cells incubated (360 min) with bulk powder particulate $Y_{0.9}Eu_{0.1}VO_4$ (0.05 mg/mL) f) blue channel; g) green channel; h) red channel; i) DIC image and j) overlay of the blue-green channels; λ_{ex} = 488 nm. Scale bar: 20 μm.

PEG-ylation Procedures

The amino PEG precursors and the aminooxy H_2N-PEG-OC_3H_6-COOH polymer with Mw ca. 2000 g/mol, 3000 g/mol and 5000 g/mol were purchased from Rapp Polymere or NanoCS and functionalised in-house through the synthetic approaches described below, giving rise to Compounds Type I and II below.

In parallel experiments the surface of the bulk powder material $Y_{0.9}Ln_{0.1}VO_4$ (denoted Eu:YVO4) and of the core-shell $Y_{0.9}Ln_{0.1}VO_4@SiO_2$ nanoparticles was functionalized with functional PEGylated silane polymers with Mw of ca. 2000, 3000 and 5000 Da, whereby n were of 40 (**$C_7H_{16}NO_4Si(C_2H_4O)_{40}CH_3O$),** 60 (for **$C_7H_{17}N_2O_4Si(C_2H_4O)_{60}C_4H_7O_3$)**, and finally **$C_7H_{17}N_2O_4Si(C_2H_4O)_{100}C_4H_7O_3$** of ca. 100 PEG mer units), through a number of optimised routes, described below. In case of the bulk powder material $Y_{0.9}Ln_{0.1}VO_4$ (denoted Eu:YVO4) one-pot reaction functionalisation, to prevent the possibility that the -NCO functional reactants **(2)** may still be present on the NP surface, the quenching reaction with the amino PEG precursors (compounds Type I) were carried out in situ, aiming to attach these directly on the -NCO terminal groups on the NPs. These type of reactions were also carried out for the linker functionalisation optimisation strategies, applied in a stepwise reaching, after the isolation of the molecular **compounds of types I and II**, characterised by IR, mass spectrometry and GPC.

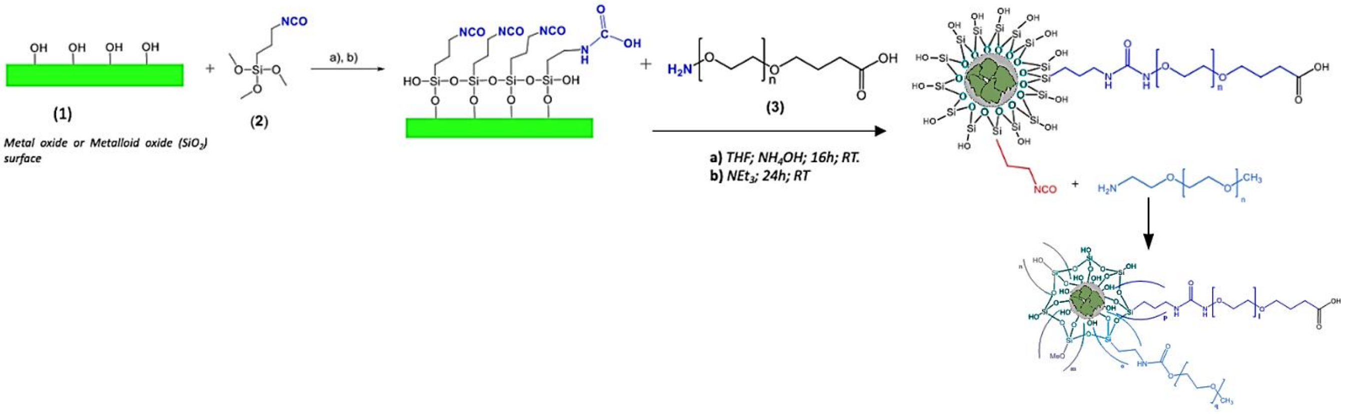

Scheme S1. General functionalisation of bulk powder materials, in one-pot reaction approach used for the optimization reactions for functionalization with -COOH groups followed by quenching in situ of -NCO residues.

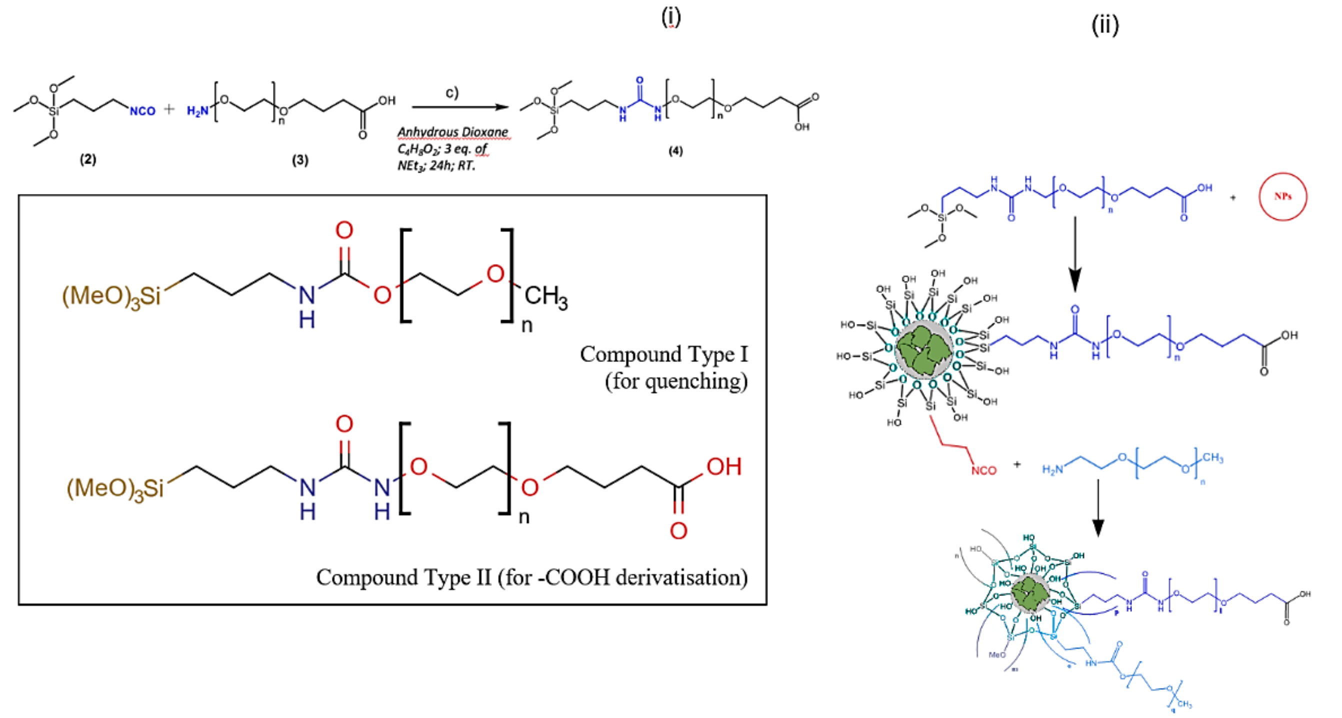

Scheme S2. Stepwise functionalization protocol for optimization reactions: **(i)** Formation of functional linker molecules followed by **(ii)** Direct NPs surface functionalization and quenching, adopted hereby as the functionalisation approach for direct functionalisation of $Y_{0.9}Eu_{0.1}VO_4$ bulk powder nanomaterial used for kinetic stability investigations. *Box:* Structures of linkers precursors and quenchers isolated, Types **I** and **II,** whereby compounds type **I** (n = 40, 60 or 100-111 range) were typically used in the quenching reactions. The functionalised linkers (with PEGs 2 kDa, 3 kDa and 5kDa) denoted compounds type II and derived from the aminooxy H_2N-PEG-O-C_3H_6-COOH (with Mw ca. 2 kDa, 3 kDa and 5 kDa) were isolated and characterised and used directly as molecular linkers for the nanophosphors conjugation and introduction of -COOH units.

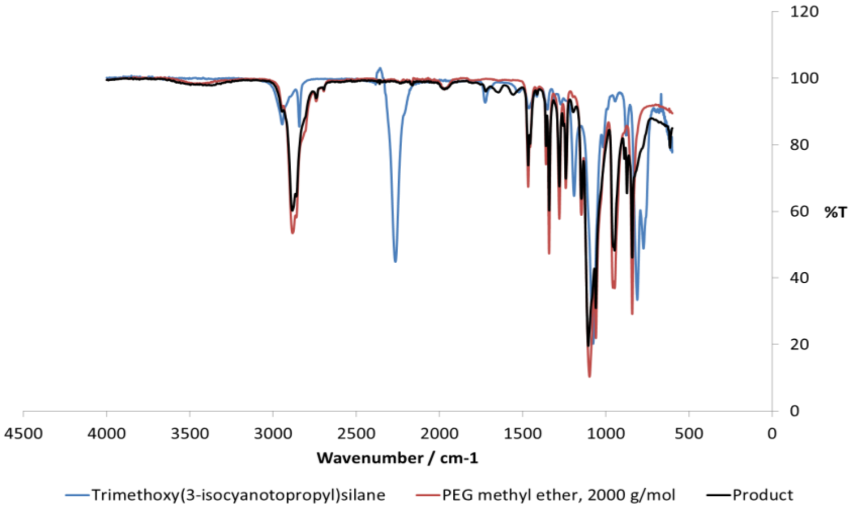

Figure S18. FT-IR spectra of Polyethylene methyl ester (red line), Trimethoxy(3-isocynotopropyl)silane (blue line) and Trimethoxy(3-ureapropyl)polyethylene glycol-silane (black line).

Reaction approach 1

100 mg nanophosphor (bulk, solid powder) was placed in a pressure tube, 10 mL dry, degassed THF and 10 µL aqueous ammonia (34%) was added and stirred for 10 minutes. 1.1 g Trimethoxy(3-isocyanotopropyl)silane was added and the reaction mixture was stirred overnight at 66°C. The product was centrifuged at 3000 rpm for 10 minutes and subsequently washed 3x with dry THF. After removal of THF the nanophosphors were analysed by IR:

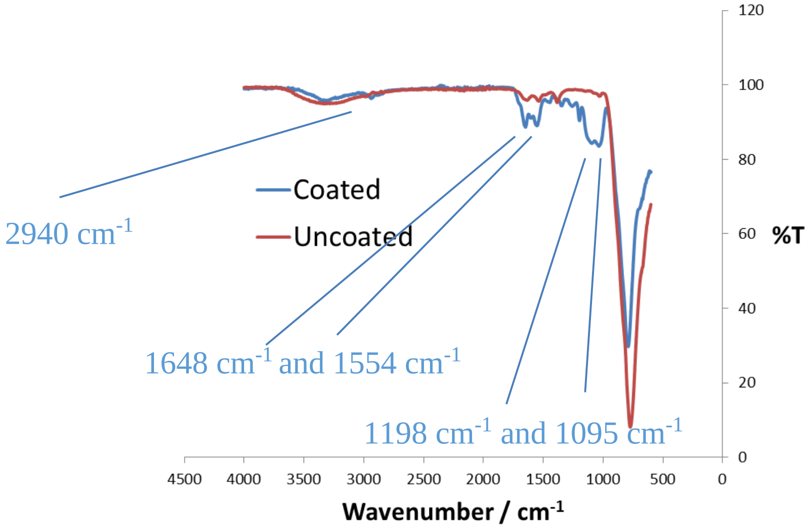

Figure S19. FT-IR of coated and uncoated $Y_{0.9}Eu_{0.1}VO_4$ nanoparticles, whereby for the coated nanoparticle IR(neat): 3328, 2940, 1648, 1601, 1554, 1445, 1409, 1378, 1347, 1309, 1257, 1198, 1095, 1035, 791, 701, 669. Uncoated, IR(neat): 3324, 1634, 1536, 1384, 1030, 773 cm^{-1}.

A band at ca. 2300 cm^{-1} would be expected for an isocyanate group, which is clearly not present. However, a band and 1095 cm^{-1} were detected which are indicative of Si-O, showing that coating has occurred (Figure S19). Therefore, it appears that the -NCO group has hydrolysed, leaving an amine group. Bands at 1648 cm^{-1} and 1554 cm^{-1} (N-H bend region) and at 1198 cm^{-1} (C-N stretch region) are confirmative of the presence of a –NH_2 group. There is also a band at 2940 cm^{-1} not visible in the uncoated, which could be indicative of one of the two expected the N-H stretch (despite being a little low). The other N-H stretch may be overlapping with the broad band occurring

in the uncoated nanophosphors and is therefore not easily visible. To avoid the observed hydrolysis a one-pot approach and pre-reacting approach were carried out.

A Schlenk was pre-silylated using dimethyldichloride silane *in vacuo* using a desiccator for 2 hours and was then moved to an oven for 2h at 200°C. 10 mL dry, THF and 10 µL aqueous ammonia (34%) was added followed by 100 mg Eu:YVO_4 nanophosphor under a flow of nitrogen and stirred for 1h. 1 mL

Trimethoxy(3-isocyanotopropyl)silane was added and the reaction mixture was stirred overnight at room temperature. 100 mg of the 5,000 g/mol (NH_2-PEG-COOH)HCl was added followed by 1 eq. of triethylamine under a flow of nitrogen. After 20h 250 mg of Poly(ethylene glycol) monomethyl ether (5,000 g/mol) was added under a flow of nitrogen (**Scheme S3**). This was allowed to react for 5h and then was centrifuged at 3000 rpm for 5 minutes and subsequently washed 3x with dry THF. After removal of THF the nanophosphors, the supernatant was analyzed by fluorimetry in Milli-Q water (**Figure S19**). The characteristic spectrum can be visualized here (λ_{ex} = 290 nm), where there was an absence of the emission peak observed in poorly dispersed coated nanoparticle (a broad peak ca. 350 nm). This therefore indicates that there is a good dispersability in THF and in water.

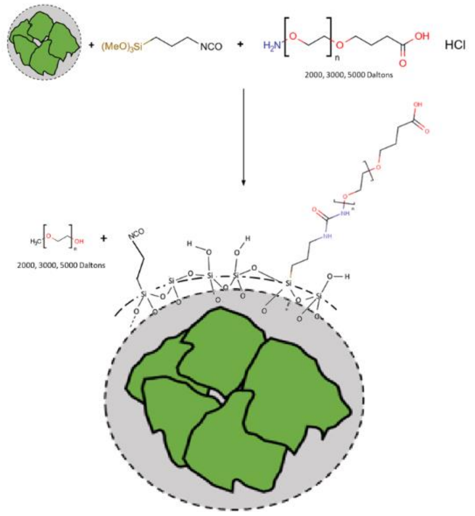

Scheme S3. Reaction scheme for the optimised 1-pot synthesis to yield coated $Y_{0.9}Eu_{0.1}VO_4$ nanoparticles *via* an isocyanate functionalized silane, and generalised to the range of functional PEG linkers.

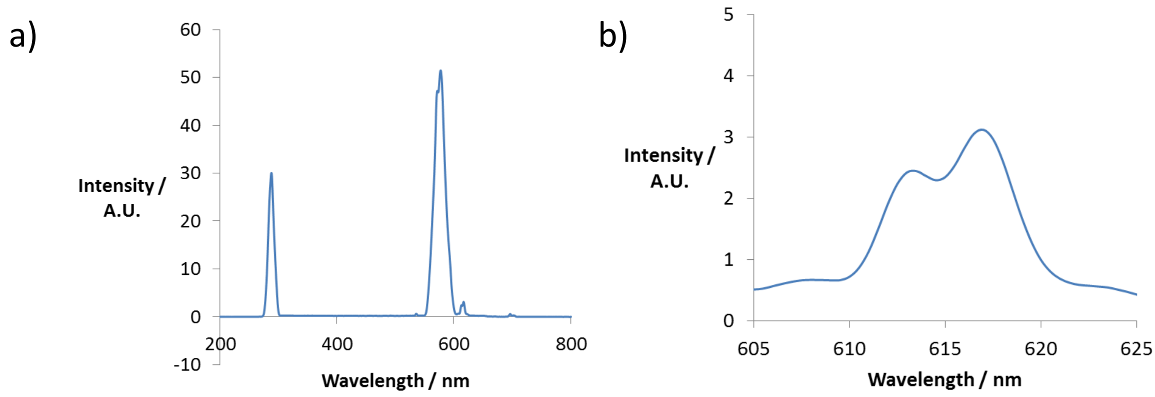

Figure S20. Luminescence spectra of the $Y_{0.9}Eu_{0.1}VO_4$ product, coated with PEG5000 linker in 2 mg/mL in Milli-Q water of scheme 1, a) 200 nm to 800 nm, b) 605 nm to 625 nm, λ_{ex} = 290 nm.

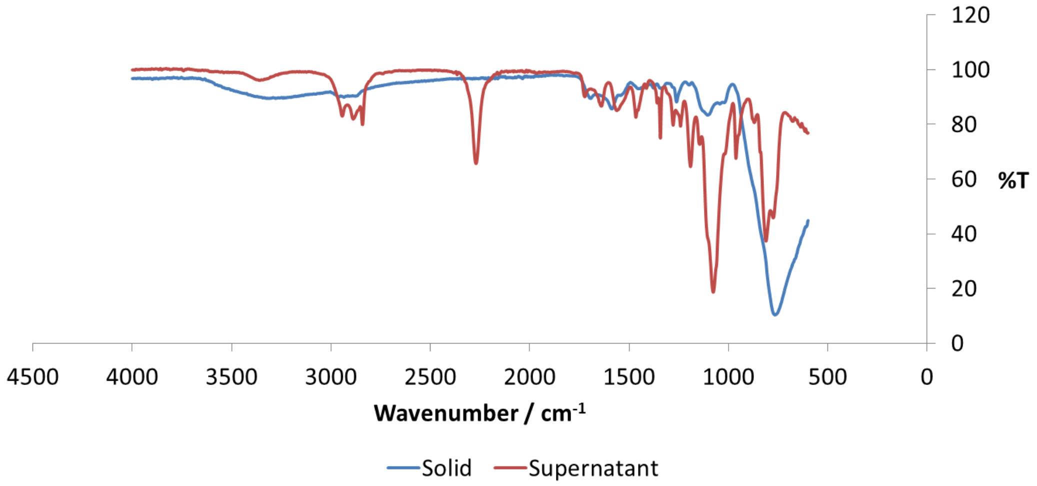

Figure 21. IR spectra of the supernatant (IR(neat): 3361, 2944, 2889, 2842, 2271, 1723, 1640, 1563, 1466, 1413, 1360, 1343, 1279, 1242, 1192, 1145, 1077, 963, 871, 812, 775, 676, 638, 616 cm^{-1}) and of the solid (IR(neat): 3315, 2963, 2935, 2877, 1693 ,1587, 1542, 1448, 1420, 1377, 1339, 1262, 1198, 1106, 1044, 1021, 767) from the reaction described in Scheme S3 (for PEG5000).

Quenching of reaction shown in Scheme S3:

To the supernatant a further 250 mg of Poly(ethylene glycol) monomethyl ether (ca. 5,000 g/mol, denoted MeO-functional PEG5000) and 10 mL of anhydrous dioxane. This was gently heated into solution and stirred at rt for 24h. The flask was dried *in vacuo* and an IR spectrum was measured. The resultant product was not found to be fully reacted (due to the presence of the isocyanate peak at 2271 cm^{-1}), therefore the process above was repeated with another 250 mg of Poly(ethylene glycol) monomethyl ether (5,000 g/mol) in anhydrous dioxane. After drying, the IR spectrum showed that no isocyanate remained (**Figure S22**).

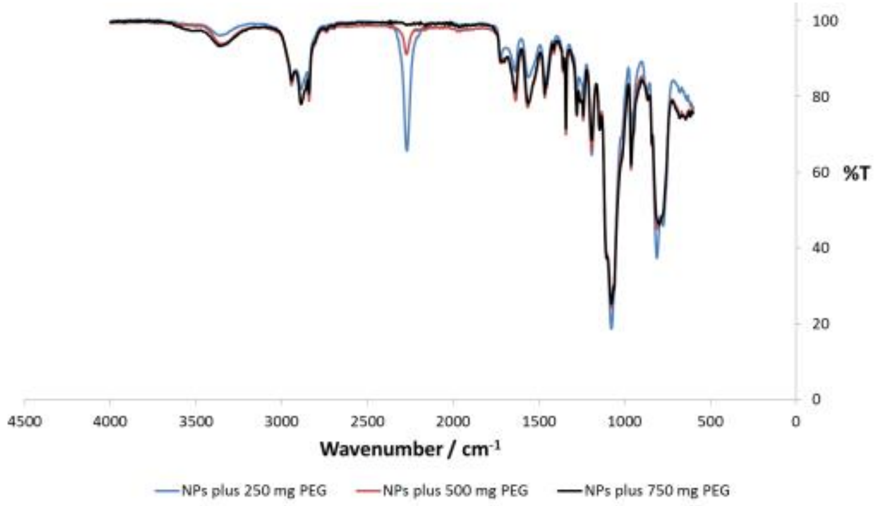

Figure S22. FTIR spectra of product of reaction described in Scheme S2 (blue), with an extra 250 mg of PEG methyl ether added (totalling 500 mg) (red) IR(neat): 3354, 2943, 2888, 2841, 2741,2695, 2272, 1724, 1636, 1566, 1466, 1455, 1413, 1360, 1343, 1279, 1242, 1192, 1147, 1106, 1078, 962, 866, 841, 811, 676, 643, 617 cm^{-1}) and with a total of 750 mg (black) (IR(neat): 3369, 2942, 2888, 2842, 2741, 2696, 1723, 1705, 1639, 1562, 1466, 1413, 1360, 1343, 1279, 1257, 1242, 1192, 1146, 1106, 1077, 1061, 962, 864, 841, 799, 679, 644, 616 cm^{-1}).

The product from the reaction illustrated in Scheme S3, which was fully quenched, was dialysed using 7 rounds of washing with Milli-Q water, using 250 mL each, whereby each 250 mL portion was exposed to the dialysis membrane for ca. 60 minutes and collected.

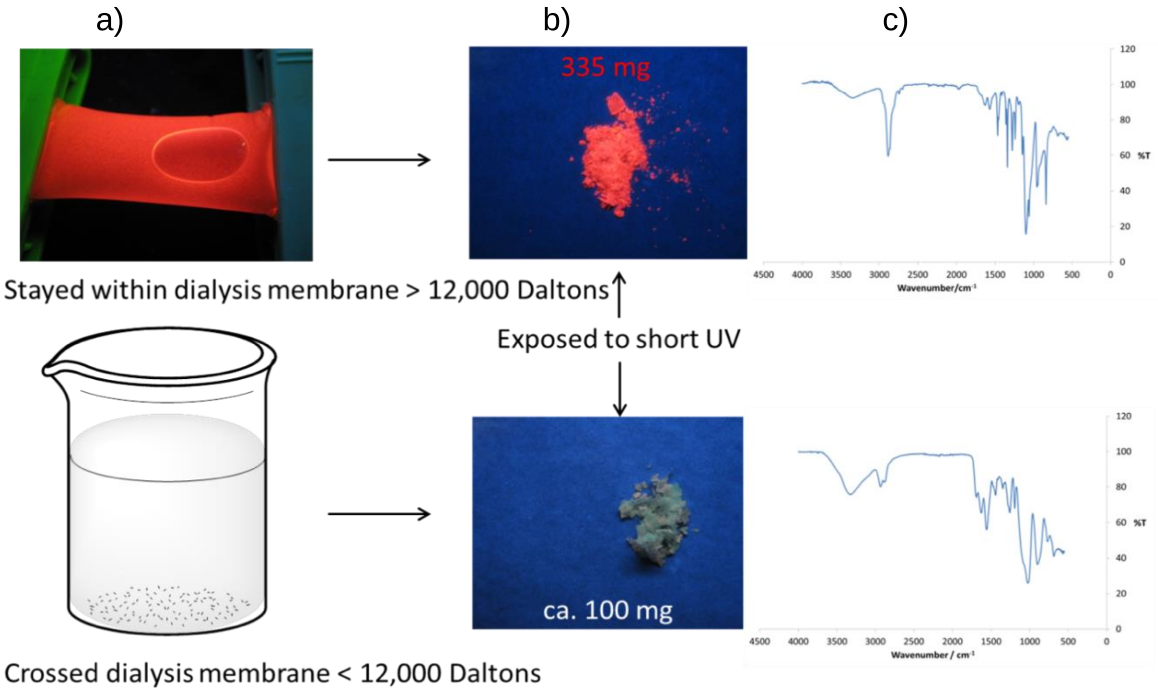

Figure S23. Overview of purification process of PEG5000-linked bulk-powder nanophosphors, a) dialysis, b) UV exposure, c) IR spectroscopy. Within the dialysis membrane IR (neat): 3347, 2943, 2885, 2742, 2696, 1700, 1628, 1568, 1467, 1455, 1413, 1360, 1341, 1279, 1241, 1196, 1146, 1100, 1060, 958, 947, 842, 777, 685 cm^{-1}; dialysate IR(neat): 3329, 2931, 2883, 1690, 1628, 1558, 1474, 1444, 1413, 1349, 1330, 1260, 1196, 1024, 899, 767, 686 cm^{-1}.

Both the liquid which remained within the dialysis membrane and the dialysate were isolated *in vacuo* (Figure S23). The IR spectra of the un-purified product, the dialysate and that, which stayed within the membrane, are compared below (Figure S24). Importantly the FTIR spectrum of the purified product is notably different from the unreacted nanophosphor and contains a significant band at 2890 cm^{-1} (Figure S24) which is attributable to the carboxylic acid. Furthermore, the intense luminescence of which stayed within the membrane when irradiated with short UV light (ca. 254 nm) indicates that this reaction was successful and yielded 335 mg of coated $Y_{0.9}Eu_{0.1}VO_4$ nanoparticles.

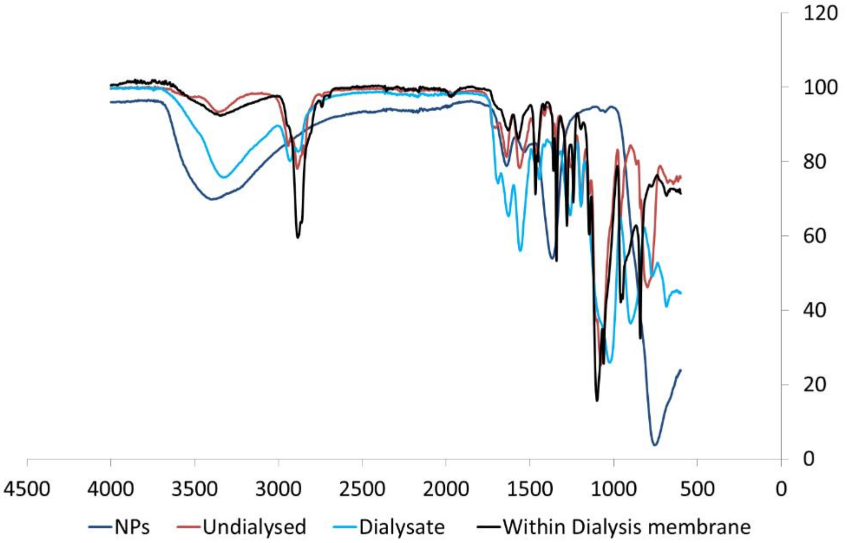

Figure S24. IR spectra of bulk powder particulate $Y_{0.9}Eu_{0.1}VO_4$ (dark blue), undialyzed solid (red), dialysate (light blue), the isolate remaining within the dialysis membrane. IR bands listed in **Figures S22-S23**.

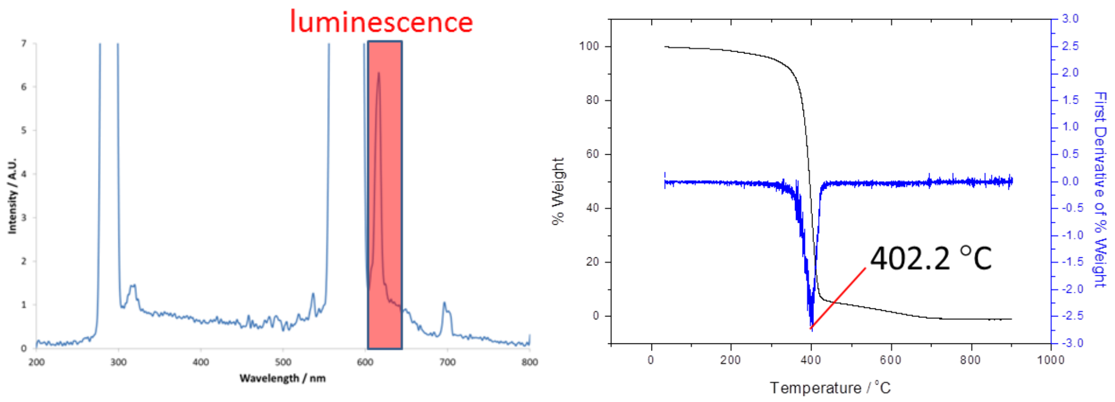

Figure S25. (left) Luminescence emission spectrum of coated bulk powder particulate $Y_{0.9}Eu_{0.1}VO_4$ with the functional 5 kDa PEG at 0.1 mg/mL (after dialysis), with an emission intensity of 6.3 A.U. (Right) TGA of coated bulk powder particulate $Y_{0.9}Eu_{0.1}VO_4$ with 5 the kDa PEG.

<u>Approach 2: Test reaction approach for alternative PEG linkers functionalisation</u>

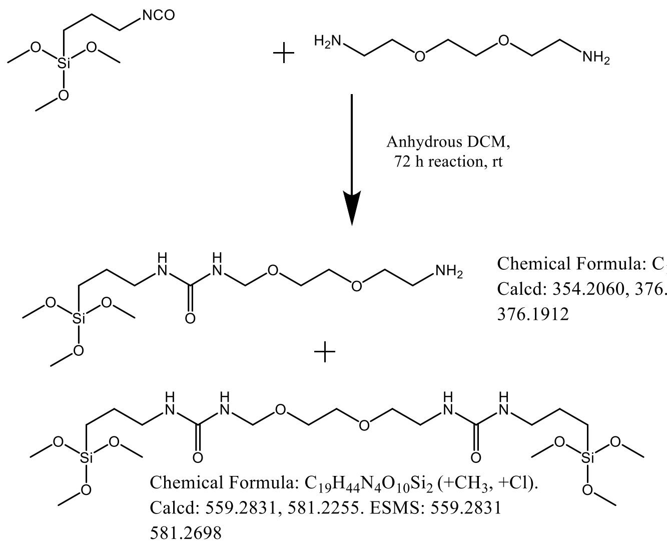

Scheme S4. A model reaction with a short amine-functionalised PEG, leading to a mono-substituted siloxane compound.

Another approach was attempted whereby reacting the silane isocyanate with the NH_2-PEG-tBoc protected amine prior to coating to the nanophosphor. Trimethoxy(3-isocyanotopropyl)silane, 1.084 g (0.0053 mmols) was diluted in 100 mL anhydrous DCM and added dropwise (over 8 h) to 0.783 g (0.0053 mmols) and allowed to stir over weekend (Scheme S4). DCM was removed *in vacuo* and an oily product was analysed by ESMS and ^{1}H and ^{13}C NMR. Mass spectrometry confirmed that there was a mixture of the mono(substituted) and di(substituted) silane (peaks ionised in positive mode as in the reaction scheme above). Importantly, there was no significant peak according to MS corresponding to the silane starting material (or its hydrolysed form) (Figure S26) as was there no peak in the region of 120 ppm in the ^{13}C NMR (75.5 MHz).

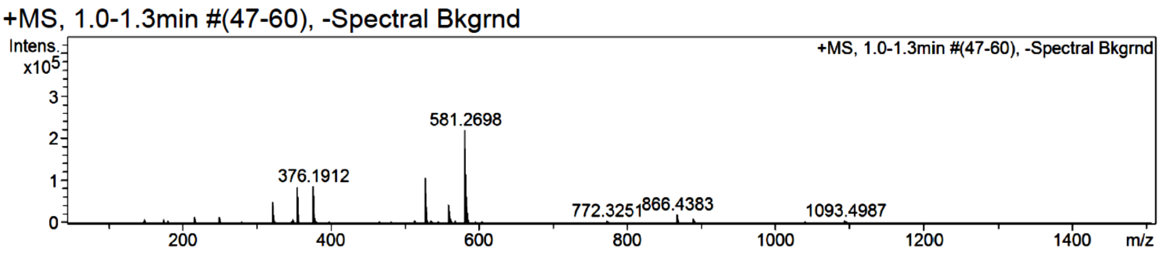

Figure S26. ES-MS spectrum of the obtained product.

Alternative functionalisation of linkers - reaction approach 3

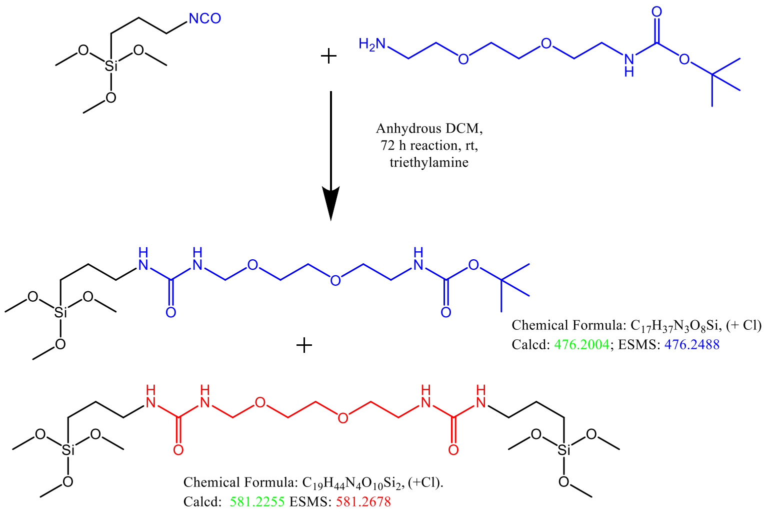

Scheme S5. A model reaction with a short t-boc protected-amine-functionalised PEG, with the purpose of synthesising a mono-substituted siloxane compound.

277.3 mg (1.351 mmols) Trimethoxy(3-isocyanotopropyl)silane, was added to 335.4 mg (1.351 mmols) of a short t-Butoxycarbonyl-Amine-PEG-Amine, with vast excess of triethylamine (136.8 mg) in anhydrous dichloromethane (Scheme S5). A preliminary ES-MS demonstrated that a mixture of the desired product and undesirable bis(substituted) product were formed (Figure S27).

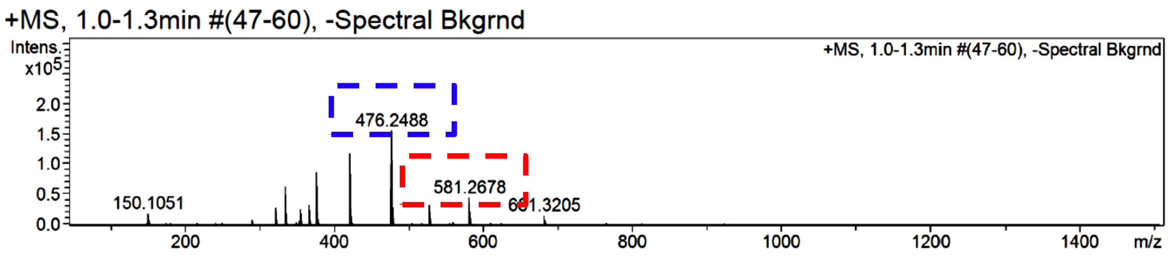

Figure S27. ES-MS of the product from reaction approach 3, showing a mixture of the desired mono(substituted) product (blue) and the by-product (red).

Alternative reaction approach 4

31.8 mg (29.4 µL, 0.1551 mmols) trimethoxy(3-isocyanotopropyl)silane was added to 0.1 g (0.1551 mmols) of t-Butoxycarbonyl-Amine-PEG-Amine (M_r = 644.7 g/mols) and to 3 equivalents of triethylamine. As was observed with model reaction 3 a preliminary ES-MS demonstrated that a mixture of the desired product and undesirable bis(substituted) product were formed (Figure S28).

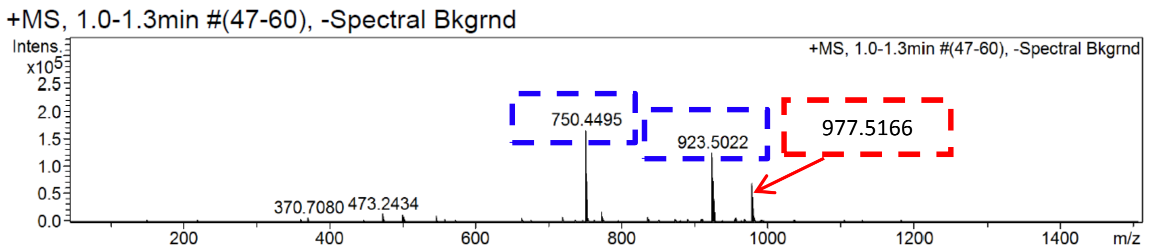

Figure S28. ES-MS of reaction approach 4, showing a mixture of the desired mono(substituted) products (protected and de-protected) (blue) and a small amount of the by-product (red).

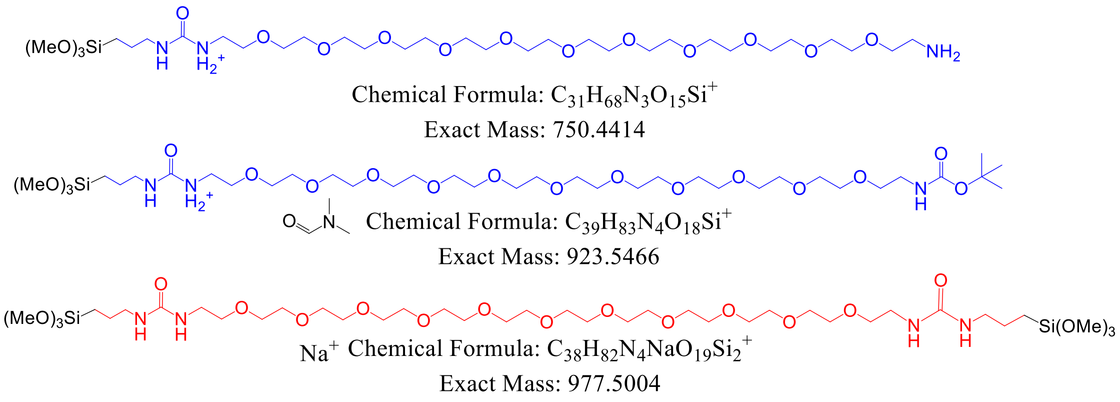

Figure S29. Figure outlining the likely products of reaction approach 4

Both t-Boc protected amine reaction approaches 3 and 4 resulted in deprotection, despite being carried out under basic conditions. It can be speculated that improved results may be achieved, by rinsing glassware with base prior to the start of the reaction and addition of the t-boc protected amine as the last component.

Alternative reaction - approach 5

Following the method of Lam *et al.*[1], succinic anhydride (1.0 g, 9.99 mmols) in 100 mL 1,4-dioxane was added dropwise on ice to 2,2′-(Ethylenedioxy)bis(ethylamine) (1.703 g, 11.5 mmols) over 30 minutes. This was allowed to warm to room temperature and rotary evaporated. Saturated aqueous Na_2CO_3 was added followed by 200 mL of diethyl ether. The following mixture was extracted and washed a second time with ether. The aqueous phase was dried *in vacuo* and a mixture of 1:1 acetone: ethanol was added (100 mL). This was subsequently filtered and washed again with 1:1 acetone: ethanol (100 mL). The solvent was removed *in vacuo* leaving an oily solid. ES-MS appears to show that the most significant peak was the unwanted di-substituted PEG, the length of PEG may be important in selectivity of this reaction (Figure S30). The peak shown is in negative mode showing the peak expected in positive mode – the spectrum may require re-calibration. It also includes the peak plus a sodium accurate to 2 decimal places.

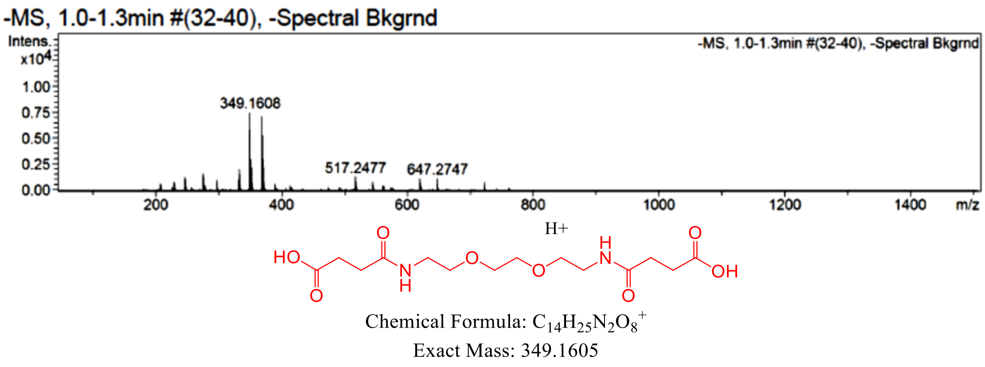

Figure S30. ES-MS of model reaction approach 5, which appears to show the presence of the di-substituted PEG Composites - with 3 kDa PEG and 5 kDa PEG – and which were characterised by IR (figure S31).

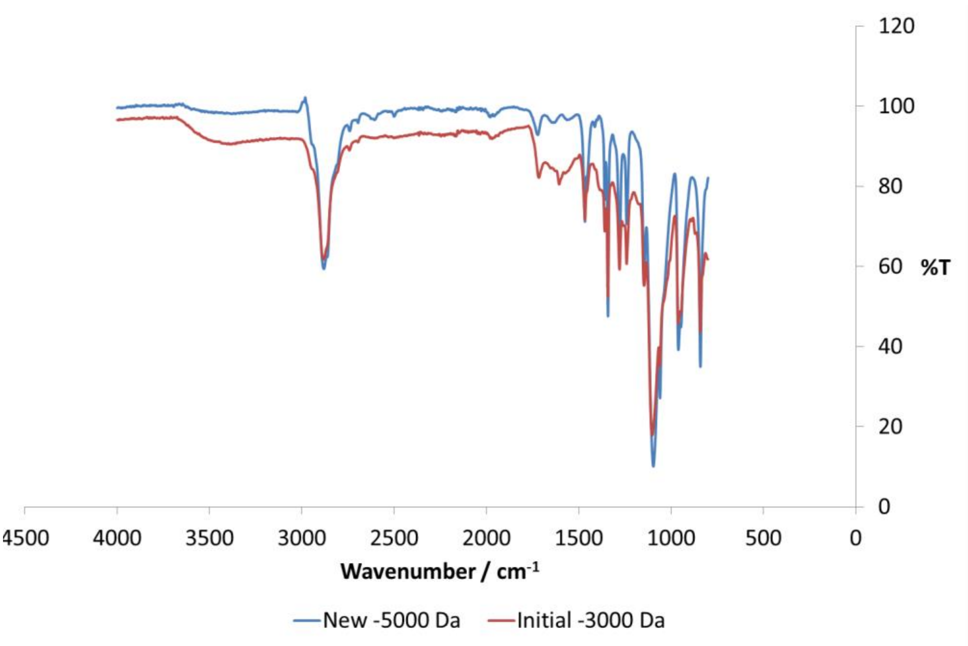

Figure S31. FT-IR spectra of trimethoxy(3-isocyanotopropyl)silane and product of reaction B: Compound with 3 kDaPEG IR(neat): 3426, 2884, 2743, 2696, 1717, 1653, 1606, 1575, 1467, 1360, 1342, 1280, 1241, 1147, 1103, 1061, 961, 870, 841, 801, 771, 746, 731, 696 cm^{-1}. Compound with 5 kDaPEG IR(neat): 2944, 2881, 2863, 2741, 2696, 2606, 2500, 1980, 1722, 1640, 1559, 1467, 1456, 1360, 1342, 1279, 1241, 1146, 1096, 1060, 961, 947, 841 cm^{-1}.

__Reactions using protocols involving a pre-reacting to silane step__

To allow both more control over the reaction and analysis *via* additional methods an approach whereby the silane was reacted to the PEG prior to coating was explored.

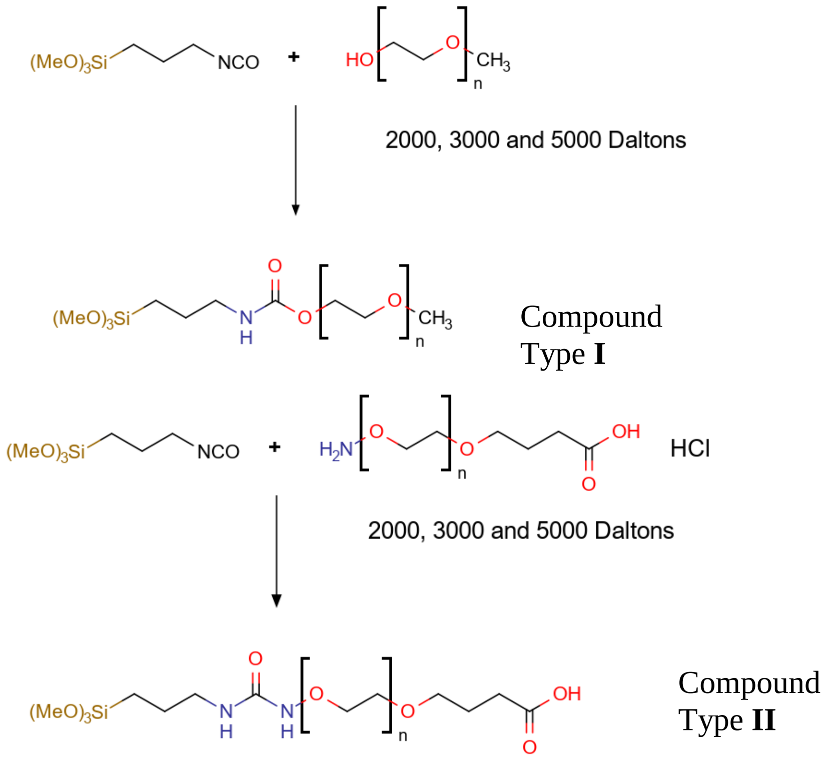

Scheme S6. Reaction to enable a controlled ratio of non-reacting PEG and to hetero-labelled PEG-amine.

Reaction A: A Schlenk was pre-silylated using dimethyldichloride silane *in vacuo* using a desiccator for 2 hours and was then moved to an oven for 2h at 200°C. 10 mL anhydrous dioxane was added under a flow of nitrogen to which, 150 mg (0.73067 mmols) of Trimethoxy(3-isocyanotopropyl)silane and 1.607 g (ca 0.8 mmols) of PEG methyl ether (2,000 g/mol) were added, with the reaction mixture stirred for 24h at room temperature, and dried *in vacuo* yielding 1.7221 g, 96 % (Scheme S6). The reactants and the product were analysed by FT-IR (Figure S32), TGA and GPC. TGA of the compound **1** possessed a first derivative at 251.94 °C, and plateaued at ca. 600 °C, with 7.4 % remaining at 900 °C. PEG methyl ether (2,000 g/mol) contained two M_n(GPC) of = 2423 and 667, with the M_n (theoretical) = 2014 (see 4.6 GPC section, Figure S47). GPC showed that compound **1** has three M_n of 5073, 2374 and 674, M_n(theoretical) = 2234 (see 4.6 GPC section, Figure S48). The major peak of the M_n(GPC) for PEG methyl ether (2,000 g/mol) was slightly smaller than that of compound **1**, which is expected suggesting that the reaction took place. The disappearance of the isocyanate peak in the FT-IR spectrum at 2265 cm^{-1} and the presence of 1663 cm^{-1} and 1556 cm^{-1}, which indicate O-C and C=O respectively and would likewise suggest that the reaction happened. Preliminary experiments have been carried out using NMR on compound **1**. Without suppression, the CH_2 groups of the polyethylene glycol chain it was not possible to observe the other peaks within this polymer (see 4.4 NMR section Figure S43). With suppression, however in the region of CH_2 groups of the polyethylene glycol chain, it was possible to presence of shifts at δ = *0.49 (*Si$\boldsymbol{CH_2}$$CH_2$$CH_2$, 2H, m), 1.47 (Si$CH_2$$\boldsymbol{CH_2}$$CH_2$, 2H, m) (see NMR section Figure S44).

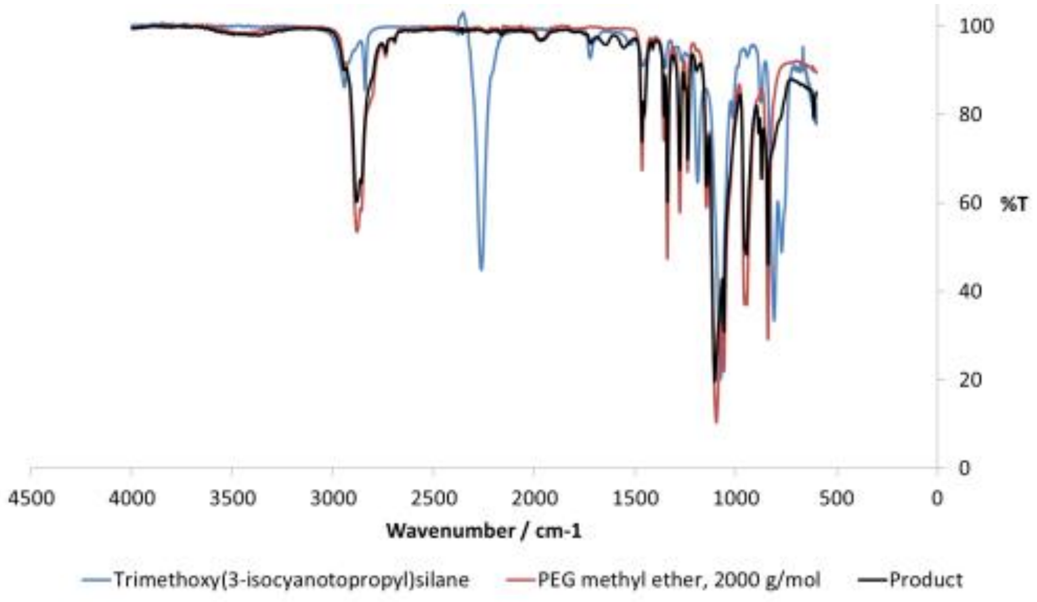

Figure S32. FTIR spectra of the product of the reaction (IR(neat): 2946, 2884, 2860, 2741, 2695, 1721, 1647, 1556, 1467, 1455, 1413, 1360, 1340, 1279, 1254, 1240, 1195, 1146, 1106, 1060, 947, 889, 873, 842, 614 cm^{-1}) compared to the starting materials Trimethoxy(3-isocyanotopropyl)silane IR (neat): 2945, 2843, 2265, 1725, 1693, 1525, 1462, 1414, 1350, 1310, 1270, 1191, 1153, 1077, 1016, 990, 947, 879, 811, 774 cm^{-1} and PEG methyl ether (2,000 g/mol) (IR(neat): 2946, 2883, 2861, 2741, 2695, 1970, 1467, 1455, 1413, 1360, 1341, 1279, 1241, 1196, 1147, 1098, 1060, 957, 947, 841, 623, 615 cm^{-1}).

Reaction B: The same protocol was followed for the synthesis of compounds **Type II** using the 3 kDa and 5 kDa PEGs. A Schlenk was pre-silylated using dimethyldichloride silane *in vacuo* using a desiccator for 2 hours and was then moved to an oven for 2h at 200°C. 20 mL anhydrous dioxane was added under a flow of nitrogen to which, 15 mg (0.073 mmols) of Trimethoxy(3-isocyanotopropyl)silane and 240.7 mg (ca. 0.08 mmols) (NH_2-PEG-COOH)HCl (3,000 g/mol) and 3 equivalents of trietheylamine (22.6 mg) were added, with the reaction mixture stirred for 24h at room temperature. The reactants and the product (yielding 231 mg) were analysed by FT-IR (Figure S33), GPC and TGA. The TGA of compound **2** (3 kDa) possessed first derivatives at 340 ºC, 371.86 ºC, 390.5 ºC and plateaued at 600 °C, with 10.5 % remaining at 900 ºC (see 4.3 TGA analyses section, Table S4). This was significantly different from the starting materials, trimethoxy(3-isocyanotopropyl)silane (first derivative 157.26 ºC, plateau 400 ºC, 1.6% remaining at 900 ºC) and NH_2-PEG-COOH, 3 kDa (first derivative 251.94 ºC, plateau 600 ºC, 9.2% remaining at 900 ºC) (see TGA analyses section, Table S4).

Starting reagent, NH_2-PEG-COOH, 3 kDa possessed two peaks, with a M_n(GPC) = 3234, which compares well to the M_n(theoretical) = 3000, and a second at 660 (see 4.6 GPC section, Figure S49). Compound **2** (3 kDa) possessed a single M_n(GPC) = 3180, with a M_n(theoretical) = 3325 (see 4.6 GPC section,, Figure S50). Likewise NH_2-PEG-COOH, 5 kDa contained two peaks, with the major at M_n(GPC) = 4148 and 671 and the M_n(theoretical) = 5006 (see 4.6 GPC section, Figure S51). Compound **2** (5 kDa) possessed two M_n(GPC) of 4302, 685, with a M_n(theoretical) = 5331 (see GPC section, Figure S52). Two peaks in the starting material may indicate that decomposition is occurring, despite storage at -20°C.

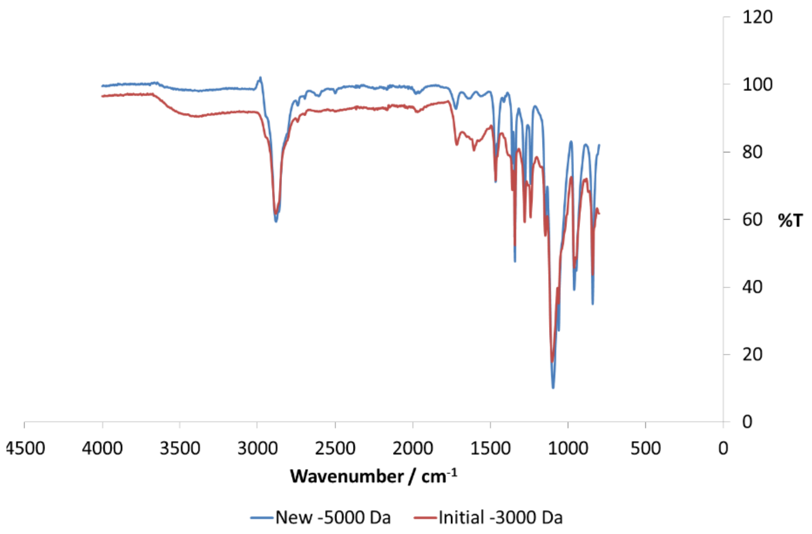

Figure S33. FT-IR spectra of trimethoxy(3-isocyanotopropyl)silane and product of reaction B: Compound **2** (3 kDa) IR(neat): 3426, 2884, 2743, 2696, 1717, 1653, 1606, 1575, 1467, 1360, 1342, 1280, 1241, 1147, 1103, 1061, 961, 870, 841, 801, 771, 746, 731, 696 cm^{-1}. Compound **2** (5 kDa) IR(neat): 2944, 2881, 2863, 2741, 2696, 2606, 2500, 1980, 1722, 1640, 1559, 1467, 1456, 1360, 1342, 1279, 1241, 1146, 1096, 1060, 961, 947, 841 cm^{-1}.

Reaction C: A Schlenk was pre-silylated using dimethyldichloride silane *in vacuo* using a desiccator for 2 hours and was then moved to an oven for 2h at 200°C. 10 mL dry, THF and 10 µL aqueous ammonia (34%) was added followed by 99.6 mg Eu:YVO_4 nanophosphor under a flow of nitrogen and stirred for 1h. 850.9 mg of compound **1** and 150.5 mg of compound **2** (3 kDa) were added under a flow of nitrogen and the reaction mixture was stirred overnight at room temperature. Subsequently this it was centrifuged at 3000 rpm for 5 minutes and washed 3x with dry THF. The solid and supernatant were dialysed yielding 31.8 mg and 647.9 respectively and analysed by FT-IR (Figure S34) and TGA. Both solid and supernatant from this reaction were analysed by TGA. The solid displayed first derivatives at 230.81 °C and spike at 352.97 °C, whereas the supernatant possessed first derivatives at 247.3 ºC and 364.56 ºC. TGA investigations on the solid showed a plateau at ca. 450 ºC (see TGA analyses section, in Table S4).

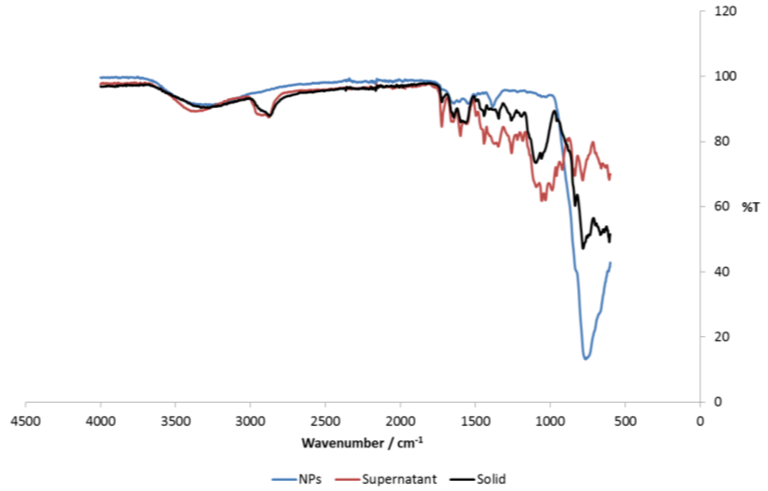

Figure S34. IR spectra of uncoated nanoparticles (IR(neat): 3289, 1724, 1643, 1608, 1551, 1385, 763, 675 cm^{-1}) and the supernatant (IR(neat): 3375, 2951, 2927, 2877, 1724, 1648, 1599, 1560, 1496, 1442, 1403, 1347, 1258, 1220, 1184, 1094, 1058, 1032, 988, 958, 920, 881, 836, 784, 753, 701, 661, 633, 607 cm^{-1}) and of the solid (IR(neat): 3278, 2871, 1724, 1643, 1563, 1442, 1344, 1260, 1191, 1097, 1059, 957, 834, 782, 752, 737, 700, 667, 639, 607 cm^{-1})

Thermogravimetric analysis

Thermogravimetric analysis is a technique whereby the mass of a sample is measured according to an increase in temperature. This approach was designed whereby a decrease in mass of coated nanophosphor can be correlated

to the mass of the coated material. If the molecular weight of the coating material is known, the number of moles required to coat a given mass of nanoparticle can therefore be calculated.

The mass of the coating material can be calculated by:

$$\%_C = 100\% - [\%_R \div 100]$$

Where $\%_C$ is the estimated percentage mass of coating material and $\%_R$ is the remaining percentage mass of sample

Table S4. Table of TGA data including the first derivative peaks, point of plateau and the percentage mass remaining at 900 °C

Sample	First Derivative Peak(s) / °C	Point of Plateau	% at 900 °C
Bulk powder particulate $Y_{0.9}Eu_{0.1}VO_4$	N/A	Not clear	96.1
Trimethoxy(3-isocyanotopropyl)silane	157.26	400 °C, 2.2%	1.6
NH_2-PEG-COOH (3 kDa)	259.32, 320.26	550 °C, 7.8%	9.2
Compound **5** (3 kDa)	340 (s), 371.86, 390.5 (s)	600 °C, 12%	10.5
NH_2-PEG-COOH (5 kDa)	247.74, 313.54	500 °C, 6.80	6.33
**Solid fraction of coated nanoparticle with compound 1 (3 kDa)**	230.81 and spike at 352.97	450 °C, 73%	71.0
**Supernatant fraction of coated nanoparticle with compound 1 (3 kDa)**	247.3, 364.56	ca. 700 °C, 9%	8.0
NH_2-PEG-COOH (5 kDa)	247.74, 313.54	500 °C, 6.80	6.33

To calculate the number of moles of coating material required, coated nanoparticle with compound **1** (3 kDa). Using the equation above gives 26.12% and 91.68% for the solid and supernatant fractions of coated nanoparticle with compound **1** (3 kDa) respectively.

The supernatant fraction was integrated using ACD labs software, whereby integration of the first derivatives indicates that the peak at 364.56 °C is 22% mass, representing SiO_2-PEG-COOH (Mr ≈ 3250 Da). Correspondingly the peak at 247.3 °C is 70% mass, representing SiO_2-PEG-Me ether (Mr ≈ 2250 Da). Considering that 91.68% corresponds to coating material these numbers can be more accurately calculated as 21.92% and 69.76% for SiO_2-PEG-COOH and SiO_2-PEG-Me ether respectively.

Therefore, for 100 mg of NP (representing 8.32% of coated material), total mass required is 1201.25 mg. SiO_2-PEG-COOH (21.92%) would be 263.3 mg, 0.0810 m moles and SiO_2-PEG-Me ether 69.76% would be 838.0 mg, 0.372 m moles. The total number of moles required is thus 0.453 m moles siloxane per 100 mg of NP. This therefore could be used for EDC coupling reactions and for using only the required number of moles of coating agent. Interestingly, the calculated molar ratio is 1:4.6, interestingly the ratio added was 1:8.17.

Probing the stability of the luminescence with time in aqueous media

The luminescence of coated and uncoated bulk powder particulate $Y_{0.9}Eu_{0.1}VO_4$ nanophosphors, 0.1 mg/mL in Milli-Q water was tested using excitation at 290 nm (Figures S10, S35-S37). This was carried out in glass vials and in Eppendorfs tubes to assess the suitability of these coated materials under biological testing conditions, whereby

plastic Eppendorf tubes were used as standard. These were also assessed at room temperature and at 4 °C. Uncoated materials were investigated as a control at the same w/v concentration (Figure S10 and S32). A luminescence emission was detectable up to 4 weeks under all conditions, with data gathered comparable at room temperature and at 4 °C as it was in glass vials and in plastic Eppendorf tubes. The luminescence of these samples was monitored over the two weeks.

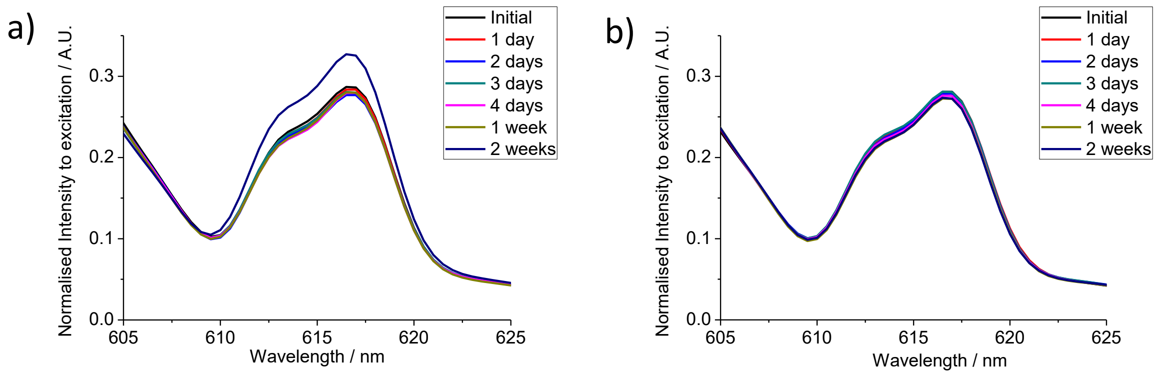

Figure S35. Luminescence spectra of uncoated bulk powder particulate $Y_{0.9}Eu_{0.1}VO_4$ nanomaterials (λ_{ex} = 290 nm) dispersed in Milli-Q water 0.1 mg/mL stored in an Eppendorf tube, protected from light a) at room temperature and b) at 4 °C up to 2 weeks.

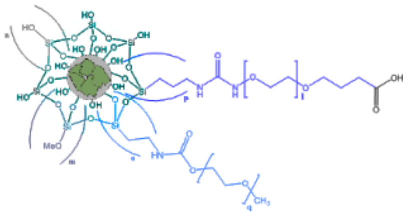

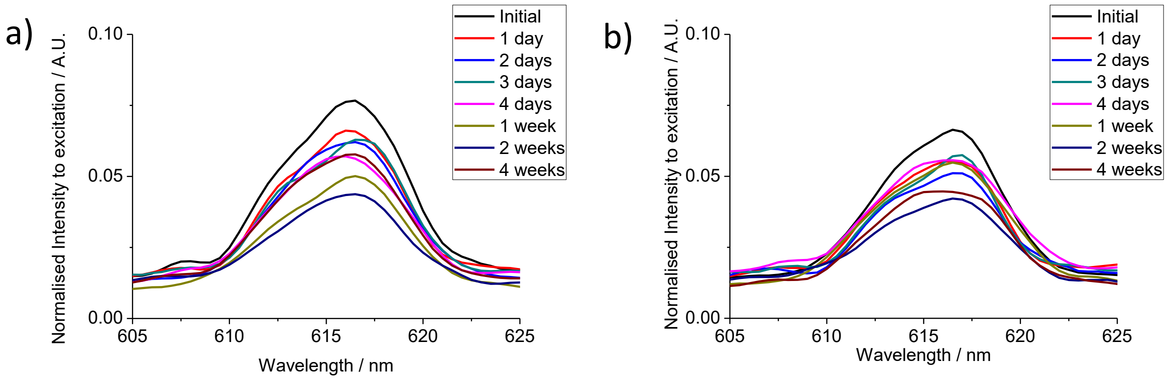

Figure S36. Luminescence spectra of PEG-coated bulk powder particulate $Y_{0.9}Eu_{0.1}VO_4$ nanomaterials (λ_{ex} = 290 nm) in Milli-Q water 0.1 mg/mL stored in a glass vial, protected from light a) at room temperature and b) at 4 °C up to 4 weeks. In this typical example, l=60 and q = 40.

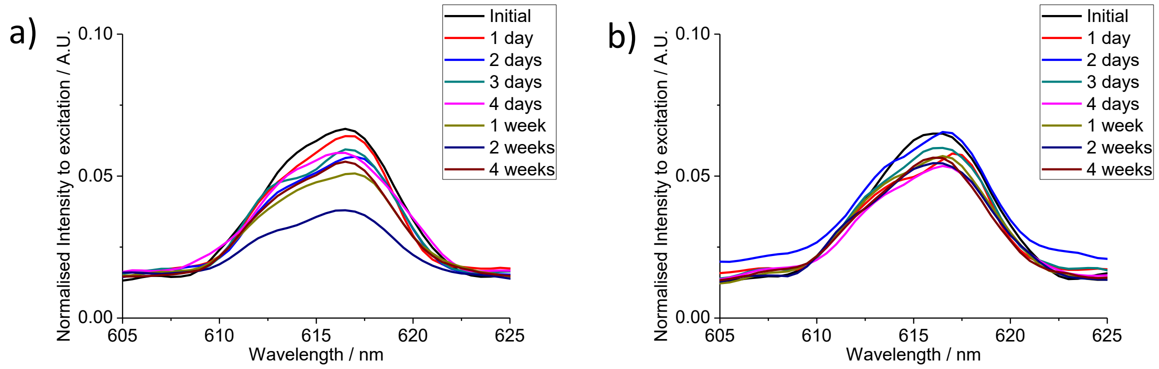

Figure S37. Luminescence spectra of PEG-coated bulk powder particulate $Y_{0.9}Eu_{0.1}VO_4$ nanomaterials (λ_{ex} = 290 nm) in Milli-Q water 0.1 mg/mL stored in an Eppendorf tube, protected from light a) at room temperature and b) at 4 °C for 4 weeks. In this typical example, l=60 and q = 40.

TGA analyses

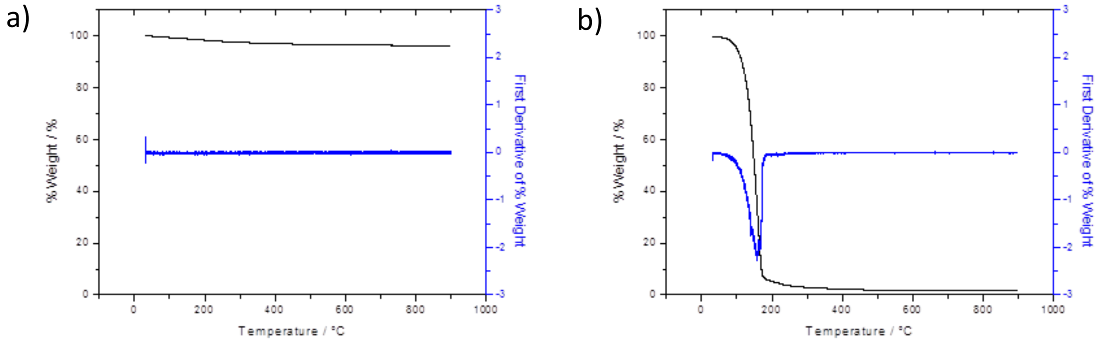

Figure S38. TGA of a) bulk powder particulate $Y_{0.9}Eu_{0.1}VO_4$, solid bulk material powder, b) Trimethoxy(3-isocyanotopropyl)silane

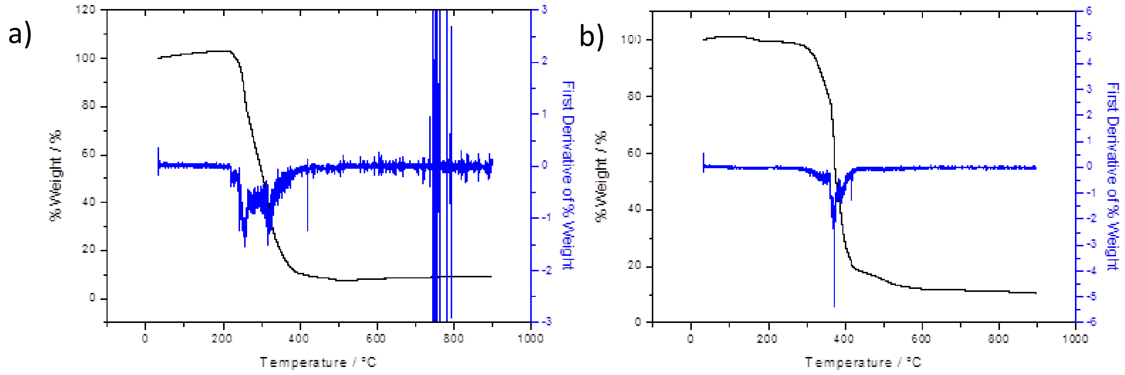

Figure S39. TGA of a) aminooxy NH_2-PEG-COOH (3 kDa), b) coated bulk powder particulate $Y_{0.9}Eu_{0.1}VO_4$ nanophosphors with compound **2** (3 kDa)

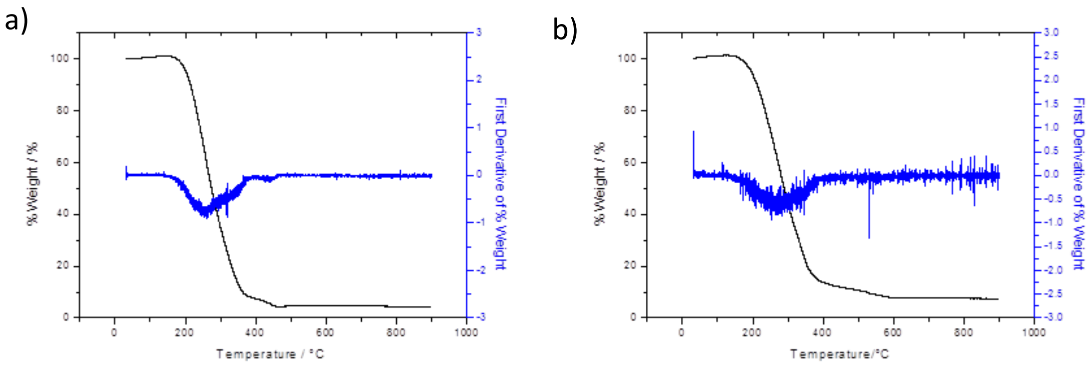

Figure S40. TGA of a) PEG-Me ether, b) Compound **Type I**

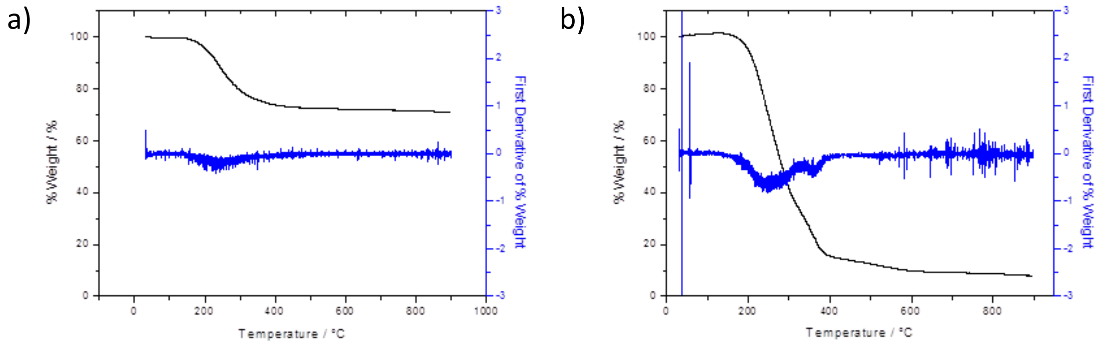

Figure S41. TGA of a) Solid fraction of coated bulk powder particulate $Y_{0.9}Eu_{0.1}VO_4$ with compound **Type I** (3 kDa), b) Supernatant fraction of coated nanoparticle with compound **I** (3 kDa)

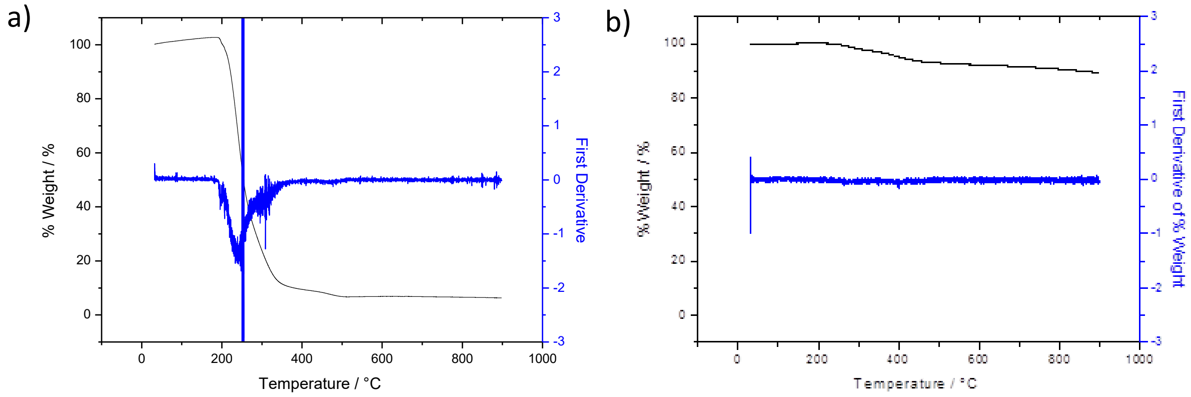

Figure S42. TGA of a) aminooxy NH_2-PEG-COOH (5 kDa), b) Coated bulk powder particulate $Y_{0.9}Eu_{0.1}VO_4$

NMR spectroscopy for model linkers studies

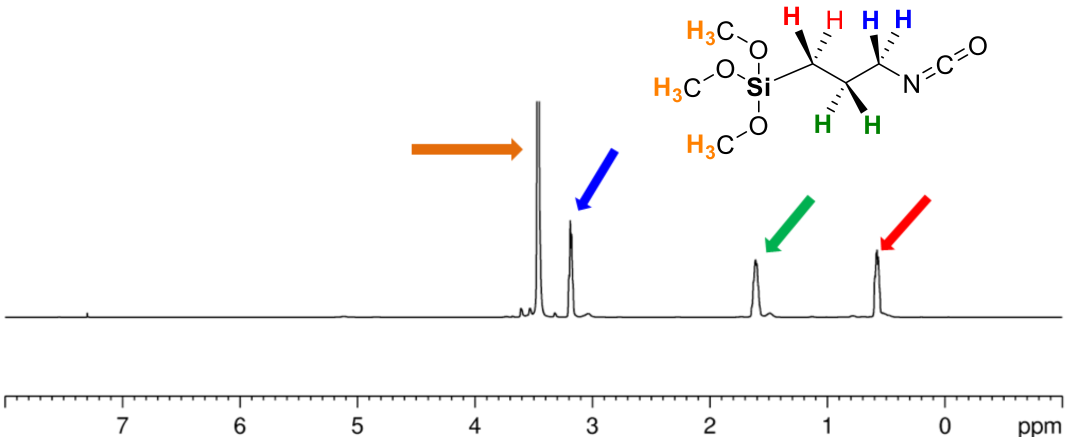

Figure S43. ^{1}H NMR 500 MHz spectrum of Trimethoxy(3-isocyanotopropyl)silane

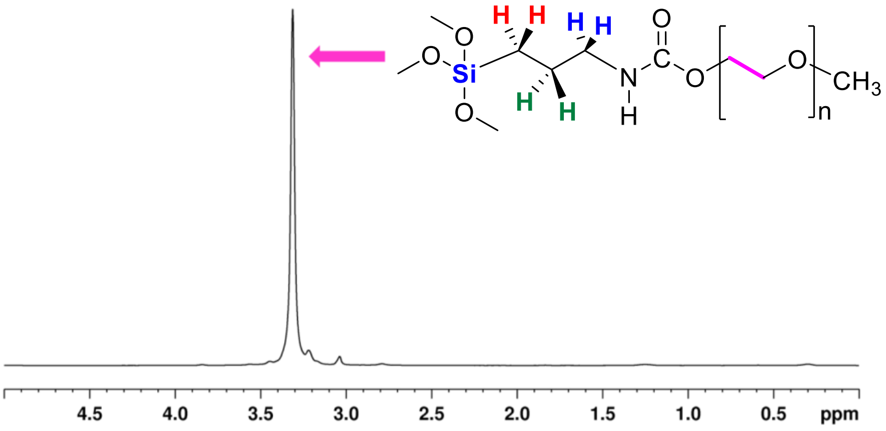

Figure S44. ^{1}H NMR 500 MHz spectrum of compound **Type I**

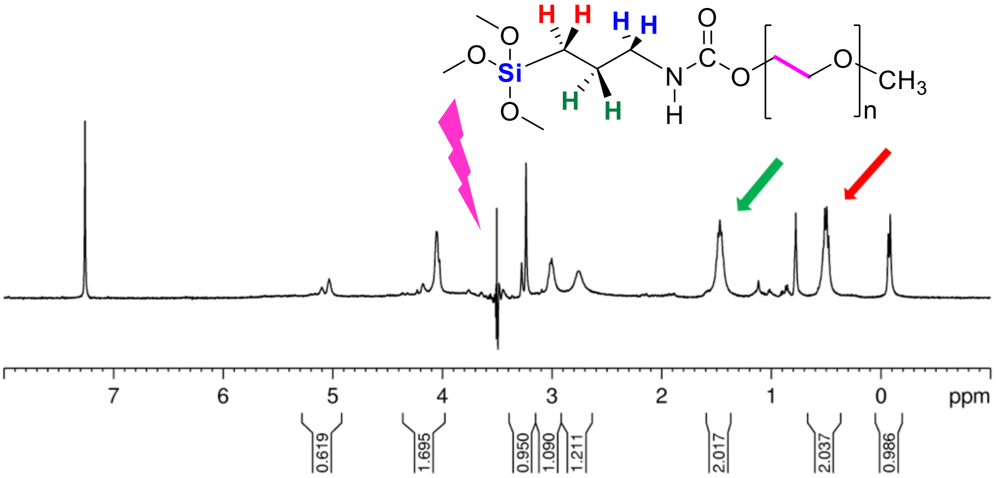

Figure S45. ^{1}H NMR 500 MHz spectrum of compound **Type I**, with suppression in the region of CH_2 groups of the polyethylene glycol chain, showing presence of shifts at *δ = 0.49 (CH₂*CH₂CH₂, 2H, m), 1.47 (CH₂*CH₂*CH₂, 2H, m), highlighted in red and green respectively.

Raman Spectroscopy:

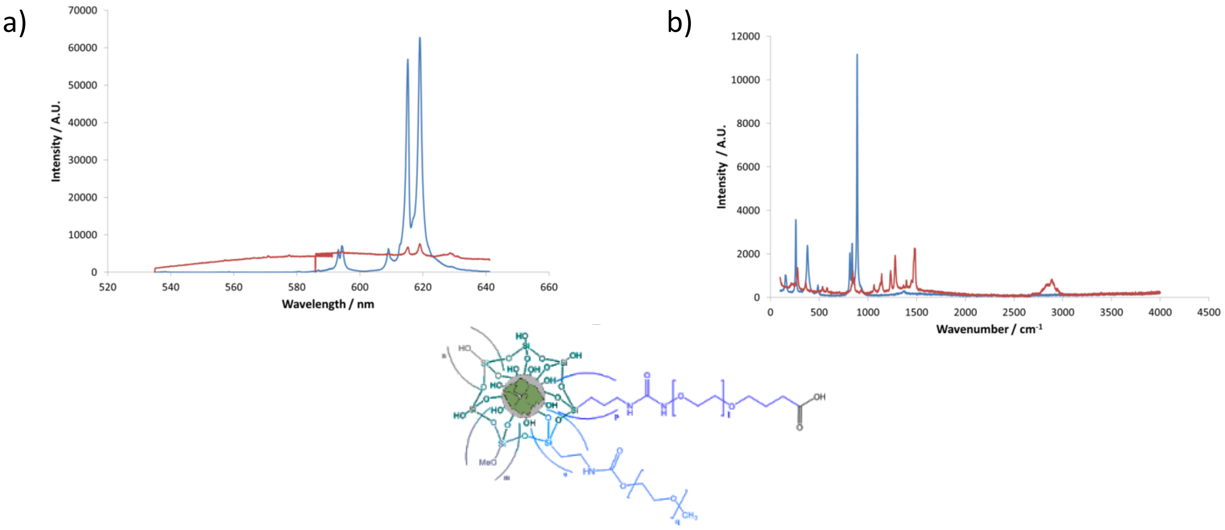

Figure S46. Raman investigations of functionalised NPs resulting from the method optimised by coating NPs via pre-reacting to silane and capping with Me-PEG ester, where the PEG used had Mw ca. 5000 g/mol, l=q=100. Blue line: coated NPs; red line H_2N-PEG-COOH; a) green laser 532 nm and b) red laser, 785 nm

GPC

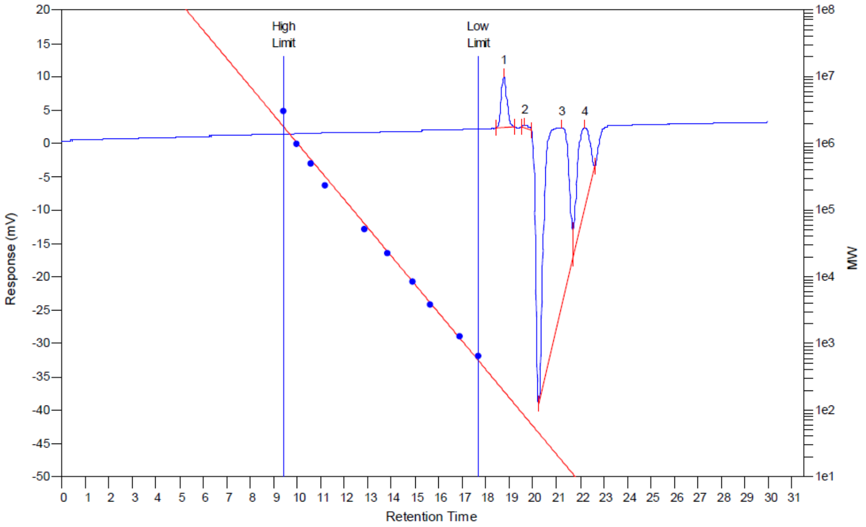

Figure S47. GPC of THF blank.

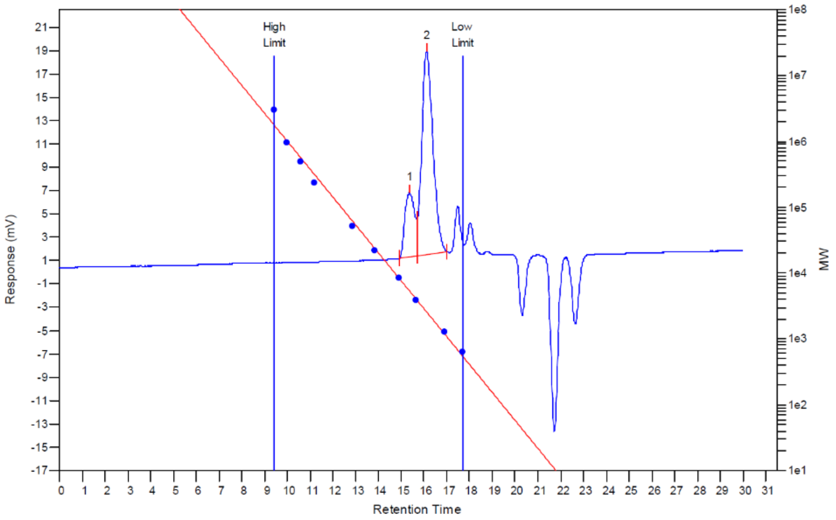

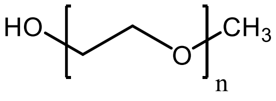

2000 Daltons n = 45

Chemical Formula: $C_{91}H_{184}O_{46}$
Molecular Weight: 2014.427

Figure S48. GPC of PEG methyl ether, M_n(GPC of 1) = 5208 and M_n(GPC of 2) = 2366, M_n (theoretical) = 2014.

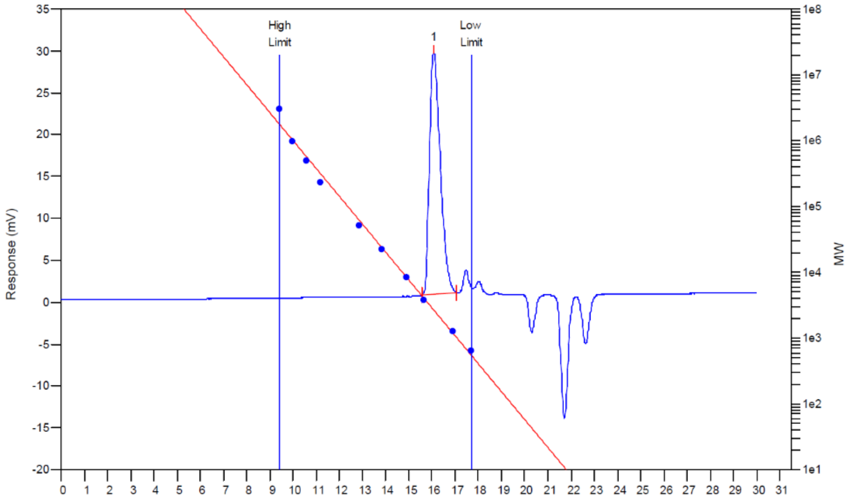

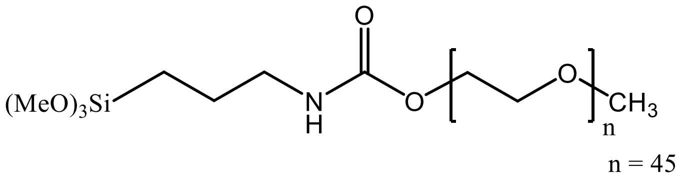

Chemical Formula: $C_{99}H_{202}NO_{50}Si$
Molecular Weight: 2234.747

Figure S49. GPC of Compound **Type I** , M_n(GPC of 1) = 2387, M_n (theoretical) = 2235.

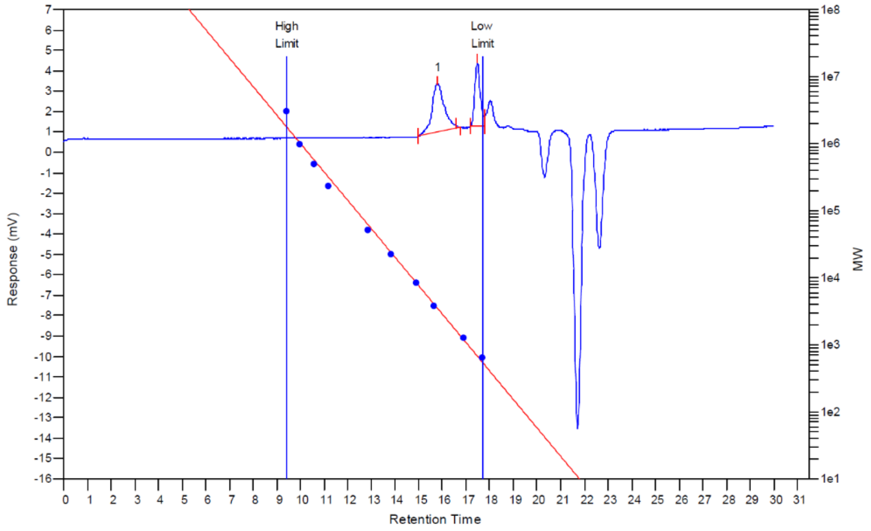

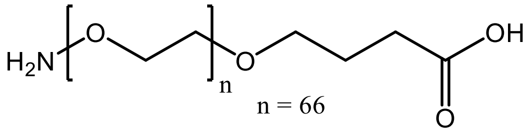

Chemical Formula: $C_{134}H_{271}NO_{69}$
Molecular Weight: 3000.580

Figure S50. GPC of NH_2-PEG-COOH (3 kDa) M_N(GPC of 1) = 3234 and M_N(GPC of 2) = 660, M_N (theoretical) = 3001.

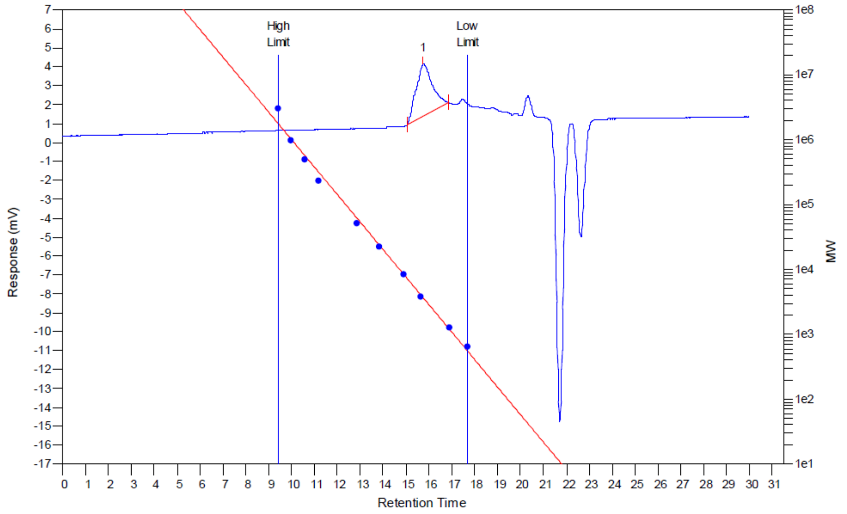

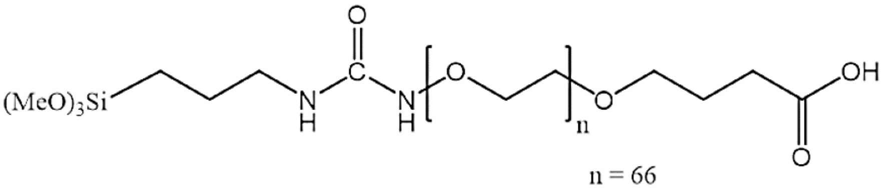

Chemical Formula: $C_{145}H_{295}N_3O_{76}Si$
Molecular Weight: 3324.985

Figure S51. GPC of Compound Type **II** (3 kDa), M_n(GPC) = 3180, M_n (theoretical) = 3325.

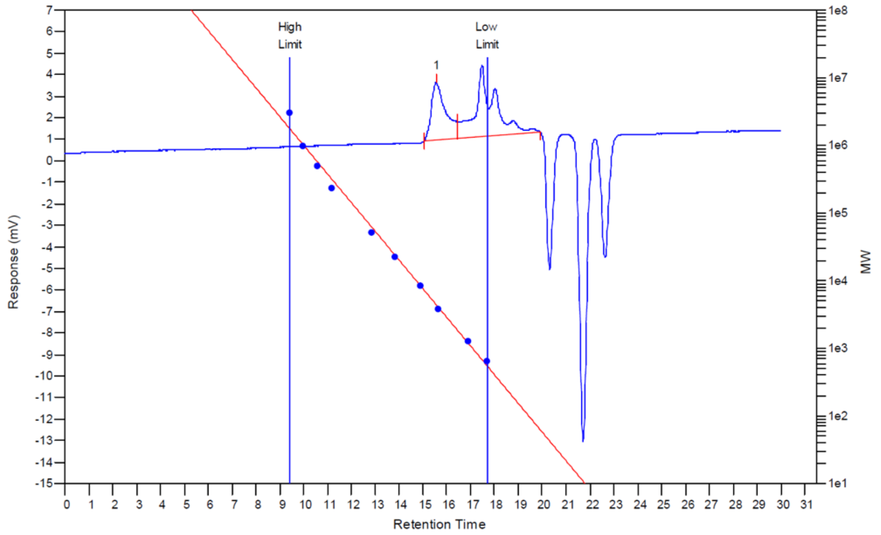

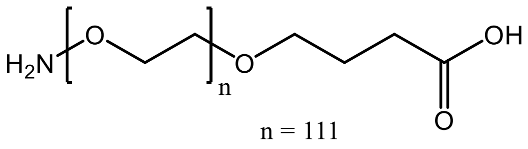

Chemical Formula: $C_{226}H_{451}NO_{114}$
Molecular Weight: 5006.987

Figure S52. GPC of NH_2-PEG-COOH (5 kDa) M_N(GPC OF 1) = 3544, M_n (theoretical) = 5007.

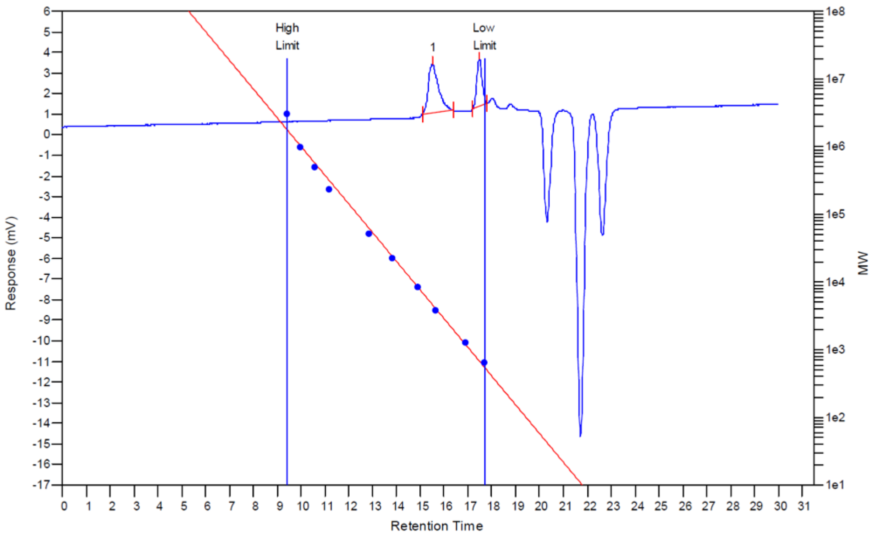

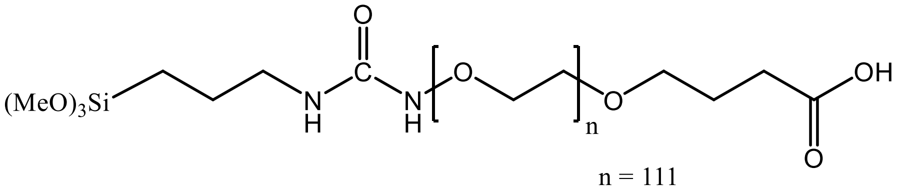

Chemical Formula: $C_{237}H_{475}N_3O_{121}Si$
Molecular Weight: 5331.392

Figure S53. GPC of Compound **Type II** (5 kDa), M_n(GPC of 1) = 4148 and M_n(GPC of 2) = 671, M_n (theoretical) = 5331.

Mass Spectrometry

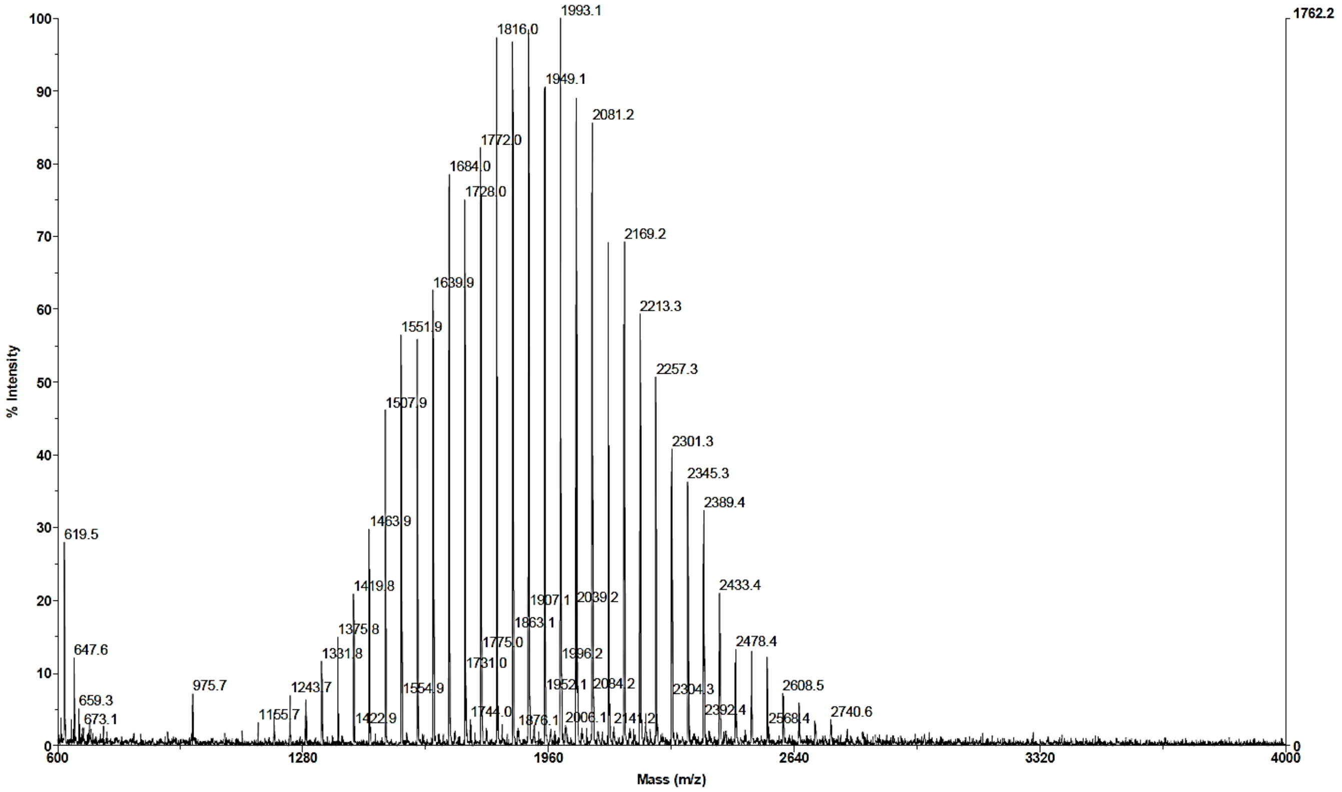

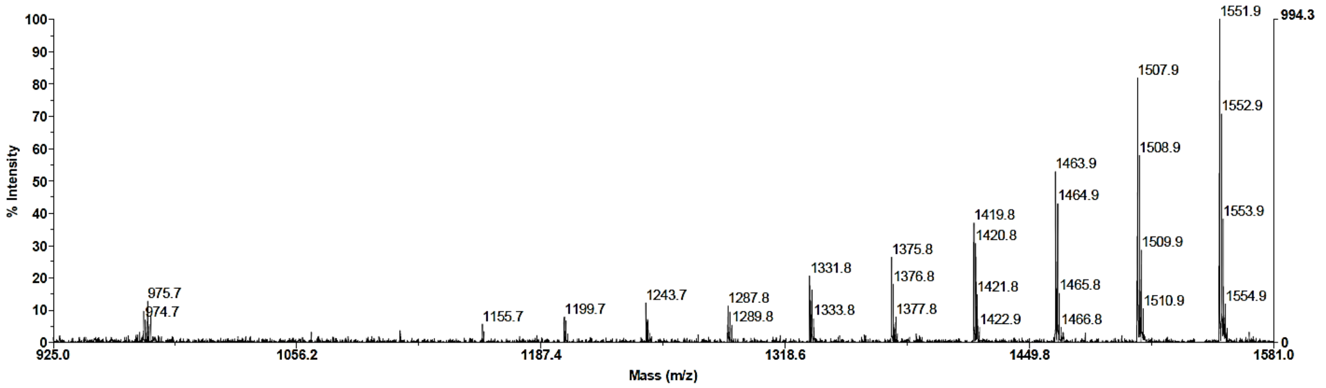

<<BATPAS114-VM-MAP_0001>> Voyager Spec #1=>AdvBC(64,0.5,0.1)=>SM5=>MC[BP = 413.3, 2123]

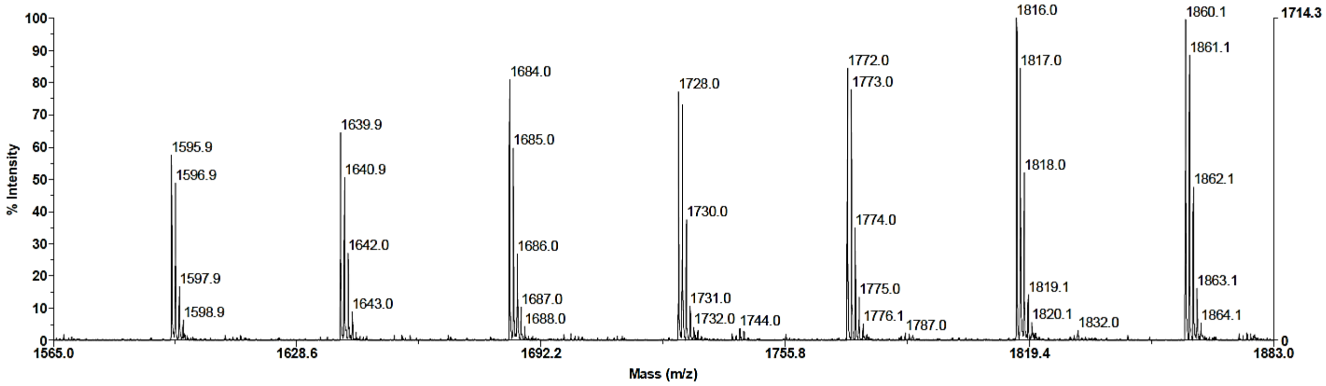

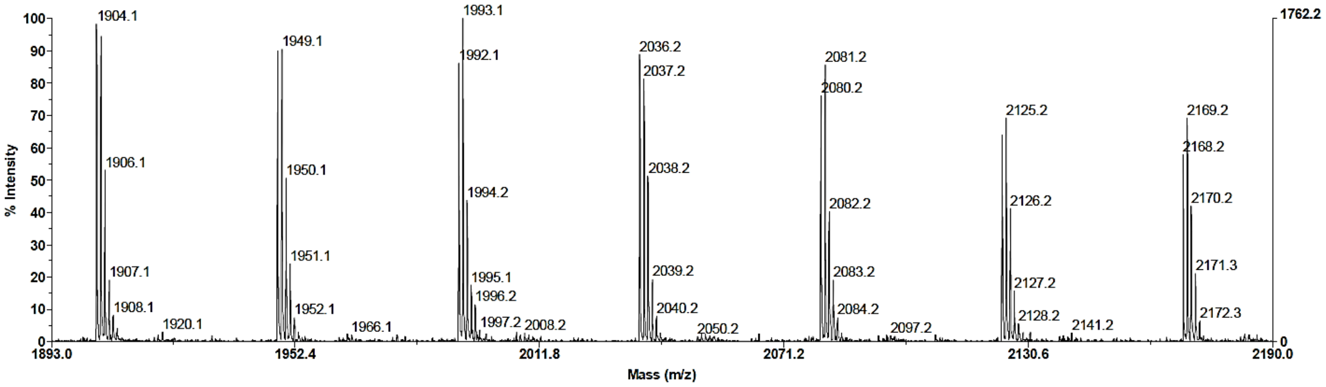

<<BATPAS114-VM-MAP_0001>> Voyager Spec #1=>AdvBC(64,0.5,0.1)=>SM5=>MC[BP = 413.3, 2123]

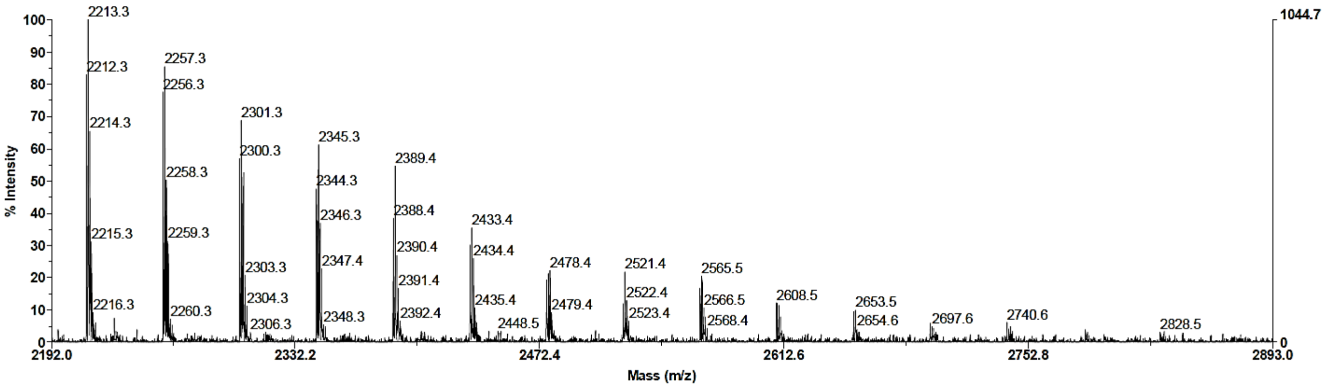

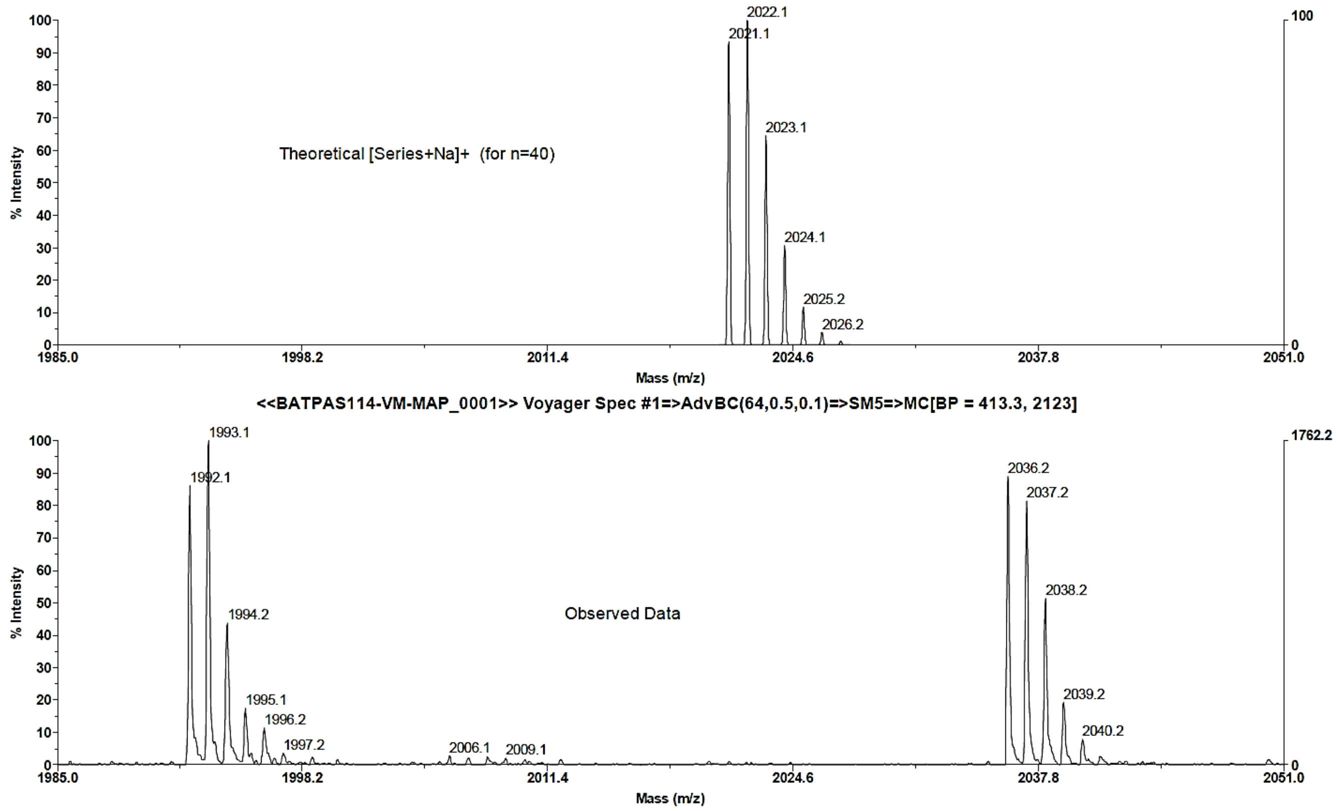

Figure S54. Mass spectra of GPC of Compound 1 (2 kDa). (THF PosRef [1:5] (Dith;THF) +NaOAc)

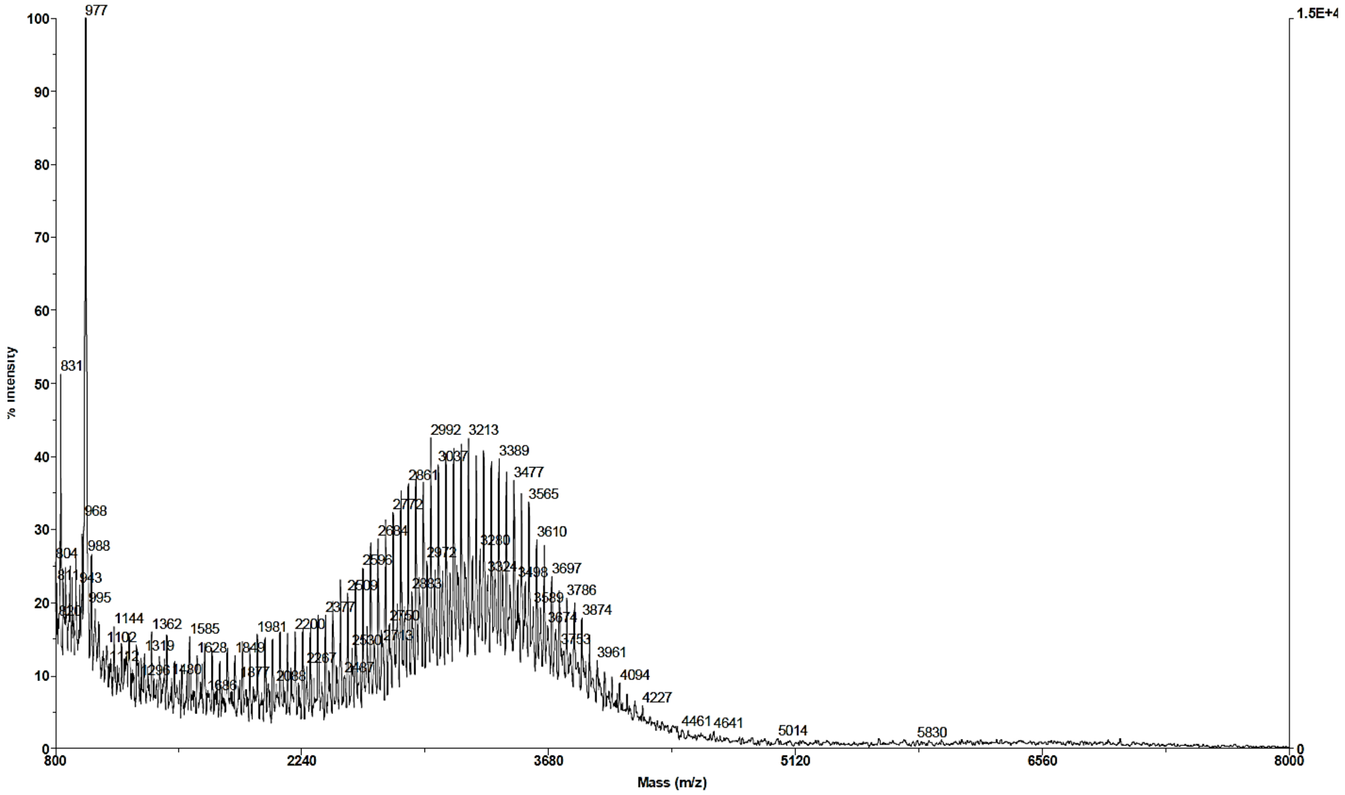

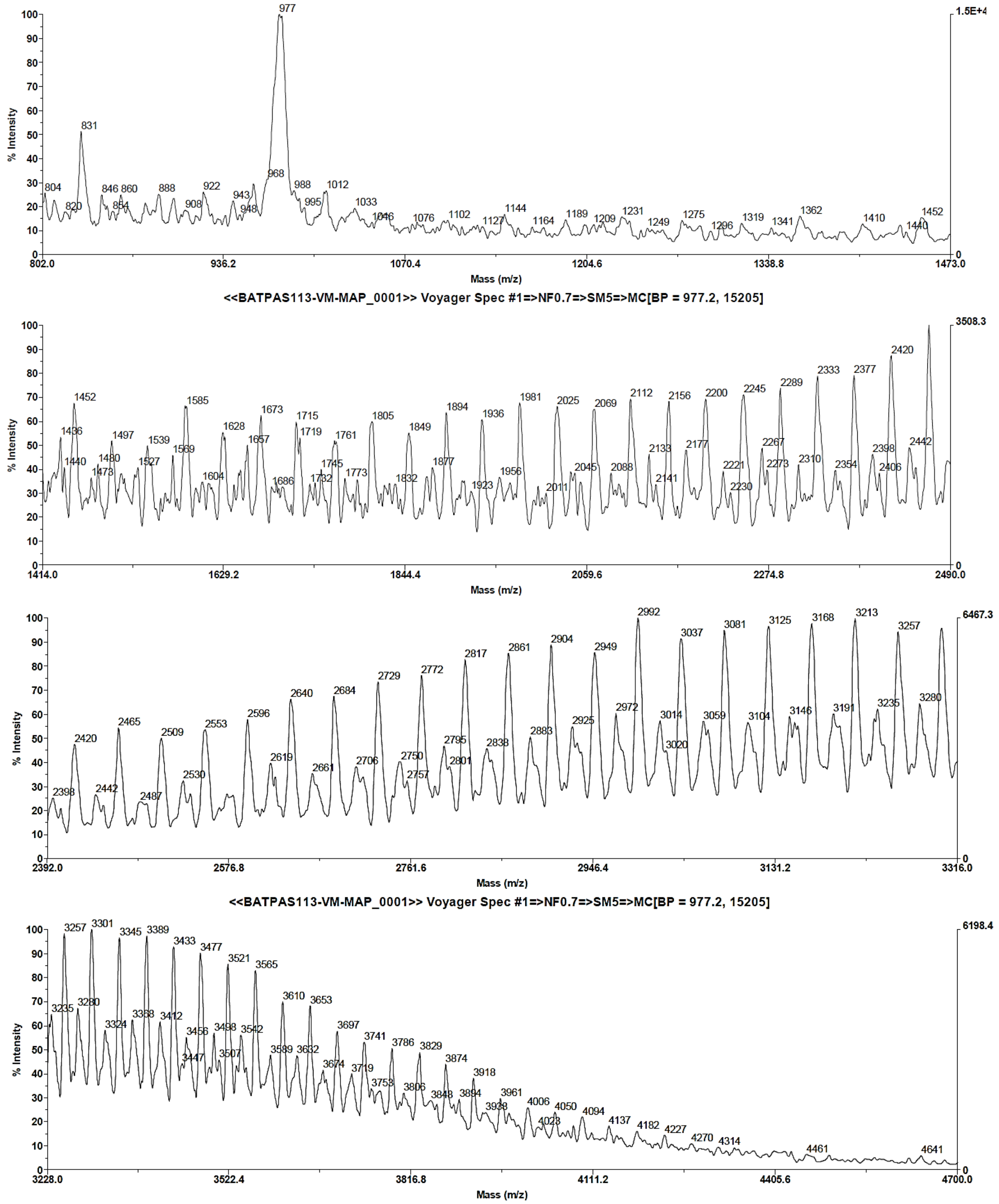

Figure S55. Mass spectra of GPC of Compound II (3 kDa). (THF PosLin [1:5] (Dith;THF) +NaOAc)

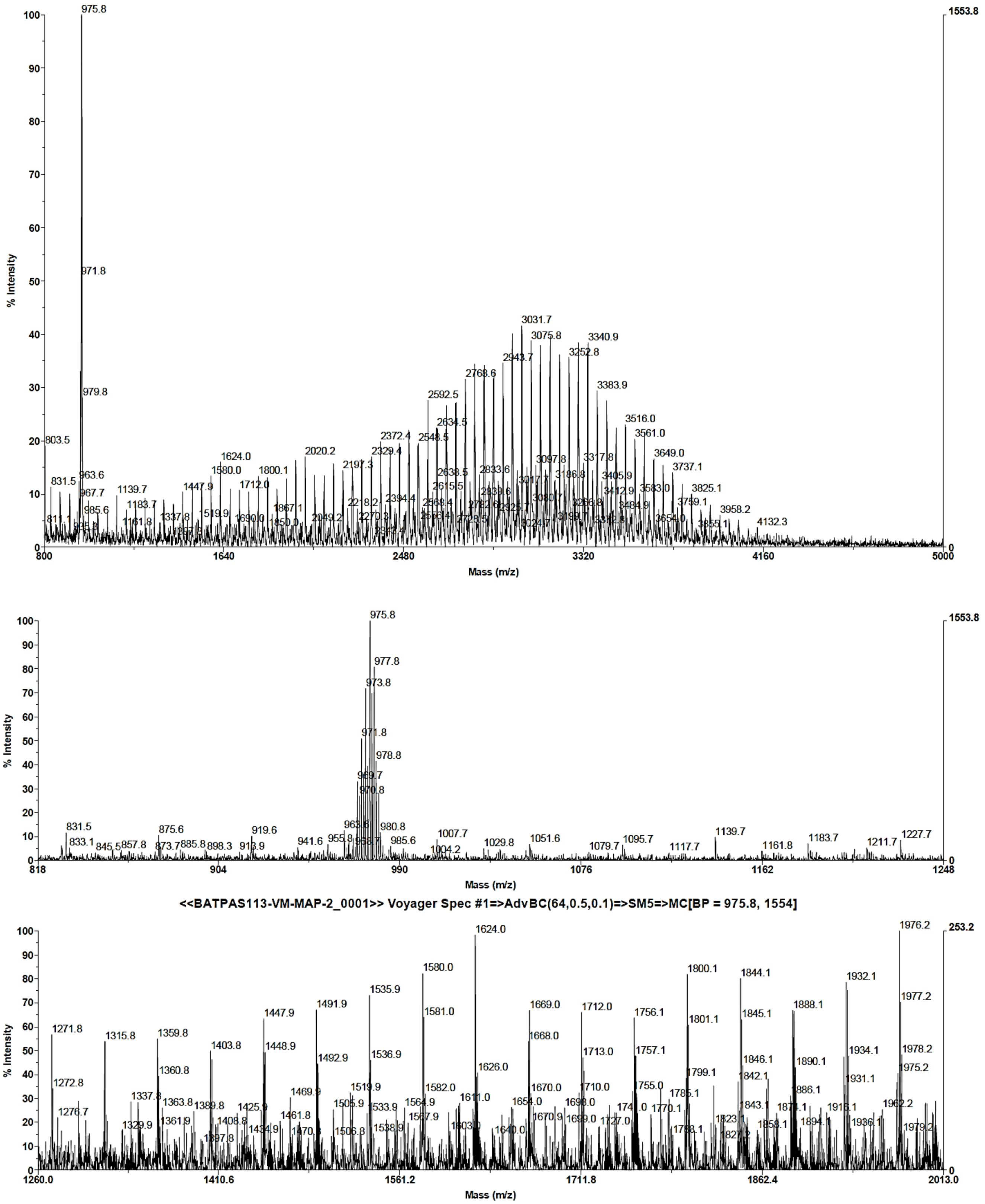
% Intensity
Mass (m/z)
<<BATPAS113-VM-MAP-2_0001>> Voyager Spec #1=>AdvBC(64,0.5,0.1)=>SM5=>MC[BP = 975.8, 1554]

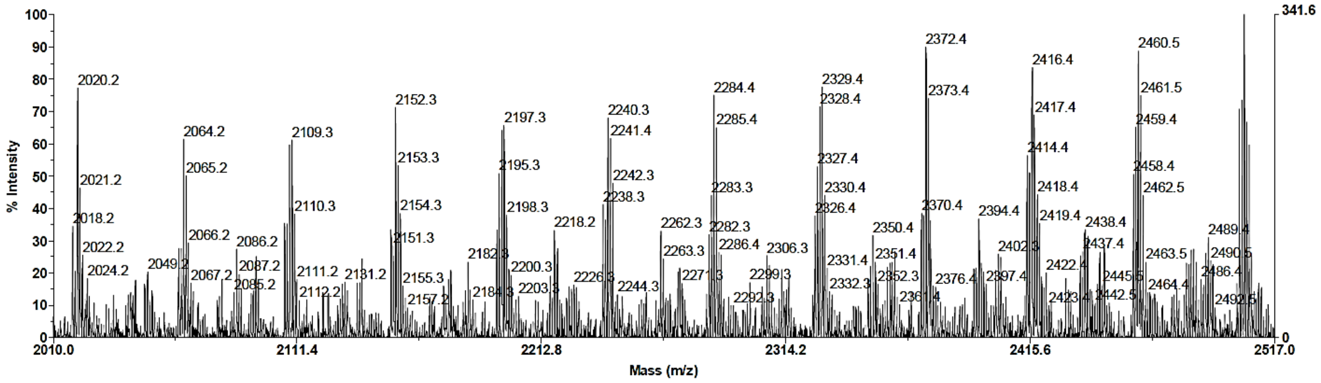

<<BATPAS113-VM-MAP-2_0001>> Voyager Spec #1=>AdvBC(64,0.5,0.1)=>SM5=>MC[BP = 975.8, 1554]

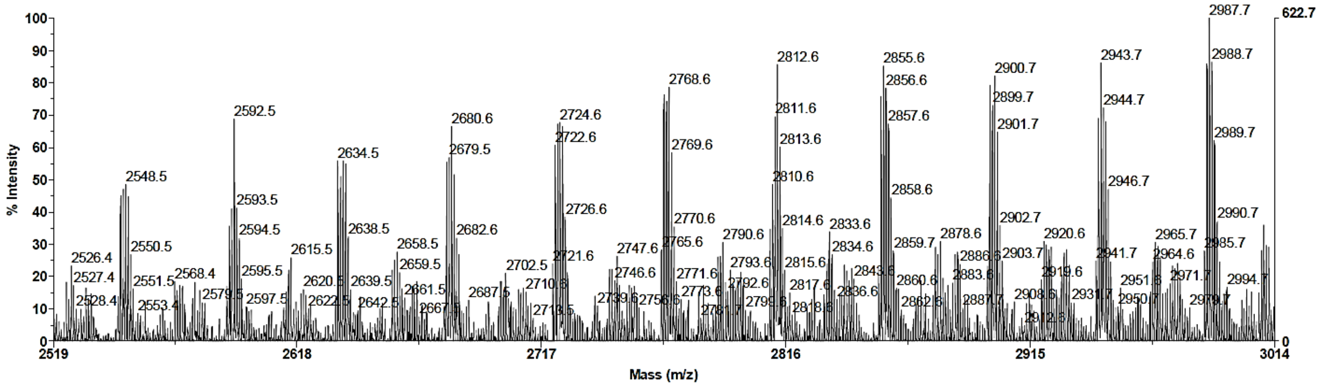

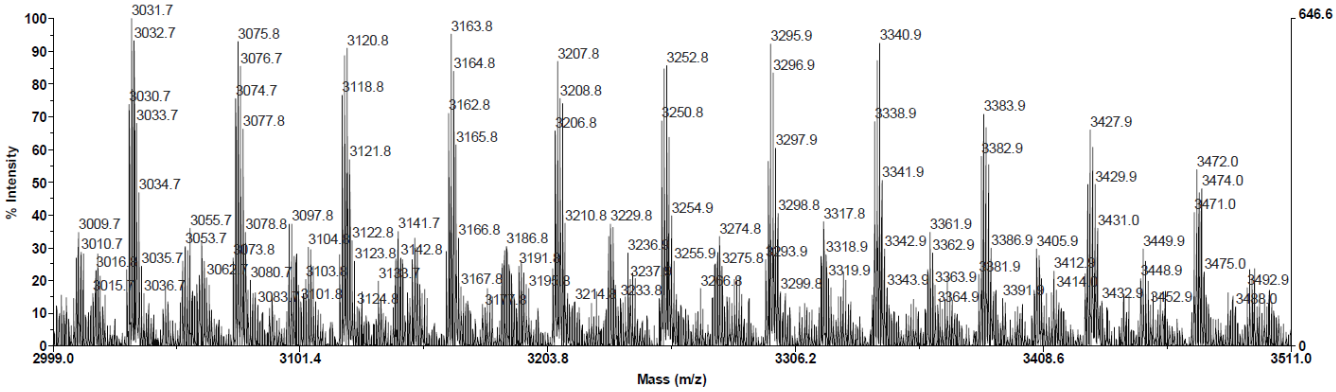

<<BATPAS113-VM-MAP-2_0001>> Voyager Spec #1=>AdvBC(64,0.5,0.1)=>SM5=>MC[BP = 975.8, 1554]

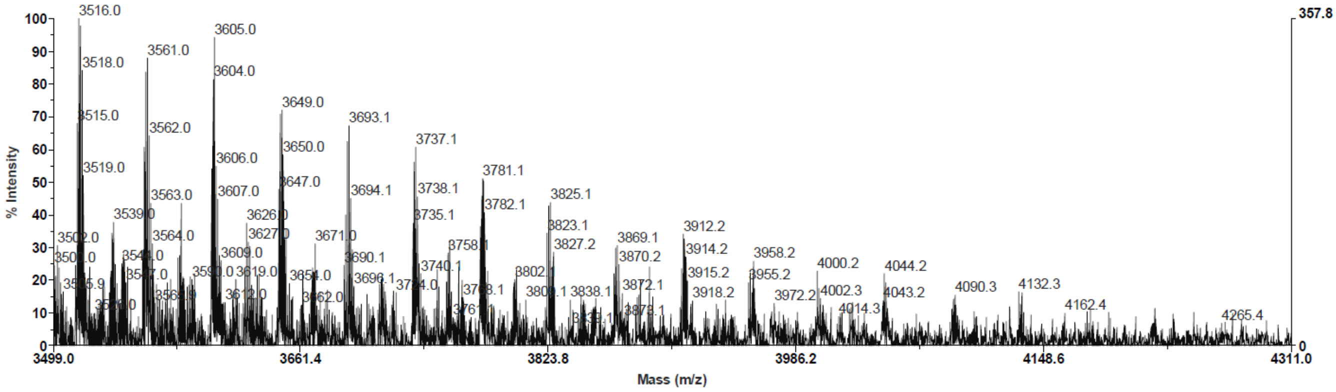

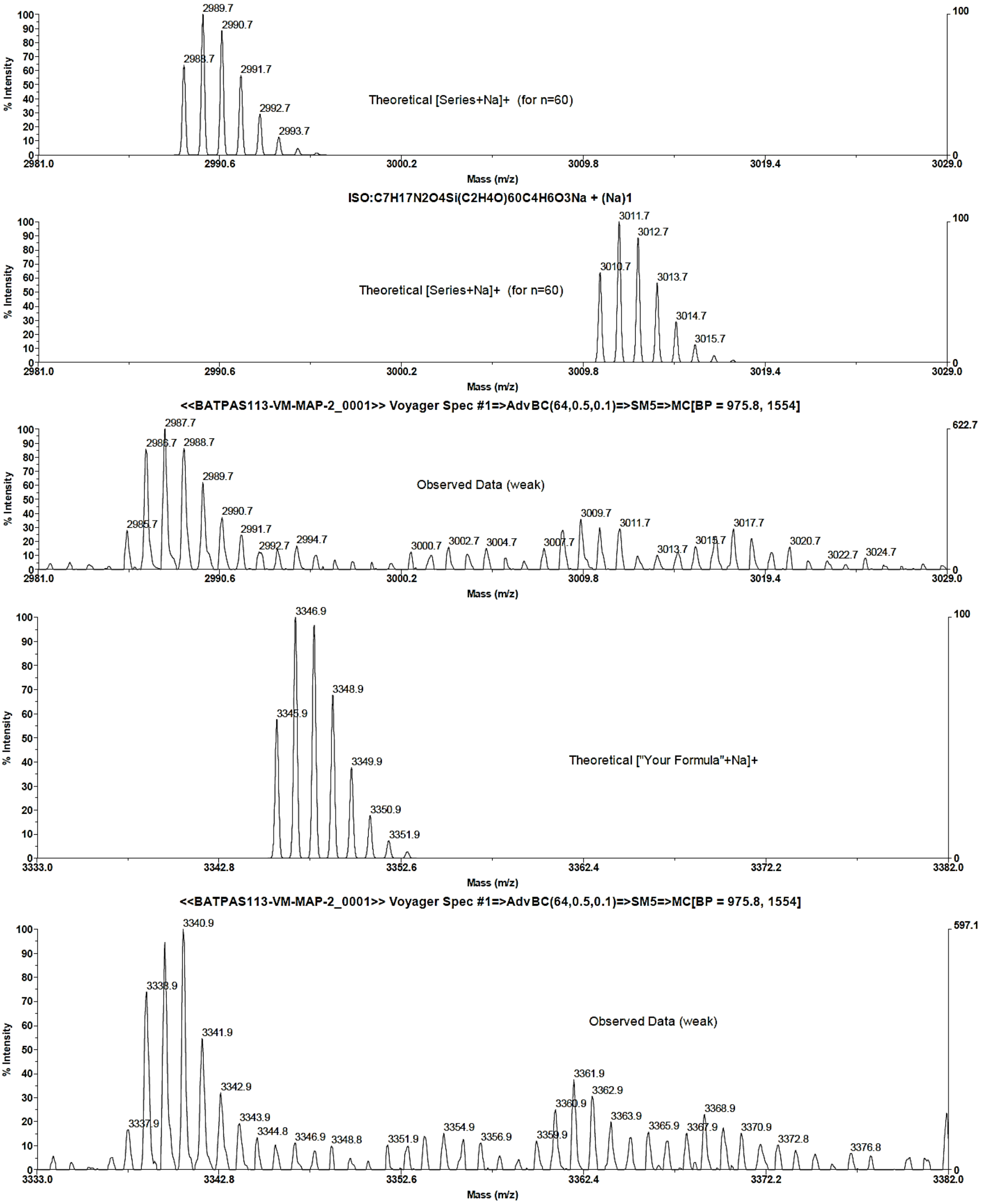

Figure S56. Mass spectra of GPC of Compound Type II (3 kDa). (THF PosRef [1:5] (Dith;THF) +NaOAc)

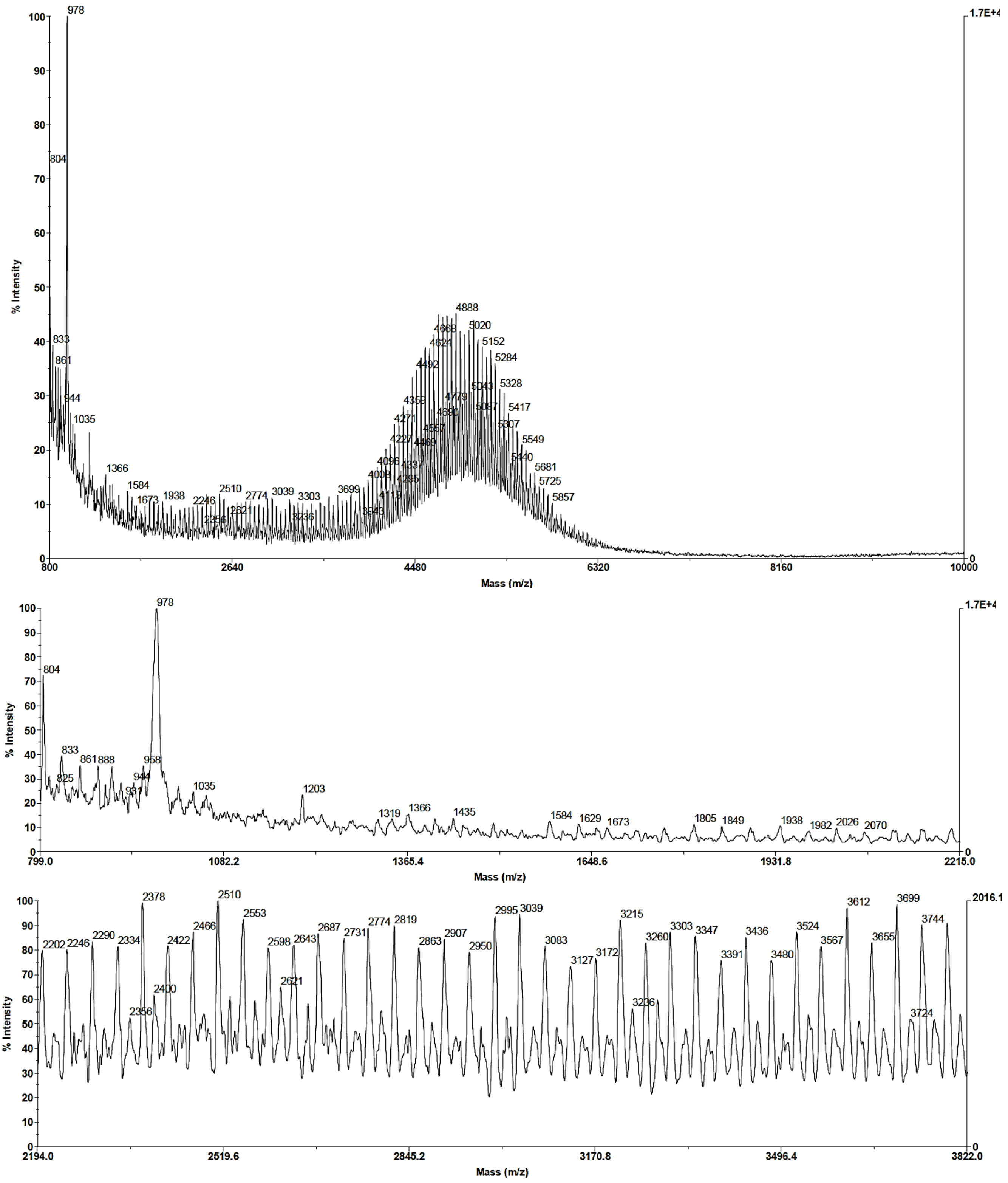
% Intensity
Mass (m/z)
1.7E+4
978
804
833
861
944
1035
1366
1584
1673
1938
2246
2356
2510
2621
2774
3039
3303
3236
3699
3943
4008
4096
4118
4227
4271
4295
4337
4359
4469
4492
4557
4624
4668
4690
4779
4888
5020
5043
5087
5152
5284
5307
5328
5417
5440
5549
5681
5725
5857
800
2640
4480
6320
8160
10000
978
804
833
825
861
888
931
944
958
1035
1203
1319
1366
1435
1584
1629
1673
1805
1849
1938
1982
2026
2070
799.0
1082.2
1365.4
1648.6
1931.8
2215.0
2016.1
2202
2246
2290
2334
2356
2378
2400
2422
2466
2510
2553
2598
2621
2643
2687
2731
2774
2819
2863
2907
2950
2995
3039
3083
3127
3172
3215
3236
3260
3303
3347
3391
3436
3480
3524
3567
3612
3655
3699
3724
3744
2194.0
2519.6
2845.2
3170.8
3496.4
3822.0

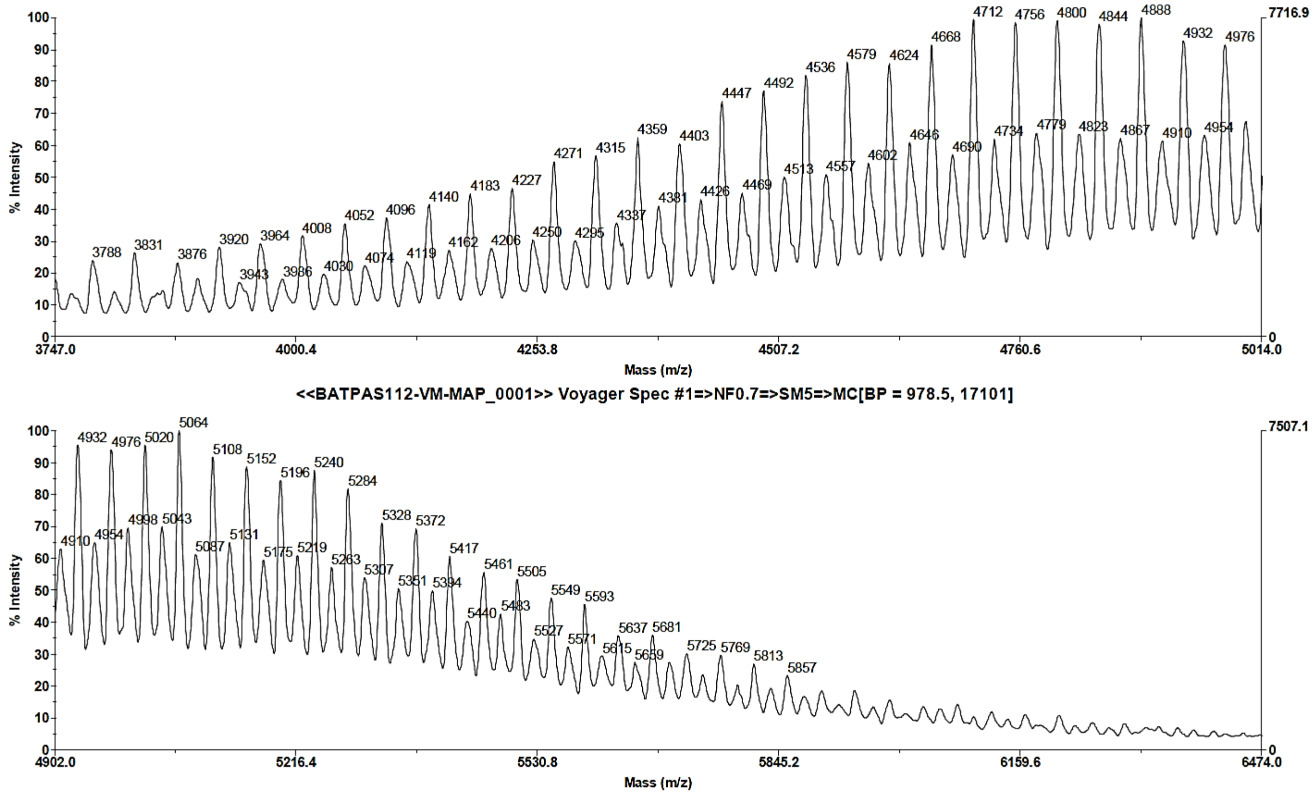

Figure S57. Mass spectra of GPC of Compound Type II (5 kDa). (THF PosLin [1:5] (Dith;THF) +NaOAc)

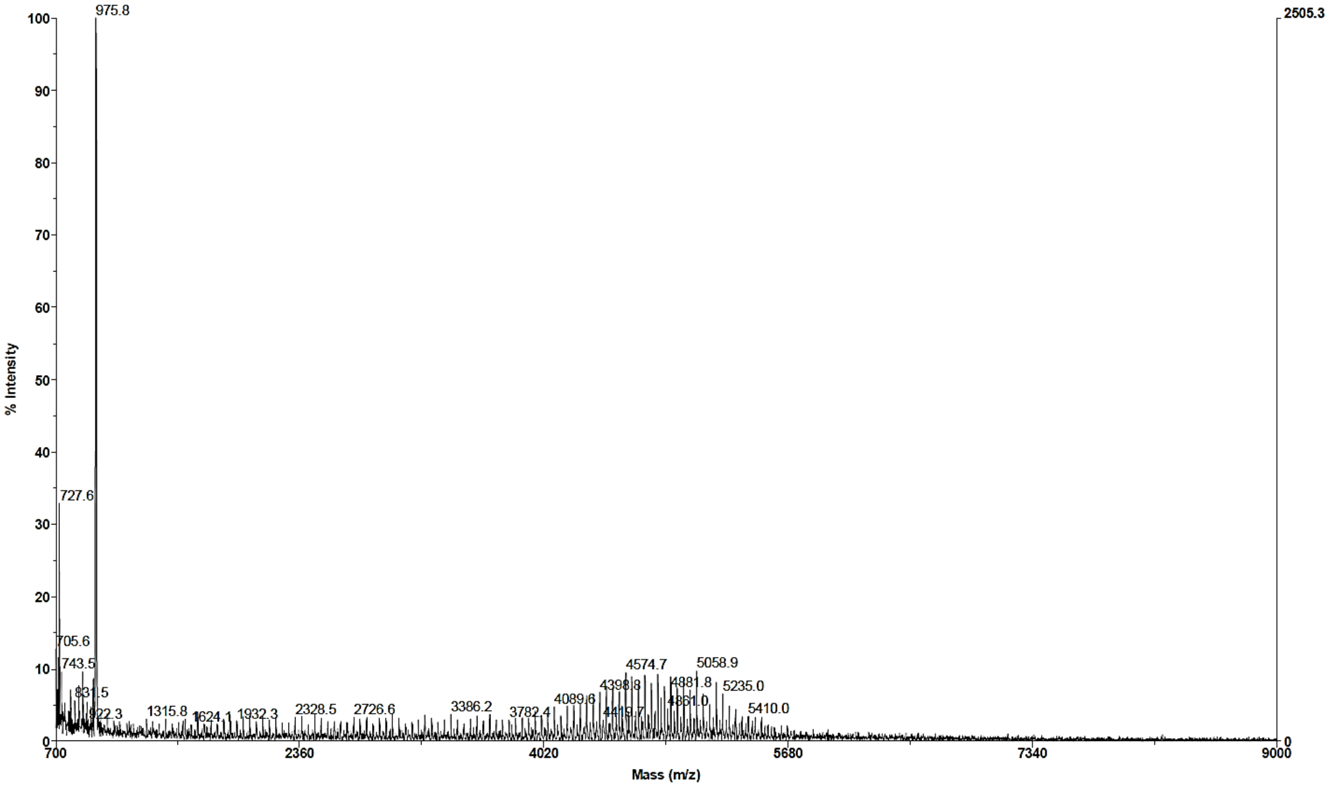

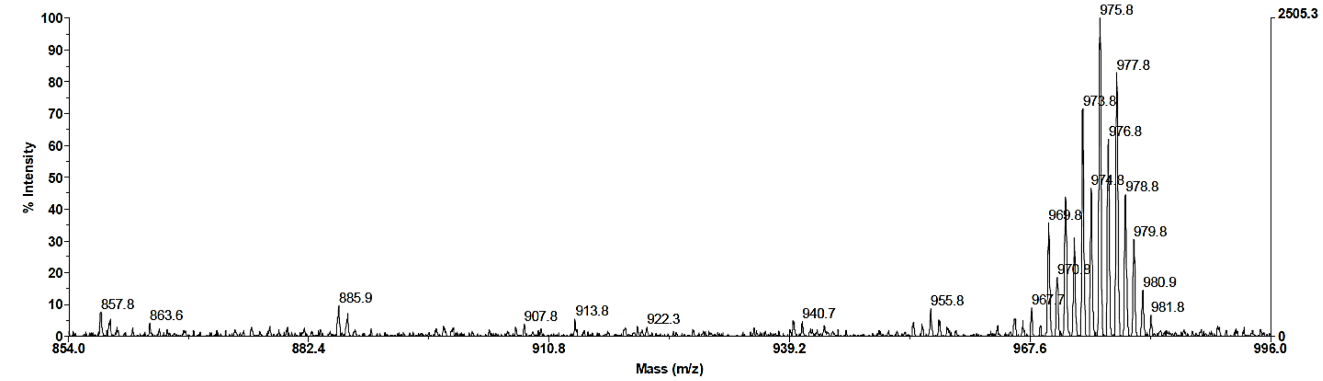

<<BATPAS112-VM-MAP-2_0001>> Voyager Spec #1=>AdvBC(64,0.5,0.1)=>SM5=>MC[BP = 975.8, 2505]

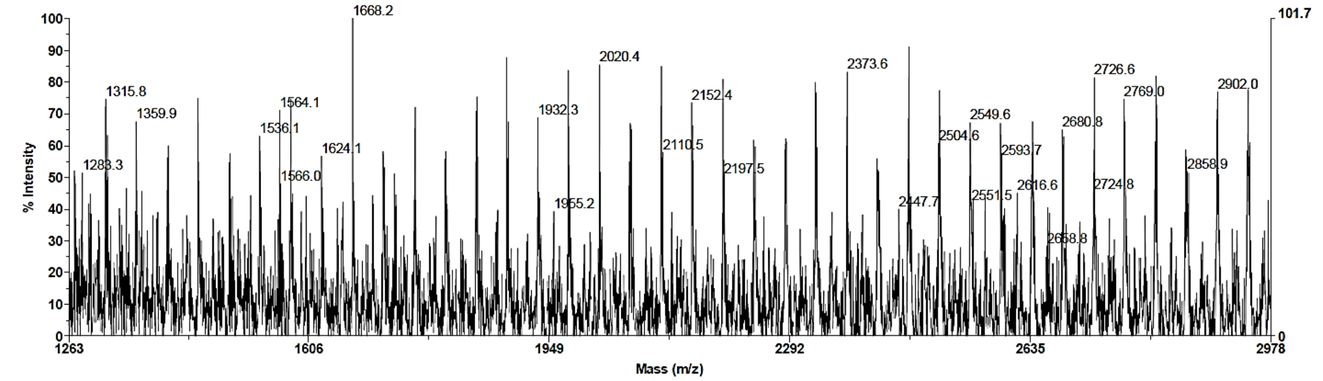

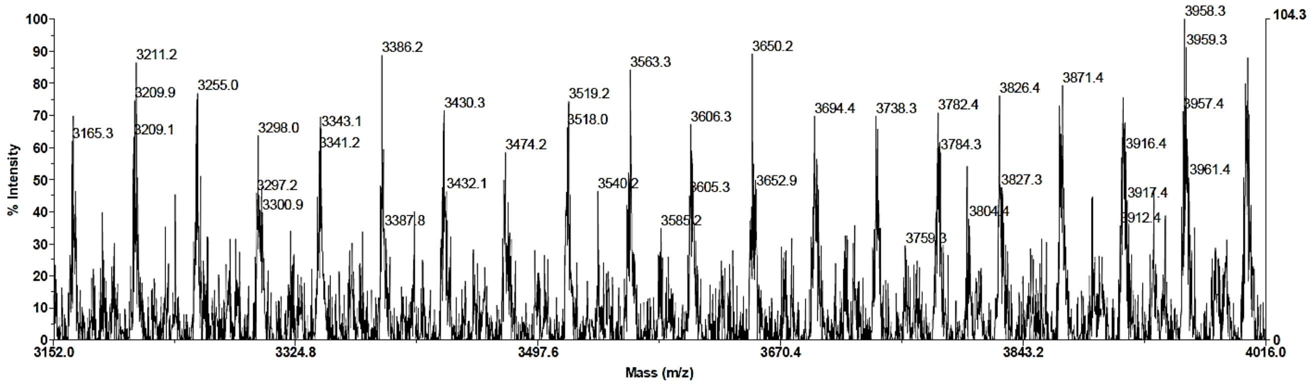

<<BATPAS112-VM-MAP-2_0001>> Voyager Spec #1=>AdvBC(64,0.5,0.1)=>SM5=>MC[BP = 975.8, 2505]

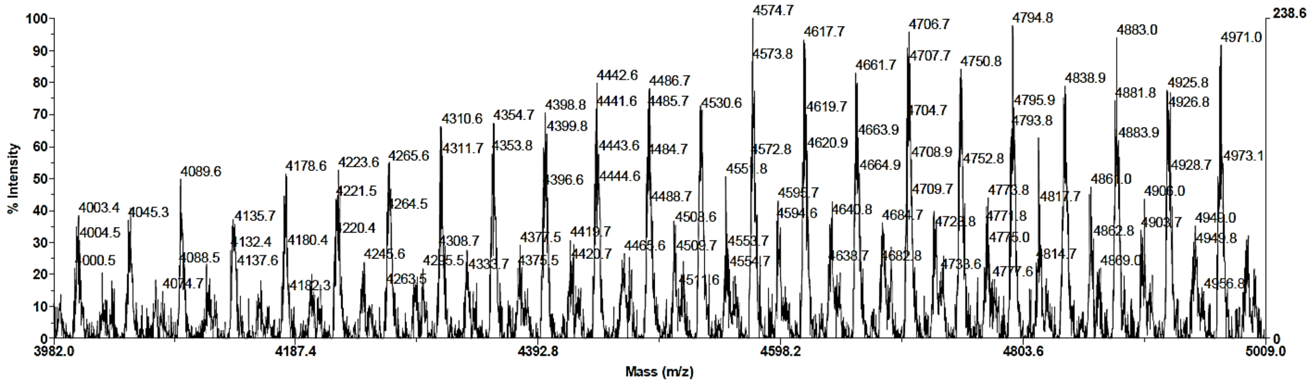

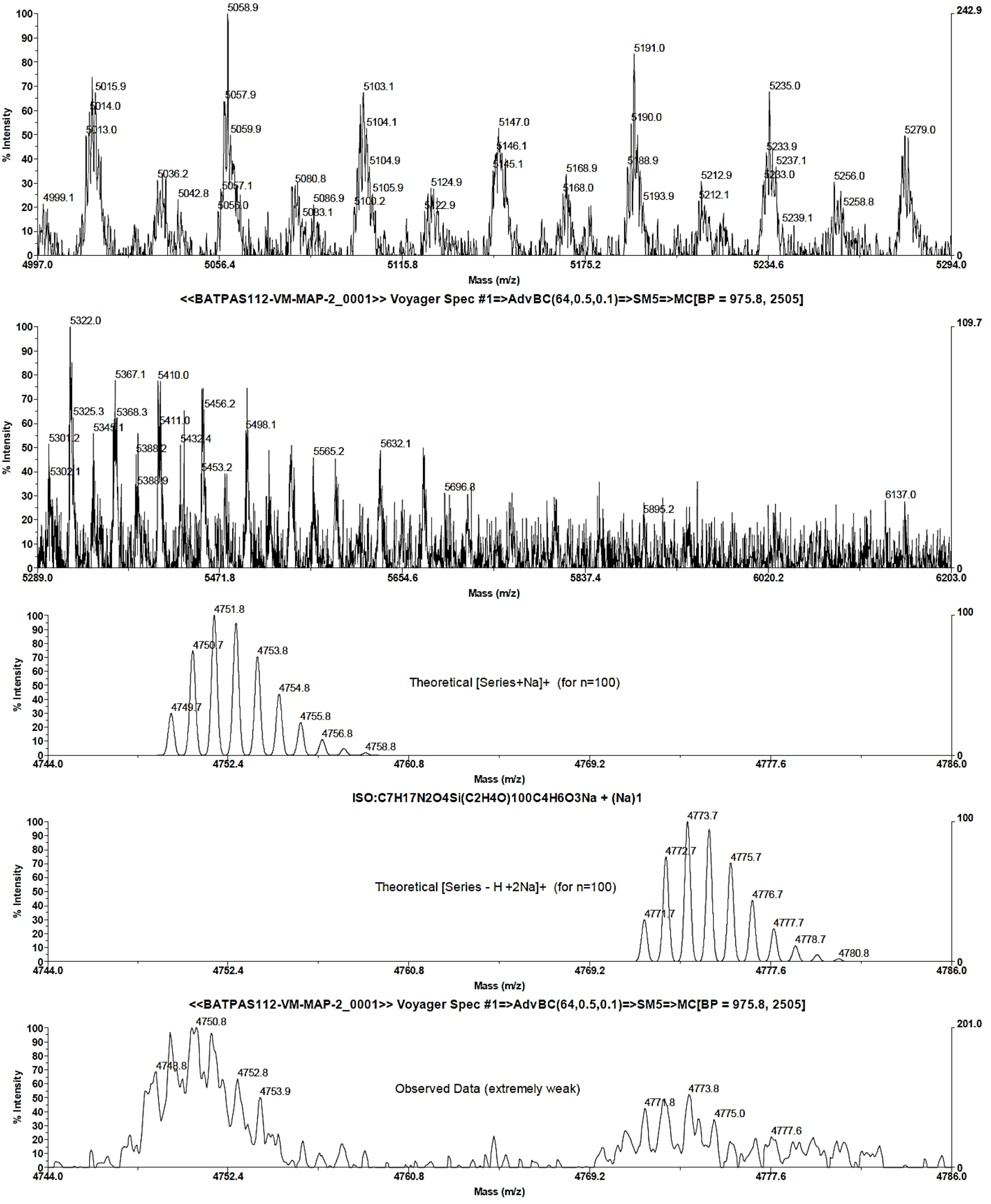

Figure S58. Mass spectra of GPC of Compound Type II (5 kDa). (THF PosRef [1:5] (Dith;THF) +NaOAc)

Confocal fluorescence imaging microscopy of $Y_{0.9}Er_{0.1}VO_4$ and corresponding functional composites

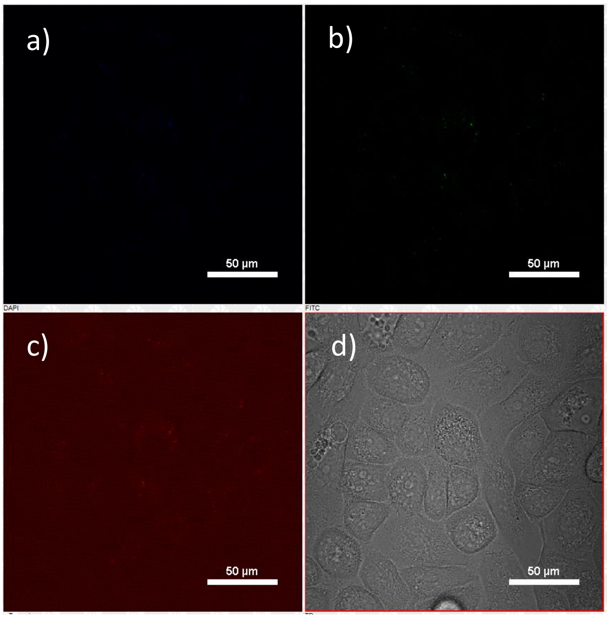

Figure S59. Confocal image of live PC-3 control cells. λ_{ex} = 405 nm; Scale bar: 50 µm; a) blue channel λ_{em} = 420-480 nm; b) green channel; λ_{em} = 516-530 nm; c) red channel λ_{em} = 615-650 nm; d) DIC channel.

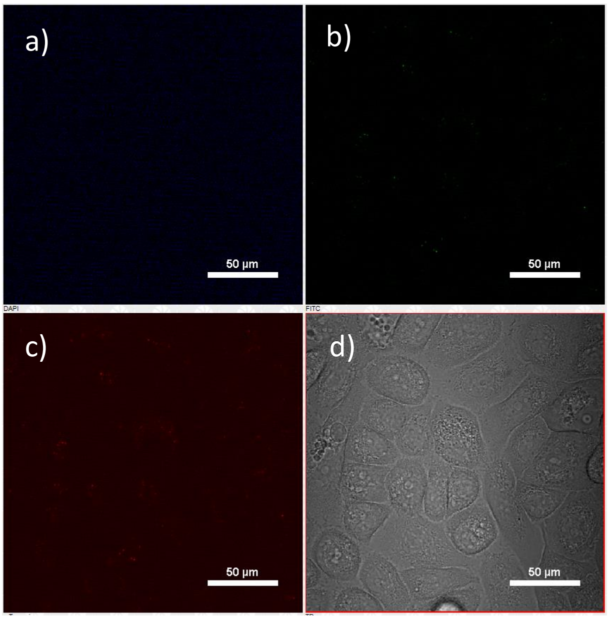

Figure S60. Confocal image of live PC-3 control cells. λ_{ex} = 488 nm. a) blue channel λ_{em} = 420-480 nm; b) green channel; λ_{em} = 516-530 nm; c) red channel λ_{em} = 615-650 nm; d) DIC channel.

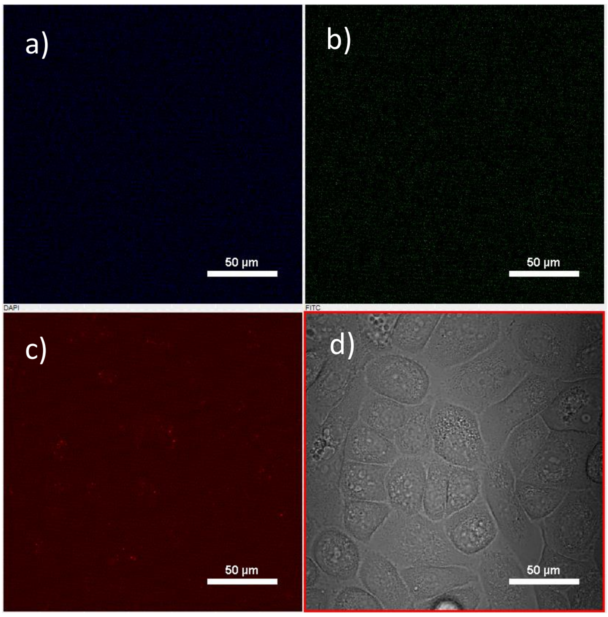

Figure S61. Confocal image of live PC-3 control cells. λ_{ex} = 561 nm. a) blue channel λ_{em} = 420-480 nm; b) green channel; λ_{em} = 516-530 nm; c) red channel λ_{em} = 615-650 nm; d) DIC channel.

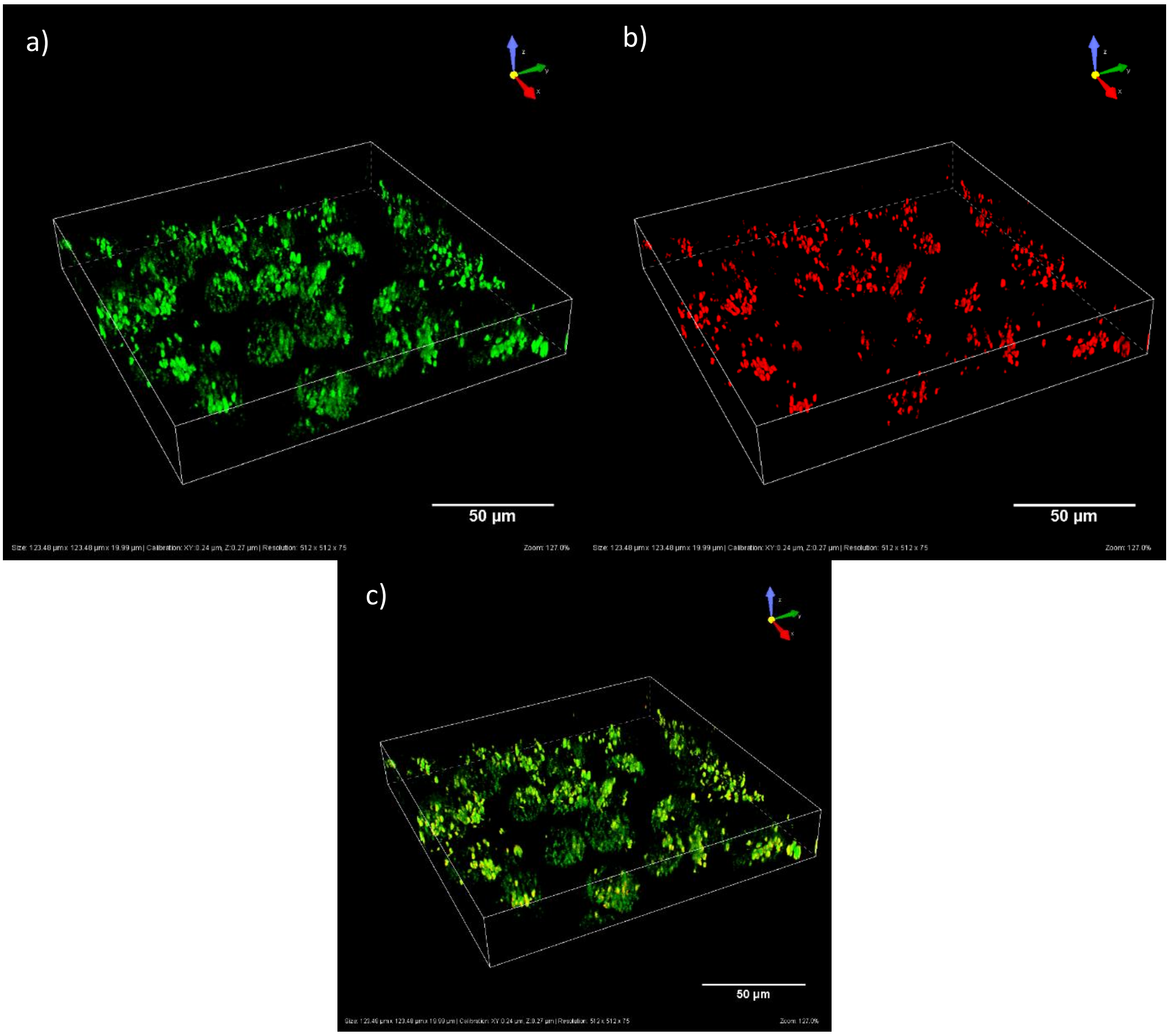

Figure S62. Confocal fluorescence image of live PC-3 cells incubated with the $Y_{0.9}Er_{0.1}VO_4$@Chitosan composite at 1 mg/ml for 15 min. 3D imaging representation. a) green channel λ_{em} = 516-530 nm; b) red channel λ_{em} = 615-650; c) overlay of green and red channels. λ_{ex} = 488 nm.

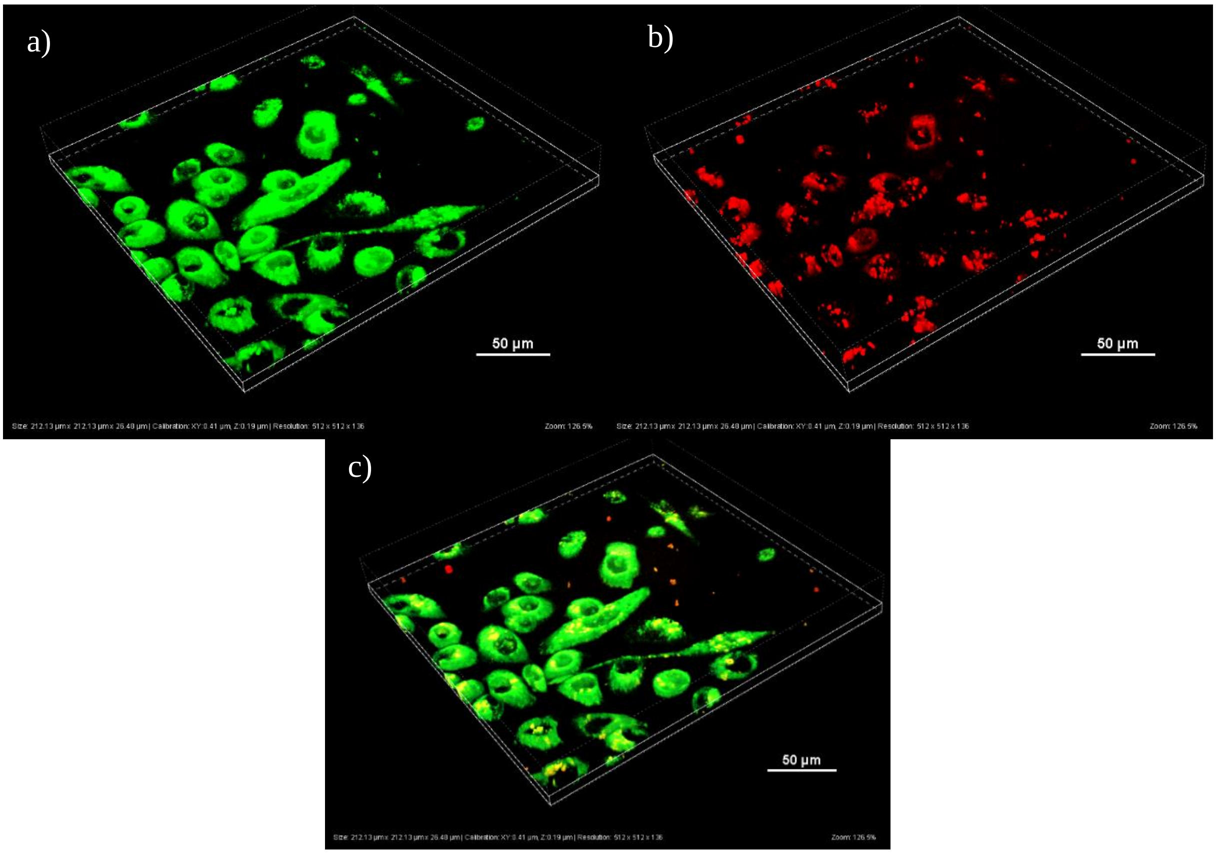

Figure S63. 3D confocal fluorescence imaging of live PC-3 cells incubated with $Y_{0.9}Eu_{0.1}VO_4@SiO_2$ composite at 1 mg/ml for 15 min. a) green channel λ_{em} = 516-530 nm; b) red channel λ_{em} = 615-650; c) overlay of green and red channels. λ_{ex} = 488 nm.

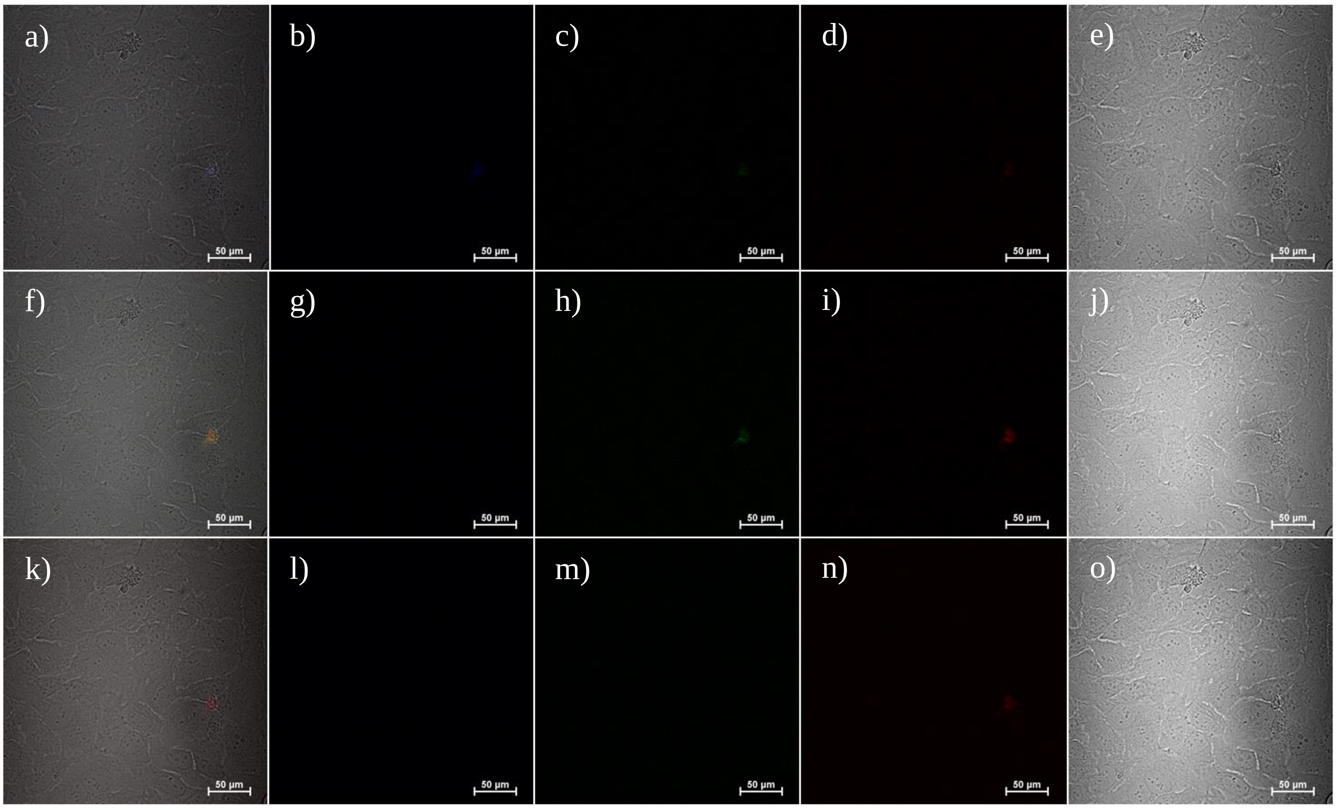

Figure S64. Confocal fluorescence imaging of live HeLa cells (control experiment, 15 min incubation, 1% DMSO, 37 ºC). a),f),k) merged channel, b),g),l) blue channel; c),h),m) green channel; d),i),n) red channel; e),j),o) DIC channel of three different areas, λ_{ex} = 405 nm; a) to e), λ_{ex} = 488 nm; f) to j), λ_{ex} = 561 nm; k) to o). Scale bar: 50 µm.

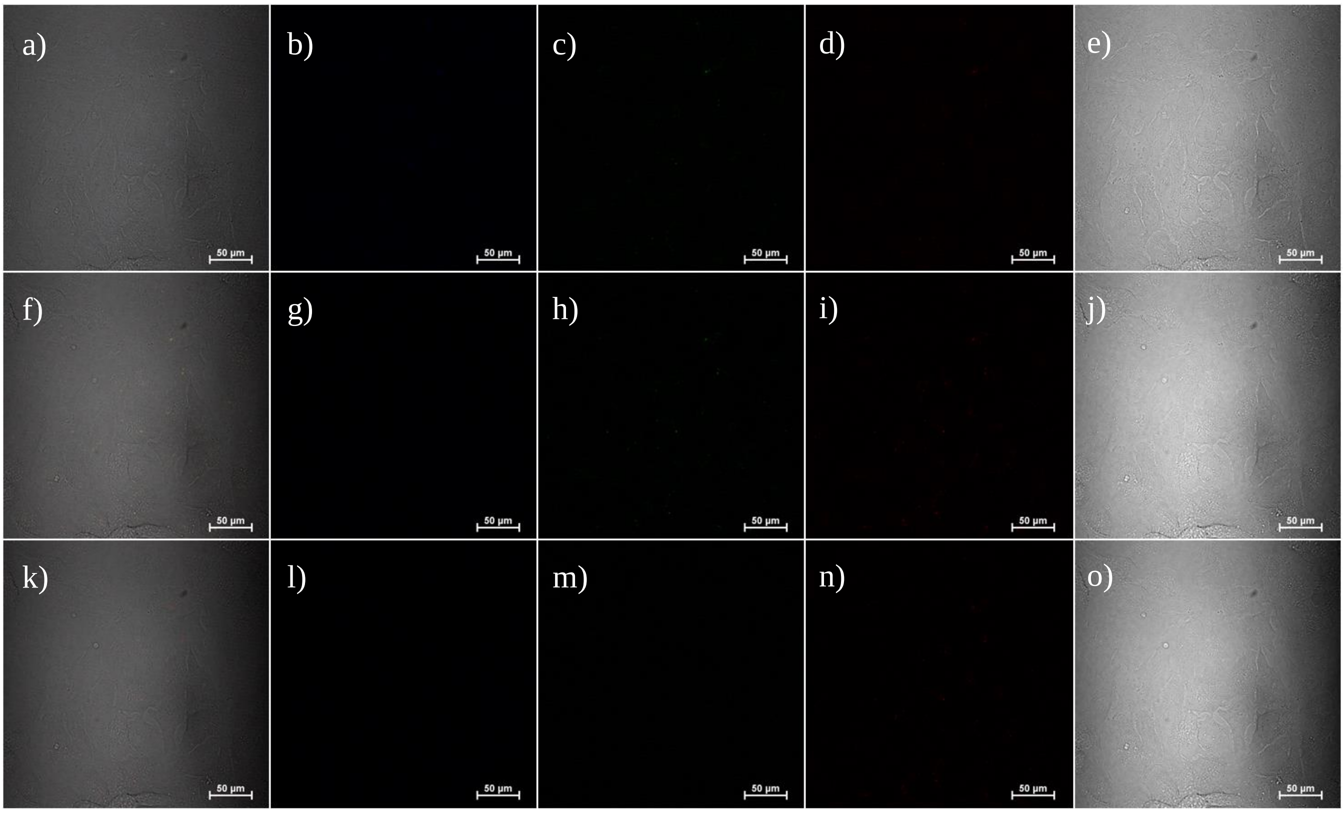

Figure S65. Confocal image of live HeLa control cells (15 min, 1% DMSO, 37 ºC). a),f),k) merged channel, b),g),l) blue channel; c),h),m) green channel; d),i),n) red channel; e),j),o) DIC channel of three different areas, λ_{ex} = 405 nm; a) to e), λ_{ex} = 488 nm; f) to j), λ_{ex} = 561 nm; k) to o). Scale bar: 50 μm.

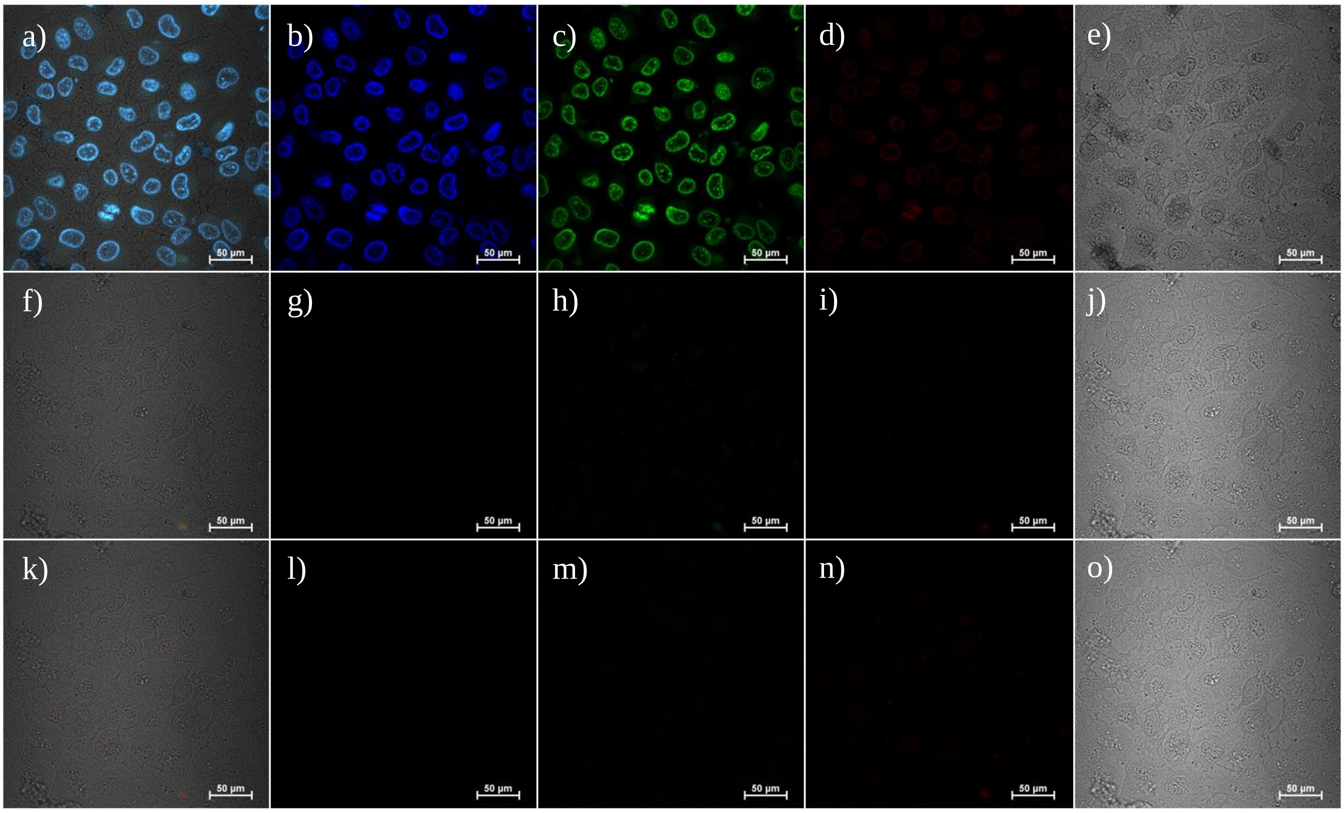

Figure S66. Confocal fluorescence imaging of live HeLa cells: 15 min incubation (as per control conditions) followed by addition of 1 µg/mL Hoechst dye and 30 min incubation (1% DMSO, 37 ºC). a),f),k) merged channel, b),g),l) blue channel; c),h),m) green channel; d),i),n) red channel; e),j),o) DIC channel of three different areas, λ_{ex} = 405 nm; a) to e), λ_{ex} = 488 nm; f) to j), λ_{ex} = 561 nm; k) to o). Scale bar: 50 μm.

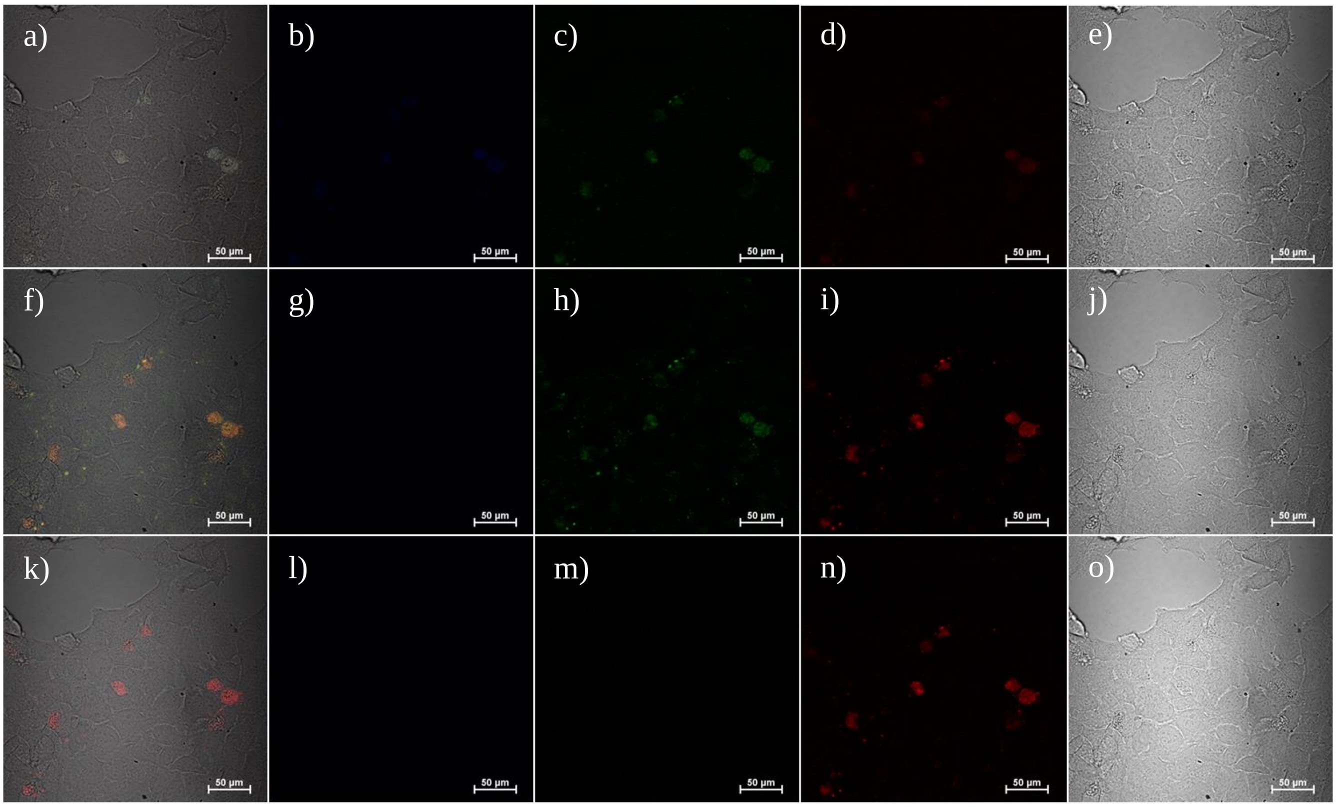

Figure S67. Confocal fluorescence imaging of live HeLa cells incubated with $Y_{0.9}Eu_{0.1}VO_4@SiO_2$ composite at 10 µg/ml for 15 min (1% DMSO, 37 ºC) a),f),k) merged channel, b),g),l) blue channel; c),h),m) green channel; d),i),n) red channel; e),j),o) DIC channel of three different areas, λ_{ex} = 405 nm; a) to e), λ_{ex} = 488 nm; f) to j), λ_{ex} = 561 nm; k) to o). Scale bar: 50 µm.

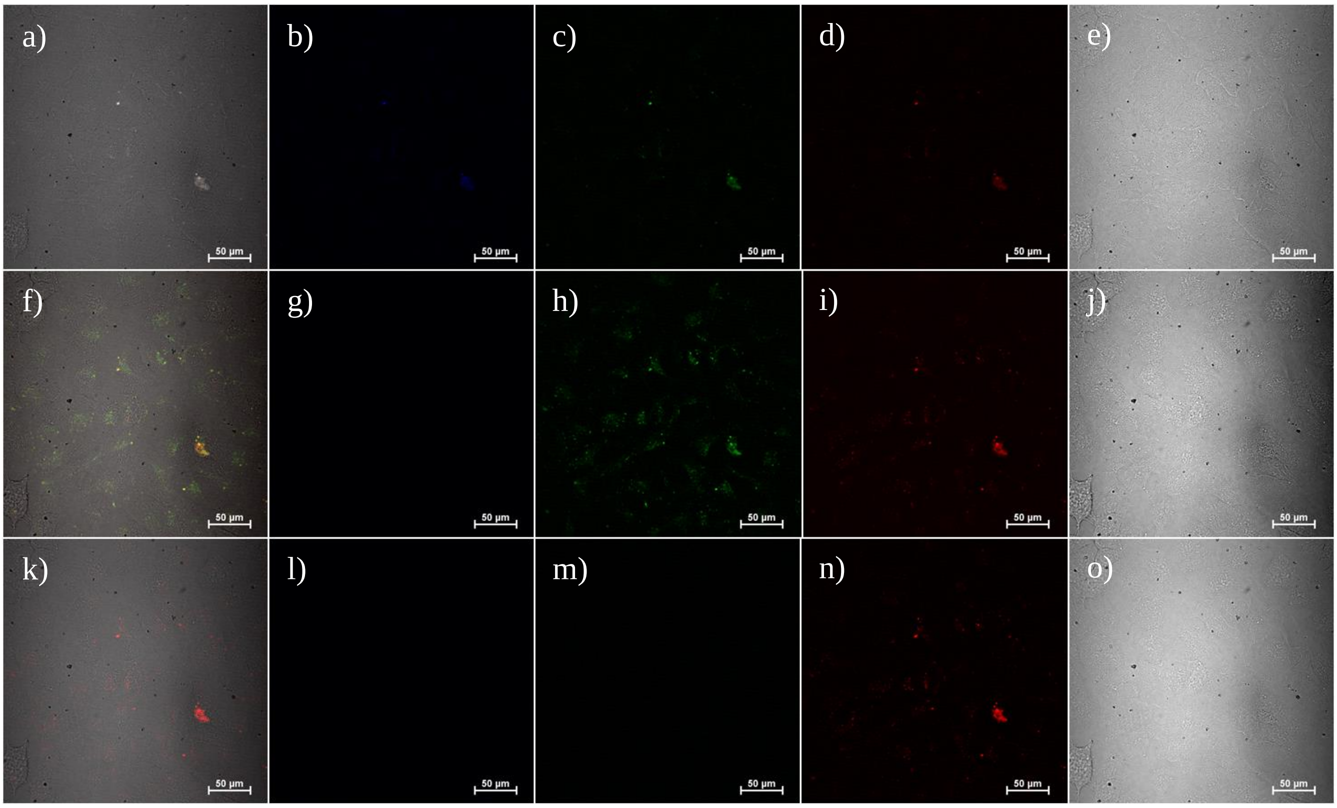

Figure S68. Confocal fluorescence imaging of live HeLa cells incubated with $Y_{0.9}Eu_{0.1}VO_4@SiO_2$ composite at 10 µg/ml for 15 min (1% DMSO, 37 ºC). a),f),k) merged channel, b),g),l) blue channel; c),h),m) green channel; d),i),n) red channel; e),j),o) DIC channel of three different areas, λ_{ex} = 405 nm; a) to e), λ_{ex} = 488 nm; f) to j), λ_{ex} = 561 nm; k) to o). Scale bar: 50 µm.

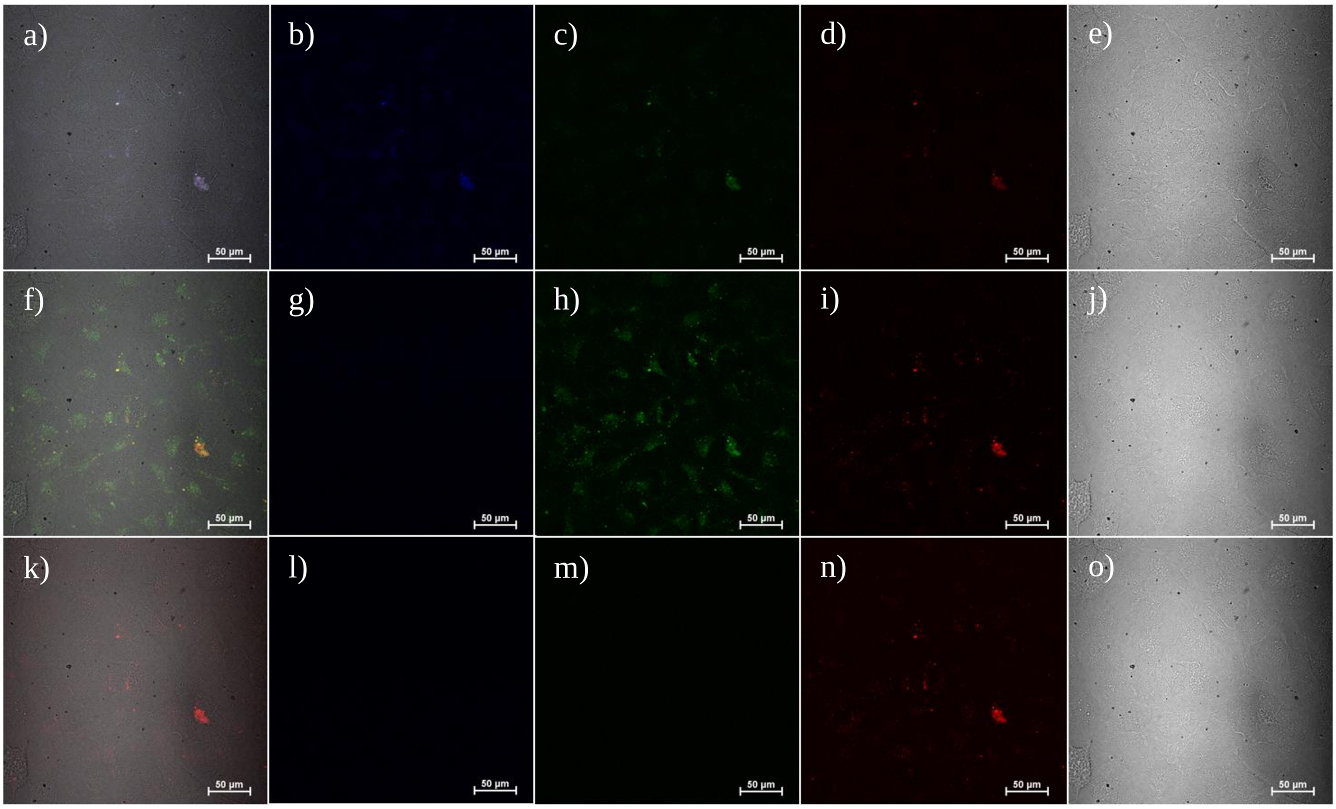

Figure S69. Confocal fluorescence imaging of live HeLa cells incubated with $Y_{0.9}Eu_{0.1}VO_4@SiO_2$ composite at 10 µg/ml for 15 min (1% DMSO, 37 ºC). a),f),k) merged channel, b),g),l) blue channel; c),h),m) green channel; d),i),n) red channel; e),j),o) DIC channel of three different areas, λ_{ex} = 405 nm; a) to e), λ_{ex} = 488 nm; f) to j), λ_{ex} = 561 nm; k) to o). Scale bar: 50 µm.

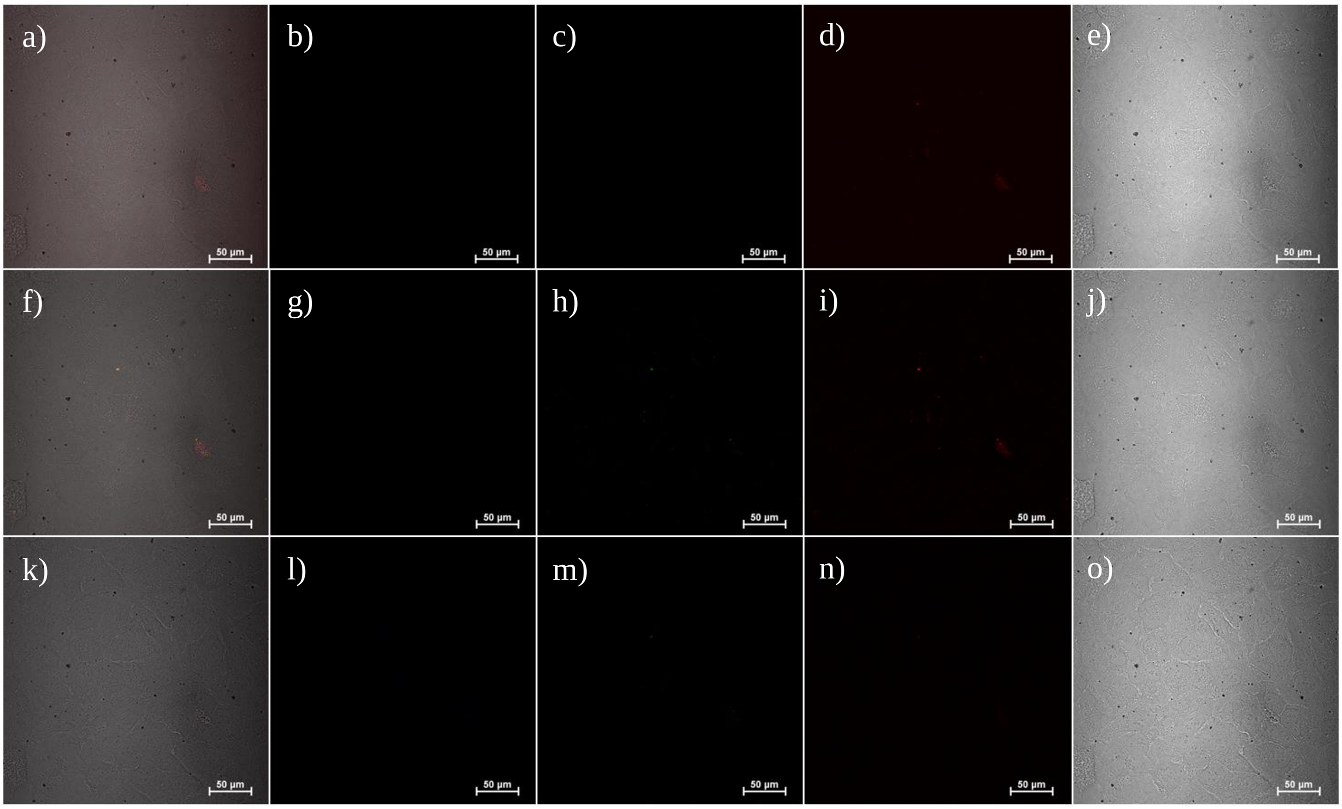

Figure S70. Confocal fluorescence imaging of live HeLa cells incubated with $Y_{0.9}Eu_{0.1}VO_4@SiO_2$ composite at 10 µg/ml for 15 min (1% DMSO, 37 ºC). a),f),k) merged channel, b),g),l) blue channel; c),h),m) green channel; d),i),n) red channel; e),j),o) DIC channel of three different areas, λ_{ex} = 405 nm; a) to e), λ_{ex} = 488 nm; f) to j), λ_{ex} = 561 nm; k) to o). Scale bar: 50 µm.

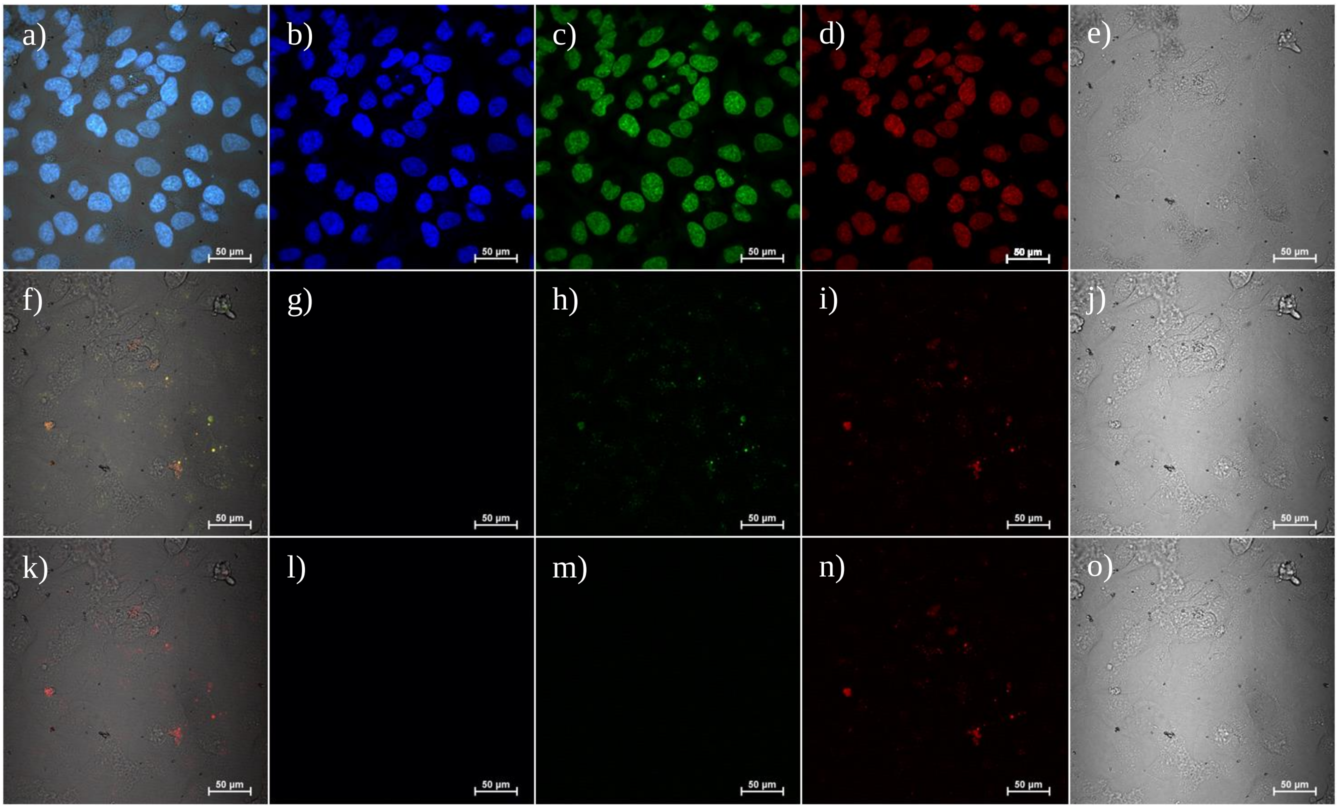

Figure S71. Confocal fluorescence imaging of live HeLa cells incubated with $Y_{0.9}Eu_{0.1}VO_4@SiO_2$ composite at 10 µg/ml for 15 min + 1µg/mL Hoechst dye for 10 min (1% DMSO, 37 ºC). a),f),k) merged channel, b),g),l) blue channel; c),h),m) green channel; d),i),n) red channel; e),j),o) DIC channel of three different areas, λ_{ex} = 405 nm; a) to e), λ_{ex} = 488 nm; f) to j), λ_{ex} = 561 nm; k) to o). Scale bar: 50 µm.

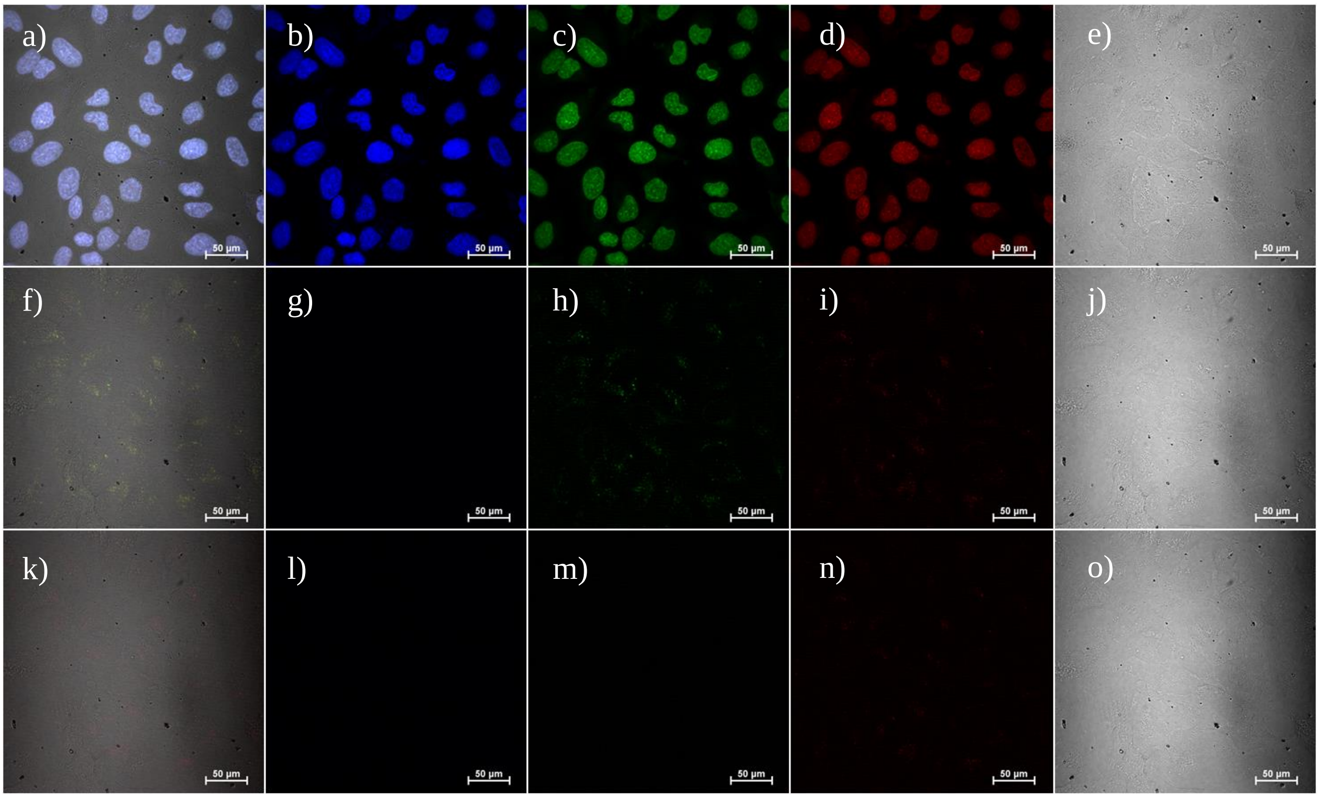

Figure S72. Confocal fluorescence imaging of live HeLa cells incubated with $Y_{0.9}Eu_{0.1}VO_4@SiO_2$ composite at 10 µg/ml for 15 min + 1µg/mL Hoechst dye for 10 min (1% DMSO, 37 ºC). a),f),k) merged channel, b),g),l) blue channel; c),h),m) green channel; d),i),n) red channel; e),j),o) DIC channel of three different areas, λ_{ex} = 405 nm; a) to e), λ_{ex} = 488 nm; f) to j), λ_{ex} = 561 nm; k) to o). Scale bar: 50 µm.

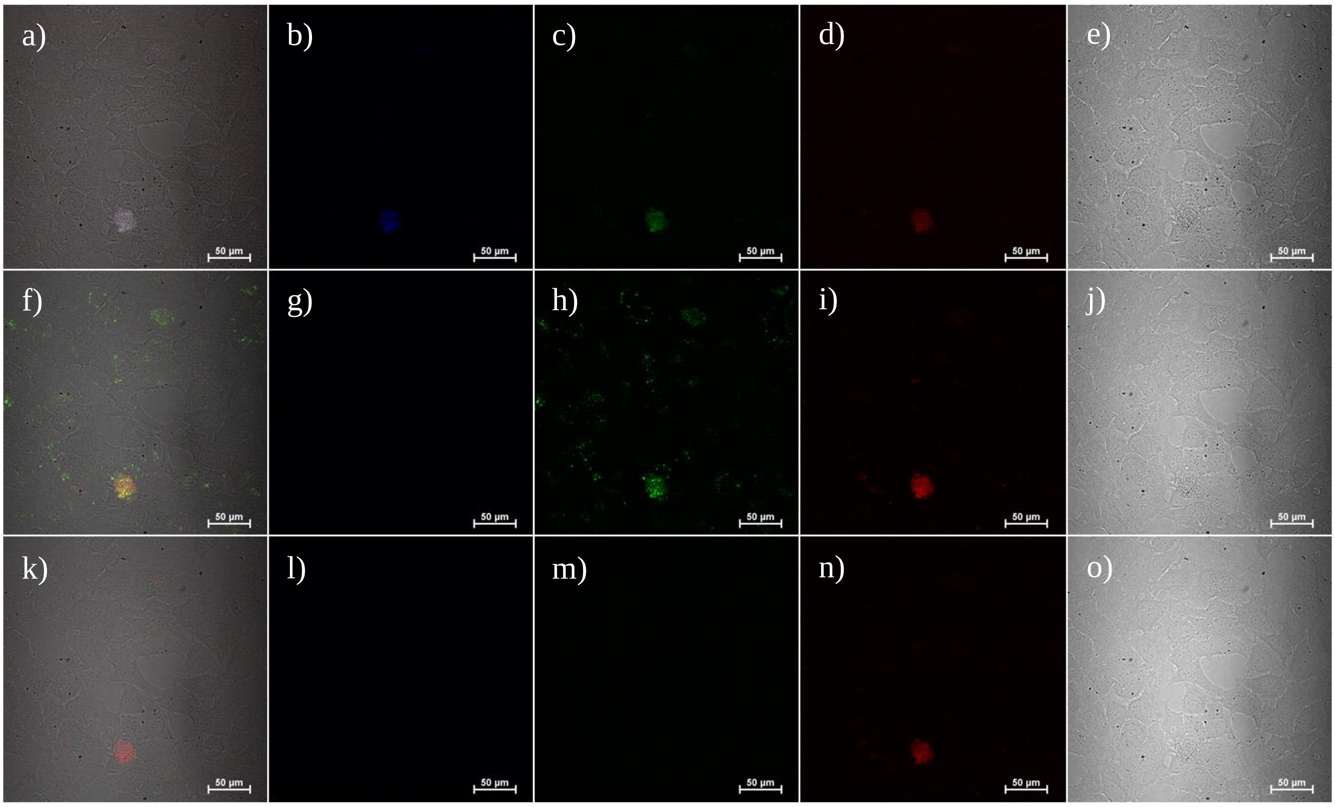

Figure S73. Confocal fluorescence imaging of live HeLa cells incubated with $Y_{0.9}Eu_{0.1}VO_4@SiO_2$ composite at 10 µg/ml for 24 hours (1% DMSO, 37 ºC). a),f),k) merged channel, b),g),l) blue channel; c),h),m) green channel; d),i),n) red channel; e),j),o) DIC channel of three different areas, λ_{ex} = 405 nm; a) to e), λ_{ex} = 488 nm; f) to j), λ_{ex} = 561 nm; k) to o). Scale bar: 50 µm.

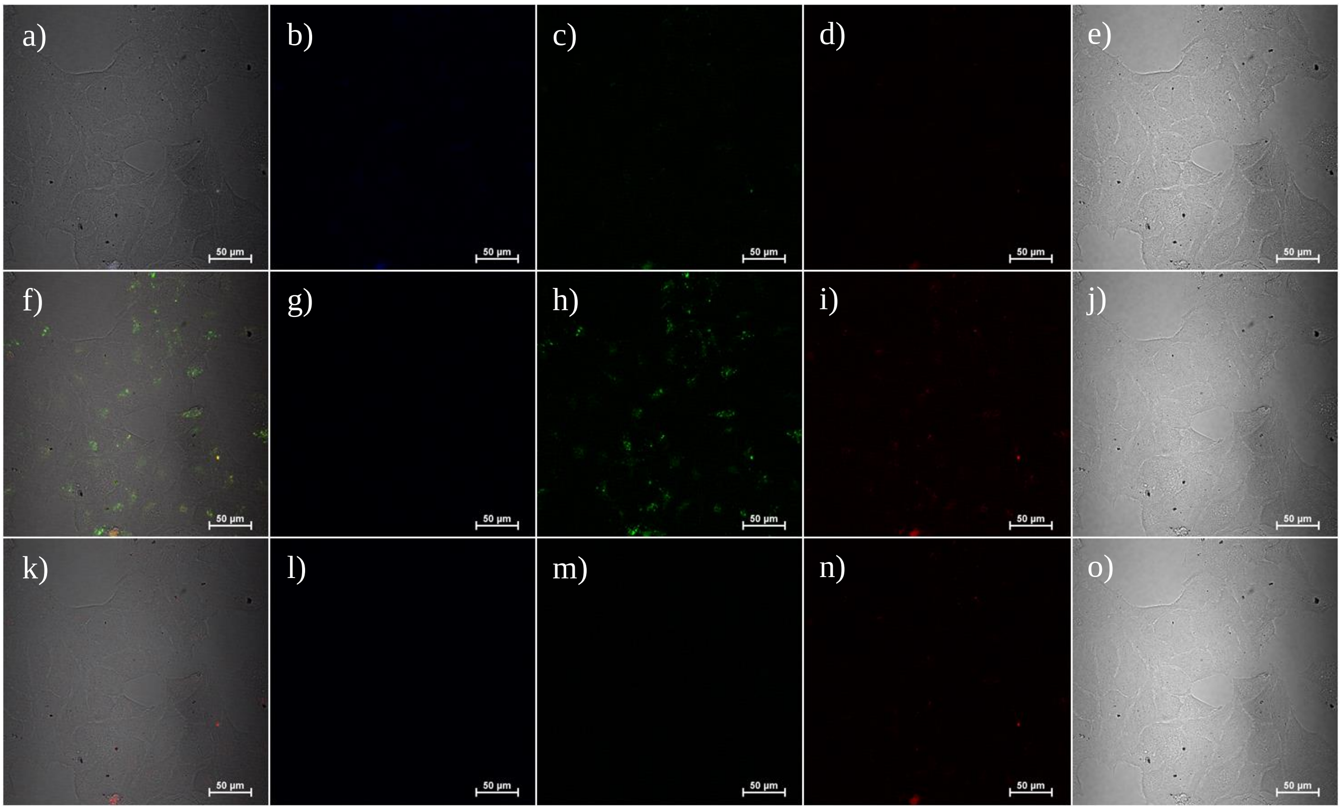

Figure S74. Confocal fluorescence imaging of live HeLa cells incubated with $Y_{0.9}Eu_{0.1}VO_4@SiO_2$ composite at 10 µg/ml for 24 hours (1% DMSO, 37 ºC). a),f),k) merged channel, b),g),l) blue channel; c),h),m) green channel; d),i),n) red channel; e),j),o) DIC channel of three different areas, λ_{ex} = 405 nm; a) to e), λ_{ex} = 488 nm; f) to j), λ_{ex} = 561 nm; k) to o). Scale bar: 50 µm.

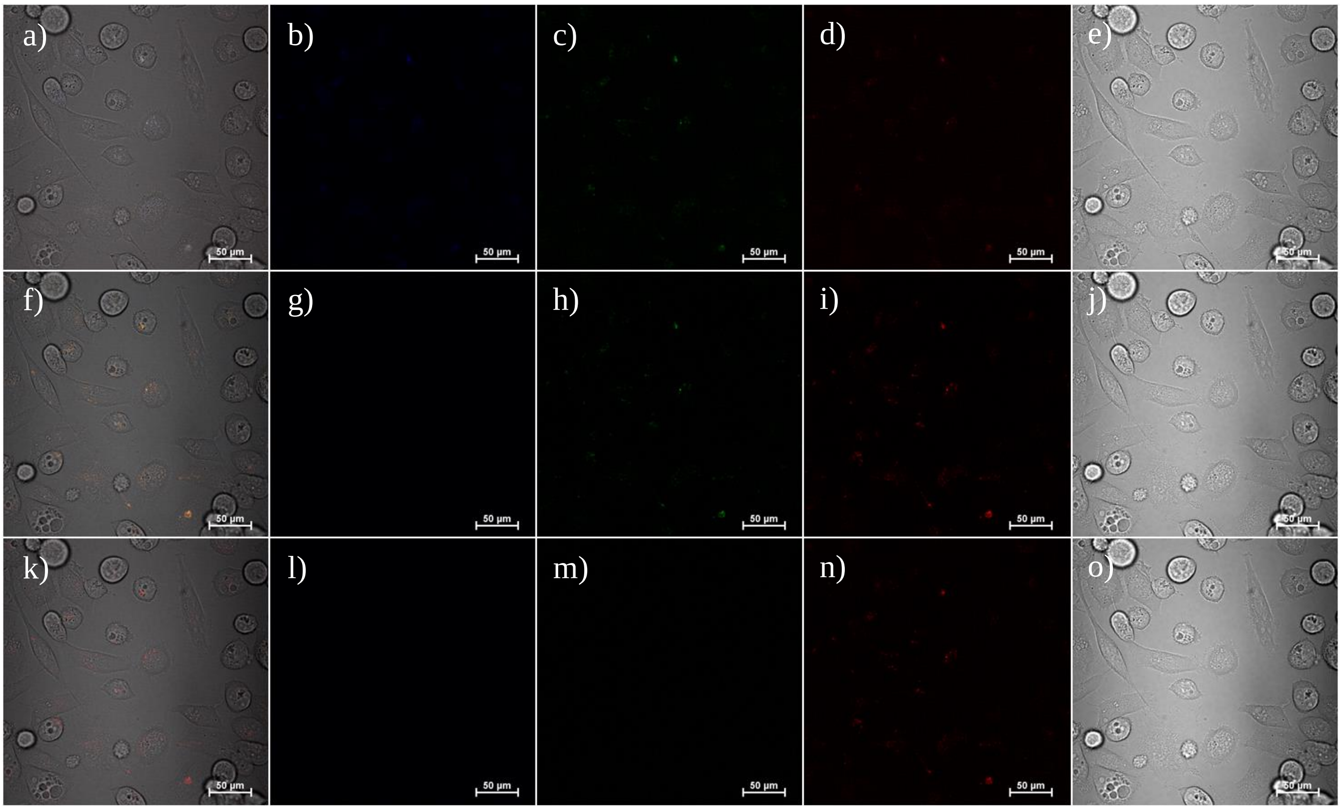

Figure S75. Confocal fluorescence imaging of live PC3 cells (controls, 15 min incubation, 1% DMSO, 37 ºC). a),f),k) merged channel, b),g),l) blue channel; c),h),m) green channel; d),i),n) red channel; e),j),o) DIC channel of three different areas, λ_{ex} = 405 nm; a) to e), λ_{ex} = 488 nm; f) to j), λ_{ex} = 561 nm; k) to o). Scale bar: 50 μm.

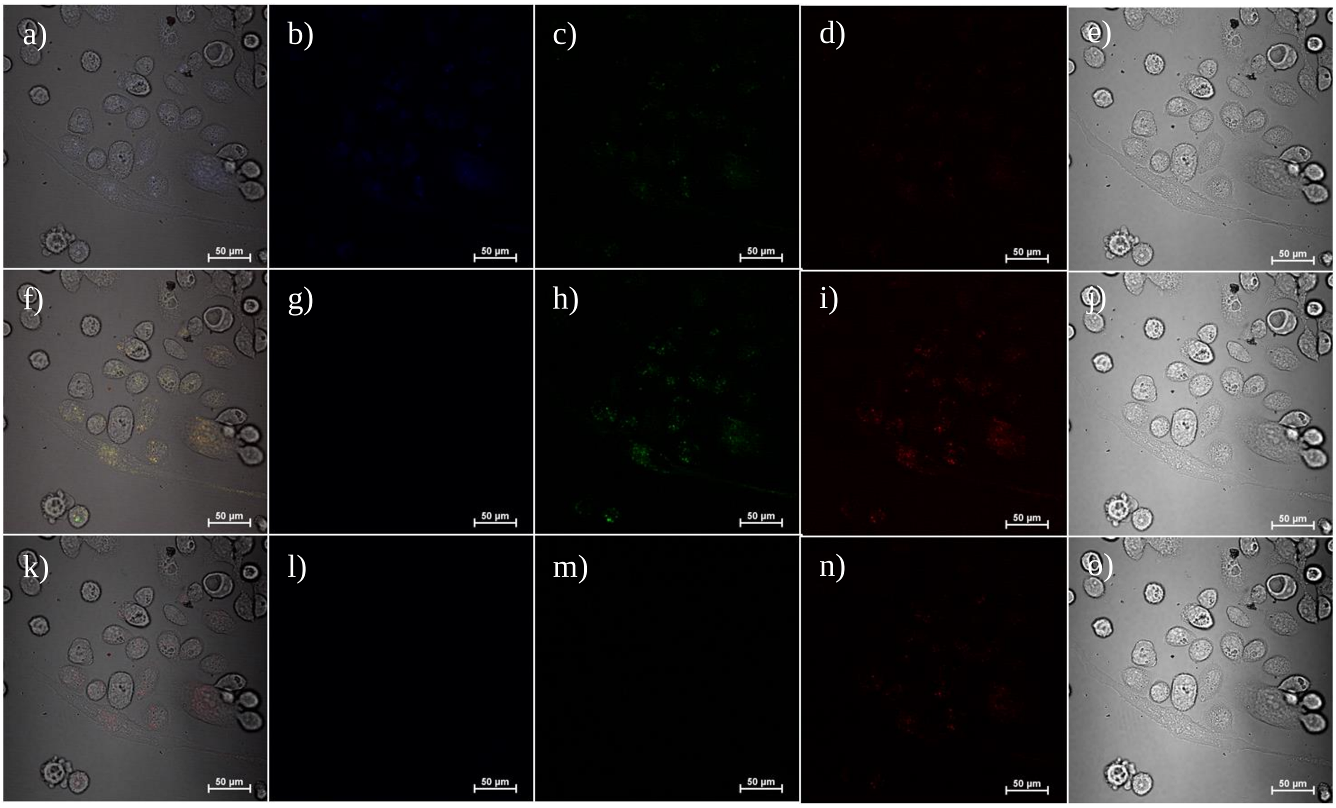

Figure S76. Confocal fluorescence imaging of live PC3 cells incubated with $Y_{0.9}Eu_{0.1}VO_4@SiO_2$ composite at 10 µg/ml for 15 min (1% DMSO, 37 ºC). a),f),k) merged channel, b),g),l) blue channel; c),h),m) green channel; d),i),n) red channel; e),j),o) DIC channel of three different areas, λ_{ex} = 405 nm; a) to e), λ_{ex} = 488 nm; f) to j), λ_{ex} = 561 nm; k) to o). Scale bar: 50 μm.

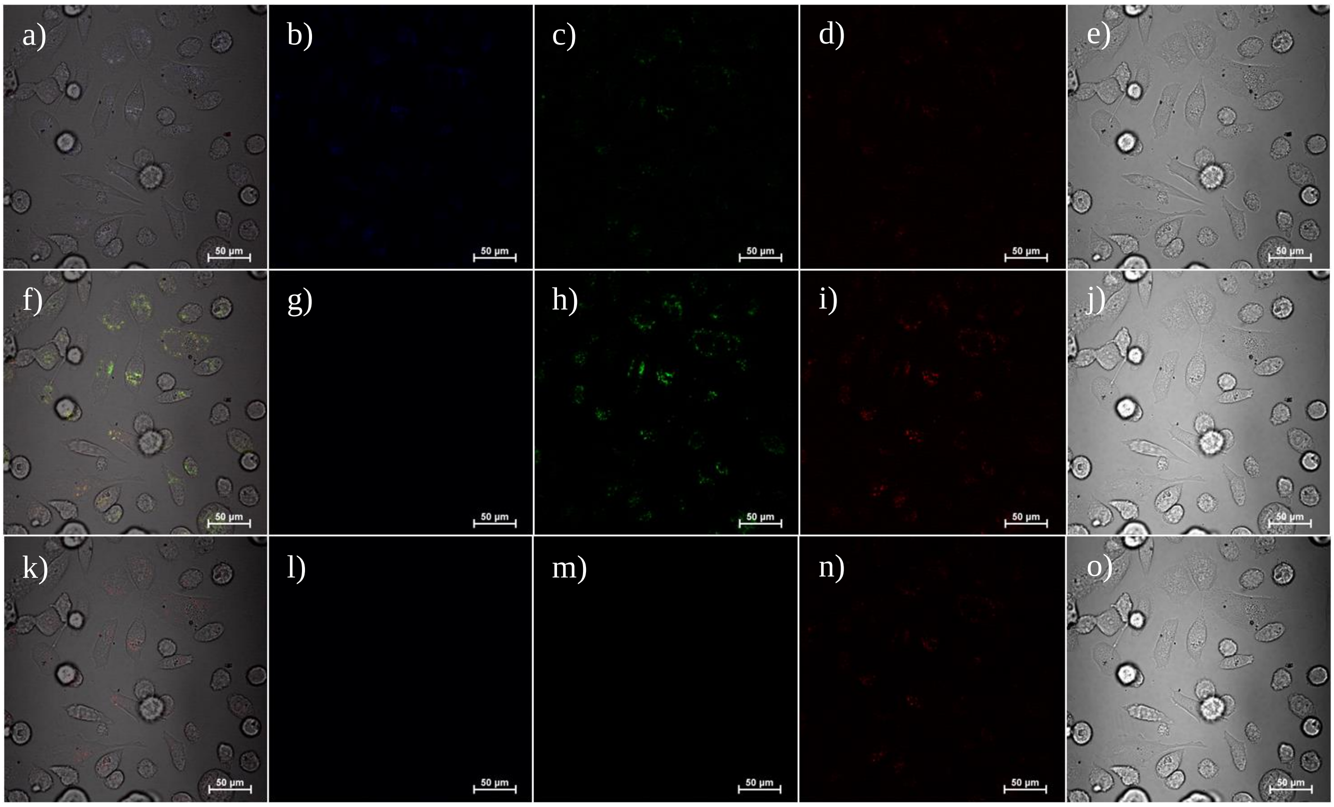

Figure S77. Confocal fluorescence imaging of live PC3 cells incubated with $Y_{0.9}Eu_{0.1}VO_4@SiO_2$ composite at 10 µg/ml for 6 hours (1% DMSO, 37 ºC). a),f),k) merged channels, b),g),l) blue channel; c),h),m) green channel; d),i),n) red channel; e),j),o) DIC channel of three different areas, λ_{ex} = 405 nm; a) to e), λ_{ex} = 488 nm; f) to j), λ_{ex} = 561 nm; k) to o). Scale bar: 50 µm.

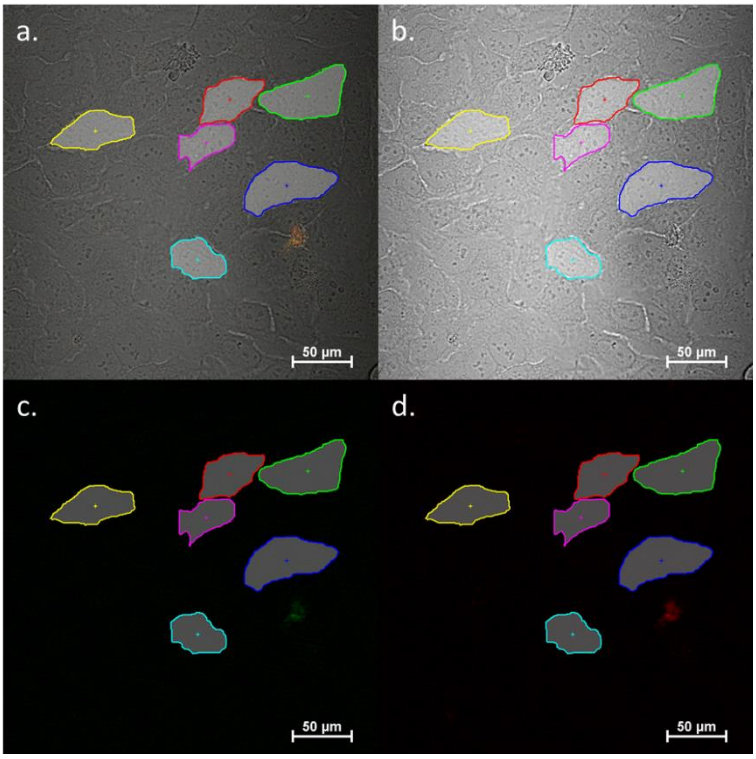

Figure S78. Confocal fluorescence imaging of live HeLa control cells (15 min, 1% DMSO, 37 ºC). a) merged image of green, red and DIC channel, b) DIC channel; c) green channel λ_{ex} = 488 nm, λ_{em} = 515 nm; d) red channel λ_{ex} = 561 nm, λ_{em} = 630 nm. Scale bar: 50 µm. The average fluorescence intensity was calculated from six selected region of interest as shown in the micrograph.

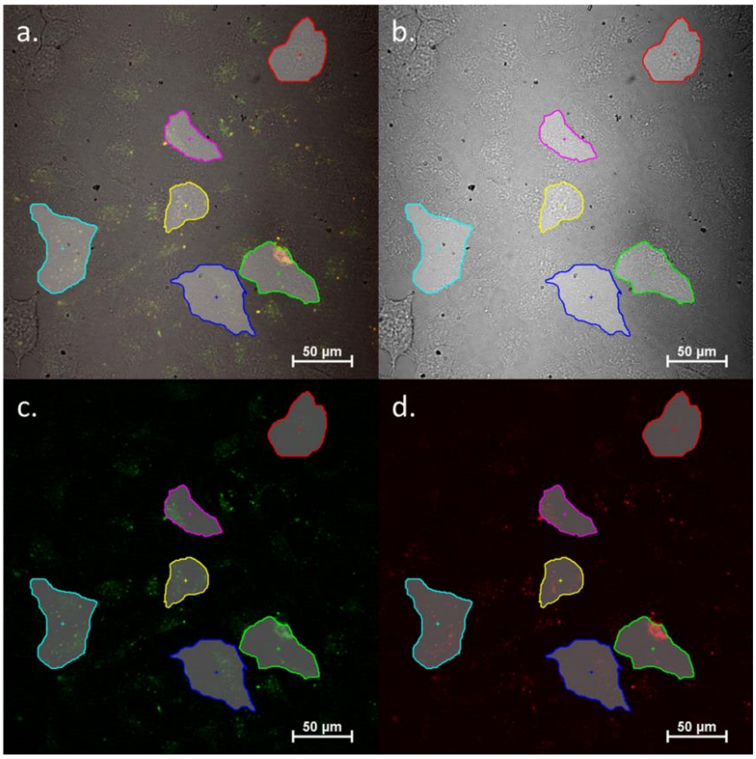

Figure S79. Confocal fluorescence imaging of live HeLa incubated with $Y_{0.9}Eu_{0.1}VO_4@SiO_2$ composite at 10 µg/ml for 15 min (1% DMSO, 37 ºC). a) merged image of green, red and DIC channel, b) DIC channel; c) green channel λ_{ex} = 488 nm, λ_{em} = 515 nm; d) red channel λ_{ex} = 561 nm, λ_{em} = 630 nm. Scale bar: 50 μm. The average fluorescence intensity was calculated from six selected region of interest as shown in the micrograph.

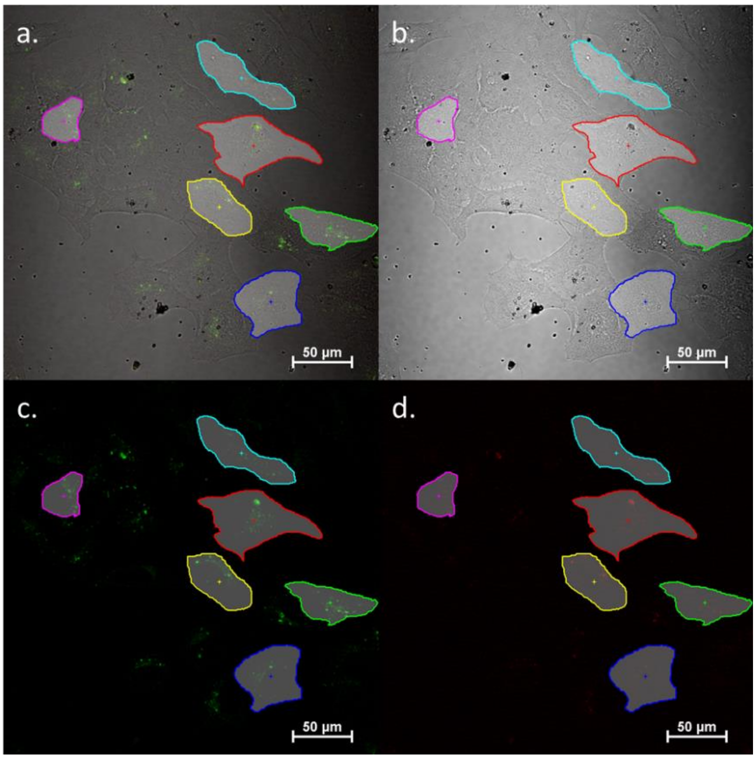

Figure S80. Confocal fluorescence imaging of live HeLa incubated with $Y_{0.9}Eu_{0.1}VO_4@SiO_2$ composite at 10 µg/ml for 6 hours (1% DMSO, 37 ºC). a) merged image of green, red and DIC channel, b) DIC channel; c) green channel λ_{ex} = 488 nm, λ_{em} = 515 nm; d) red channel λ_{ex} = 561 nm, λ_{em} = 630 nm. Scale bar: 50 μm. The average fluorescence intensity was calculated from six selected region of interest as shown in the micrograph.

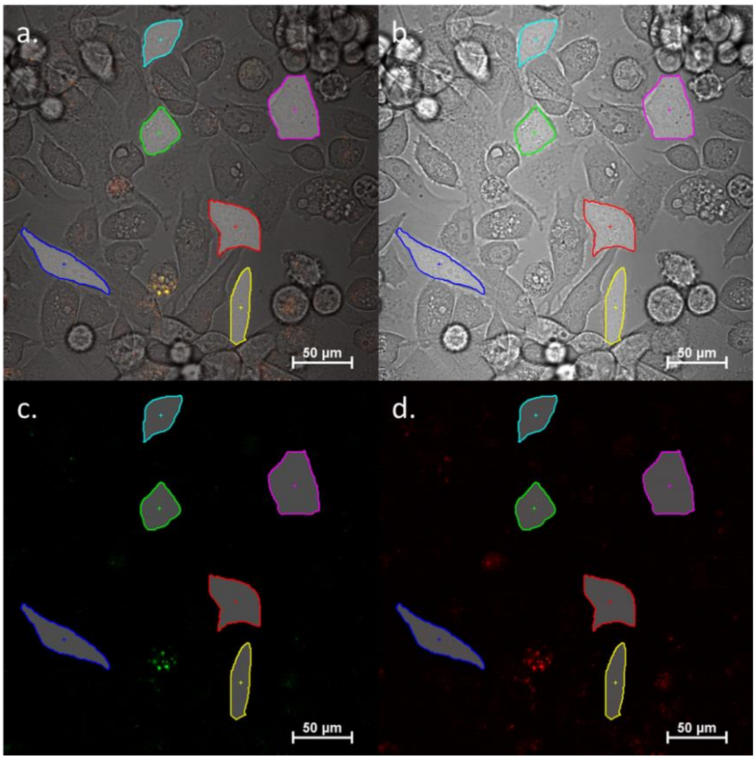

Figure S81. Confocal fluorescence imaging of live PC3 control cells (15 min, 1% DMSO, 37 ºC). a) merged image of green, red and DIC channel, b) DIC channel; c) green channel λ_{ex} = 488 nm, λ_{em} = 515 nm; d) red channel λ_{ex} = 561 nm, λ_{em} = 630 nm. Scale bar: 50 µm. The average fluorescence intensity was calculated from six selected region of interest as shown in the micrograph.

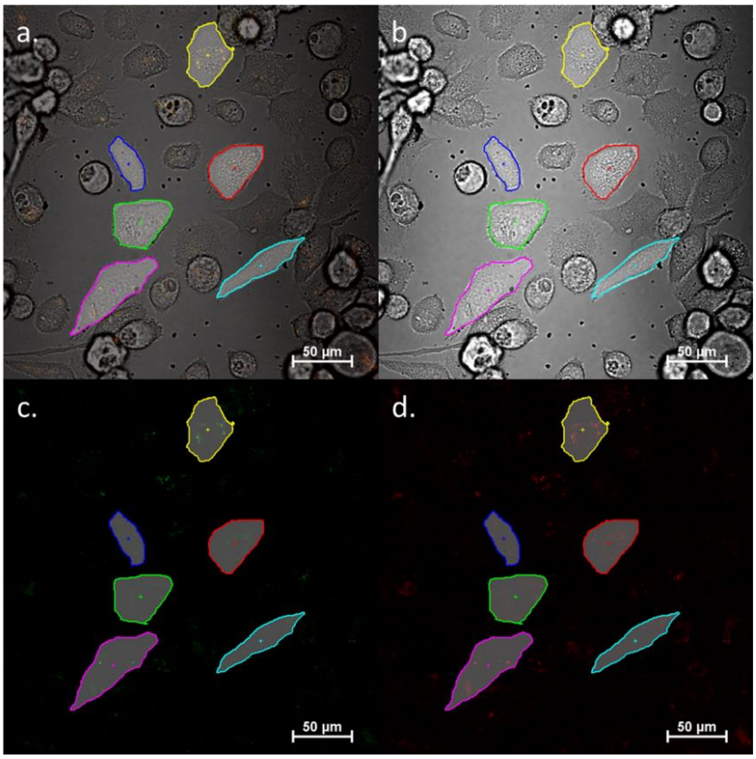

Figure S82. Confocal fluorescence imaging of live PC3 incubated with $Y_{0.9}Eu_{0.1}VO_4@SiO_2$ composite at 10 µg/ml for 15 min (1% DMSO, 37 ºC). a) merged image of green, red and DIC channel, b) DIC channel; c) green channel λ_{ex} = 488 nm, λ_{em} = 515 nm; d) red channel λ_{ex} = 561 nm, λ_{em} = 630 nm. Scale bar: 50 µm. The average fluorescence intensity was calculated from six selected region of interest as shown in the micrograph.

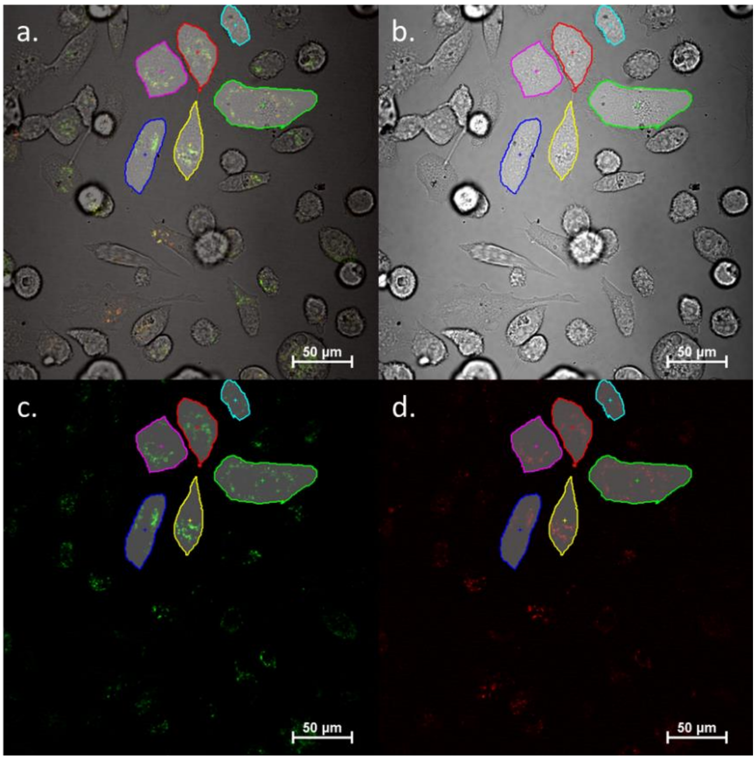

Figure S83. Confocal fluorescence imaging of live PC3 incubated with $Y_{0.9}Eu_{0.1}VO_4@SiO_2$ composite at 10 µg/ml for 6 hours (1% DMSO, 37 ºC). a) merged image of green, red and DIC channel, b) DIC channel; c) green channel λ_{ex} = 488 nm, λ_{em} = 515 nm; d) red channel λ_{ex} = 561 nm, λ_{em} = 630 nm. Scale bar: 50 µm. The average fluorescence intensity was calculated from six selected region of interest as shown in the micrograph.

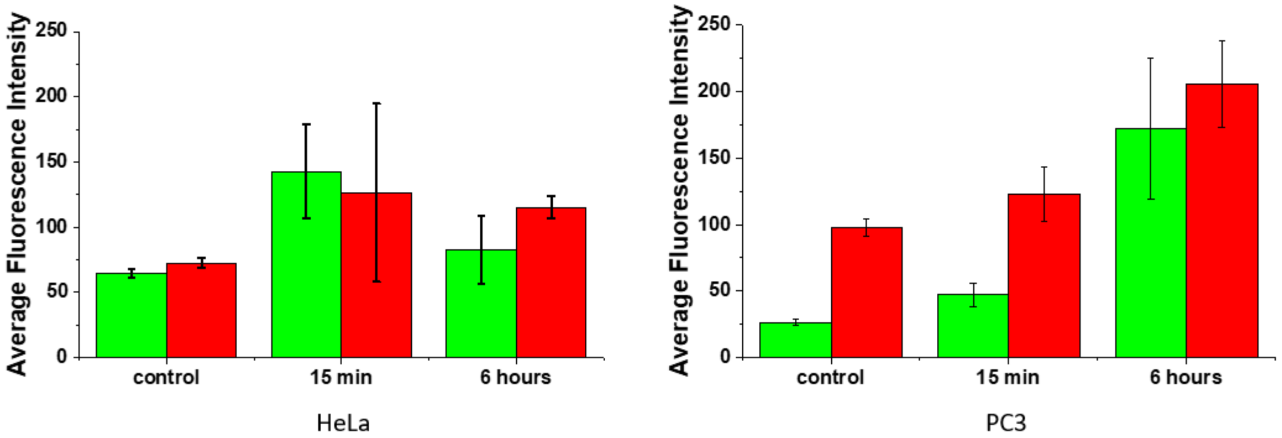

Figure S84. Average fluorescence intensities of HeLa and PC3 cells treated with 1%DMSO (controls, 15 min incubation) vs. cells incubated with 10 µg/mL $Y_{0.9}Eu_{0.1}VO_4@SiO_2$ for 15 min and similarly, for 6 hours incubation times (37 ºC).

General Cell culturing methods for fluorescence imaging

HeLa and PC-3 cell lines were purchased from American type culture collection (ATCC). Cells were grown as monolayers in T75 tissue culture flasks and cultured in Eagle's Minimum Essential Medium (EMEM) for HeLa and Roswell Park Memorial Institute medium (RPMI) for PC-3, 1% L-glutamine (200 mM), 0.5% penicillin/streptomycin (10 000 IU mL-1/10 000 mg mL-1). Cells were cultured at 37 °C in a humidified atmosphere of 5% CO_2 in air and split once 70% confluence had been reached, using the corresponding cell medium. All steps were performed in absence of phenol red. Once cells reached more than 70% confluence, the supernatant containing dead cell matter and excess protein was aspirated. The live adherent cells were then washed with 10 mL of phosphate buffer saline solution twice to remove any remaining media. Cells were incubated in 3 mL of trypsin solution (0.25% trypsin) for 5 to 7 min at 37 °C.

After trypsinisation, 6 mL of medium containing 10% serum medium was added to inactivate the trypsin and the solution was centrifuged for 5 min (1000 rpm, 25 °C). The supernatant liquid was aspirated and 5 mL of serum medium was added to the cell matter left behind. Cells were counted using a haemocytometer and then seeded as appropriate.

For microscopy, cells were seeded into glass-bottomed Petri dishes and incubated for 12 h for HeLa and 24 h for PC-3 to ensure adhesion. Cells were plated in 35 mm uncoated 1.5 mm thick glass-bottomed dishes as 3 × 10^5 cells per dish and incubated for at least 24 h prior to imaging experiment. Once cells attached firmly, cells were washed with 990 μL Hank's Balanced Salt Solution (HBSS) five times and refilled with 990 μL of serum-free medium (SFM), then in each case, an aliquot of 10 μL of the nanoparticulate material (generally as 1 mg/mL stock dispersion in DMSO) was added. Cells were incubated with compounds for 15 minutes, 1 h, 6h or over-night at 37 °C, or longer, as required. Afterwards, cells were washed with 990 μL Hank's Balanced Salt Solution (HBSS) three times to rinse any remaining probe traces from the medium and 990 μL of SFM was added.

Once the cell dish was ready for the single photon confocal fluorescence imaging, cells were excited at 405 nm, 488 nm and 561 nm wavelength, then at each wavelength, there were five images captured namely a merged image, image between 420 and 480 nm wavelength, image between 516 and 530 nm wavelength, image between 615 and 650 nm wavelength and Differential Interference Contrast (DIC) image.

Cellular Viability Tests

Standard MTT assays of HeLa cells treated with $Y_{0.9}Eu_{0.1}VO_4$@SiO_2 composite were performed in order to investigate the effect of silica encapsulation on the cellular viability. The results demonstrate that encapsulation of $Y_{0.9}Eu_{0.1}VO_4$ NPs within a silica shell improves the *in vitro* biocompatibility.

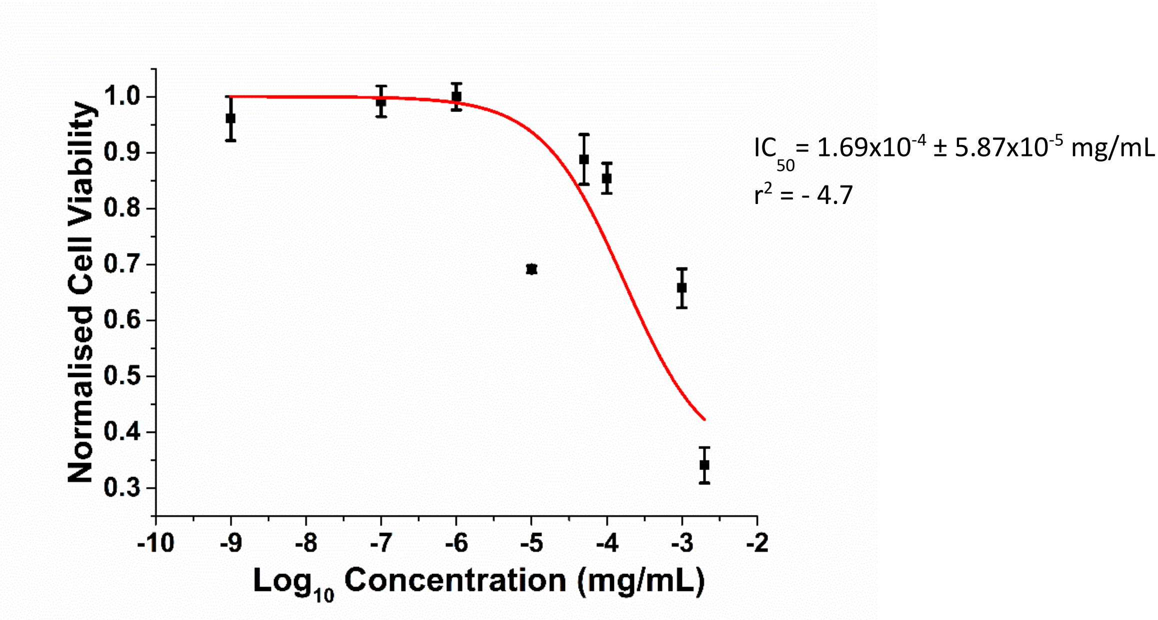

Figure S85. Dose response curve for $Y_{0.9}Eu_{0.1}VO_4$@SiO_2 composite. Error bars stand for standard error calculated from the twelve repeats.

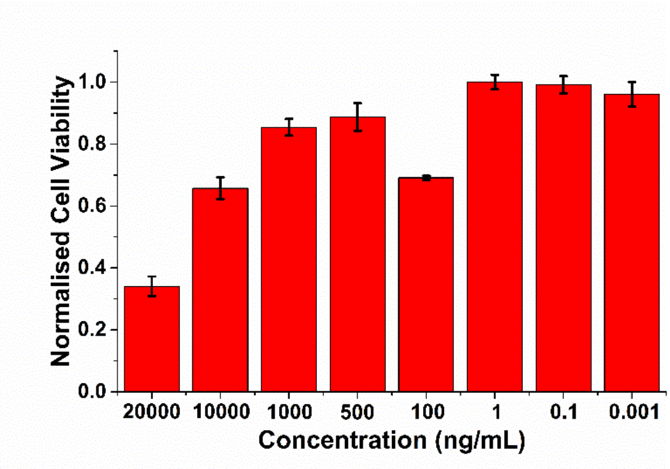

Figure S86. Normalised cell viability in in HeLa cells treated with 1pg/mL – 20 µg/mL $Y_{0.9}Eu_{0.1}VO_4@SiO_2$, cells seed and grow for 48 hours at 37 ºC, $Y_{0.9}Eu_{0.1}VO_4@SiO_2$ was incubated for 48 hours at 37 ºC, 5 mg/mL MTT reagent was incubated for 3 hours. Error bars stand for standard error calculated from the twelve repeats.

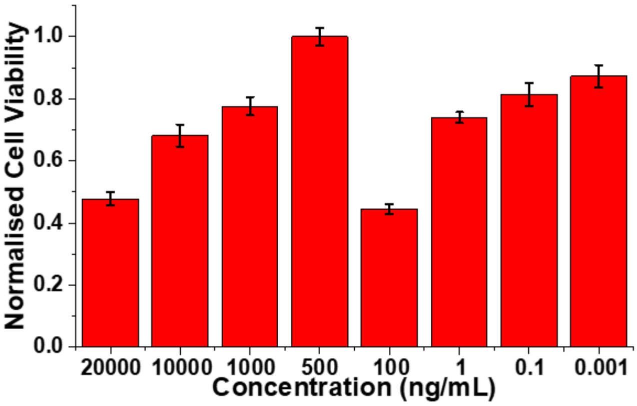

Figure S87. Normalised cell viability in in PC3 cells treated with 1pg/mL – 20 µg/mL $Y_{0.9}Eu_{0.1}VO_4@SiO_2$, cells seed and grow for 48 hours at 37 ºC, $Y_{0.9}Eu_{0.1}VO_4@SiO_2$ was incubated for 48 hours at 37 ºC, 5 mg/mL MTT reagent was incubated for 3 hours. Error bars stand for standard error calculated from the twelve repeats.

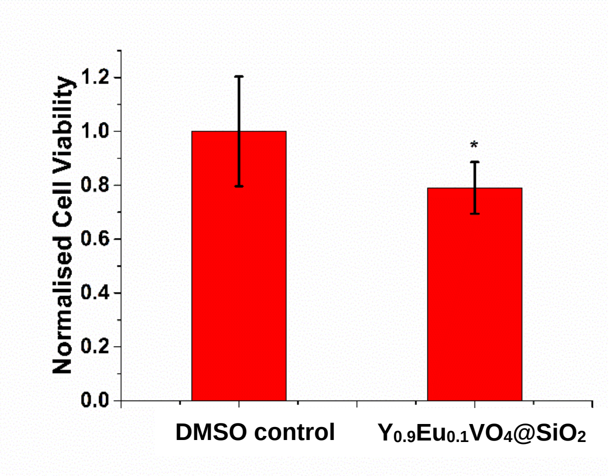

Figure S88. Normalised cell viability in HeLa cells treated with $Y_{0.9}Eu_{0.1}VO_4@SiO_2$ composite for 48 hours. Significance in difference between two groups were tested by Student *t* test. The asterisk marks a significant difference at the level of $p < 0.05$.

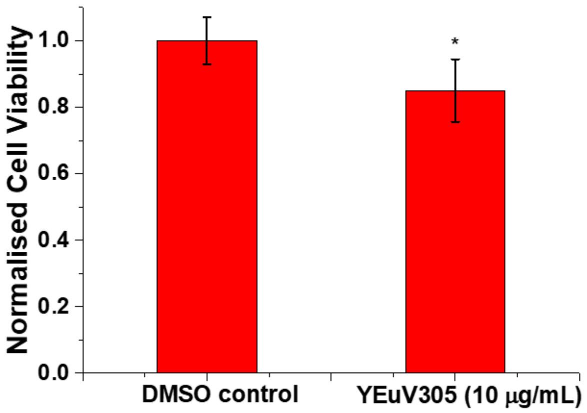

Figure S89. Normalised cell viability in PC3 cells treated with $Y_{0.9}Eu_{0.1}VO_4@SiO_2$ composite for 48 hours. Significance in difference between two groups were tested by Student *t* test. The asterisk marks a significant difference at the level of $p < 0.05$.

References

(1) Townsend, J. B.; Shaheen, F.; Liu, R.; Lam, K. S. Jeffamine Derivatized TentaGel Beads and Poly(Dimethylsiloxane) Microbead Cassettes for Ultrahigh-Throughput in Situ Releasable Solution-Phase Cell-Based Screening of One-Bead-One-Compound Combinatorial Small Molecule Libraries. *J. Comb. Chem.* **2010**, *12* (5), 700–712. https://doi.org/10.1021/cc100083f.
